# Estimating the True Effect of Lifestyle Risk Factors for Myopia: A Longitudinal Study of UK Children

**DOI:** 10.1167/tvst.13.11.10

**Published:** 2024-11-13

**Authors:** Jeremy A. Guggenheim, Rosie Clark, Anna Pease, Peter S. Blair, Cathy Williams, on behalf of the UK Biobank Eye and Vision Consortium

**Affiliations:** https://orcid.org/0000-0001-5164-340X; https://orcid.org/0000-0003-1247-4636; https://orcid.org/0000-0002-3472-1047; https://orcid.org/0000-0002-7832-8087; https://orcid.org/0000-0002-9133-2021; 1 School of Optometry & Vision Sciences, Cardiff University, Cardiff, UK; 2 Centre for Academic Child Health, Population Health Sciences, Bristol Medical School, University of Bristol, Bristol, UK; 3 Epidemiology and Statistics, Bristol Population Health Sciences, Bristol Medical School, University of Bristol, Bristol, UK

**Keywords:** myopia, time outdoors, educational attainment, sleep, instrumental variable, ALSPAC

## Abstract

**Purpose:**

Lifestyle risk factors are implicated in driving the current surge in myopia prevalence yet, paradoxically, known risk factors explain little of the variation in refractive error in the population. Here, we applied “instrumental variable” (IV) methods designed to avoid reverse causation and decrease confounding bias, to gauge lifestyle risk factor effect sizes.

**Methods:**

Three myopia risk factors (time outdoors, time reading, and sleep duration) were assessed in participants of the Avon Longitudinal Study of Parents and Children: a cross-sectional sample of 2302 children aged 15 years old and a longitudinal sample of 3086 children followed from age 7 to 15 years. Seven IVs were considered jointly: dog ownership, cat ownership, bedtime variability, birth order, and polygenic scores quantifying genetic predisposition to spend additional time outdoors, years in fulltime education, and time asleep overnight.

**Results:**

Risk factor effect sizes were 4-fold to 9-fold higher in the IV analyses compared with conventional regression analyses. In IV analyses, one extra hour spent outdoors every day during childhood was associated with a shift toward hyperopia of +0.53 to +0.94 diopters (D), whereas 1 extra hour spent reading every day was associated with a shift toward myopia of −0.44 to −0.88 D. There was inconsistent evidence that sleep duration influenced refractive error.

**Conclusions:**

Myopia risk factor effects were underestimated up to 9-fold in conventional analyses in this sample, compared with IV analyses.

**Translational Relevance:**

We speculate that the effects of lifestyle risk factors for myopia have been underestimated in past studies.

## Introduction

The prevalence of myopia has risen steadily, which points to modern lifestyle factors as triggers or drivers for myopia, even if genetics is known to be important in determining an individual's initial susceptibility and rate of progression.^1^^–^^5^ Myopia has become a public health concern.^5^^,^^6^

Longitudinal cohort studies of children have been successful in identifying environmental risk factors for myopia. A protective (negative) association between myopia and time outdoors was highlighted in the Sydney Myopia Study^7^ and confirmed in subsequent randomized controlled trials (RCTs).^8^^,^^9^ A potentially risk-increasing (positive) association between myopia and measures of educational intensity such as academic achievement and after school class attendance has also been widely observed.^10^ However, in the absence of RCTs examining the impact of additional schooling, support for a causal role of education has relied on noninterventional methods such as Mendelian randomization^11^^–^^13^ and regression discontinuity.^14^^–^^16^ More recently, some studies have reported a potentially risk-increasing association between myopia and shorter sleep duration.^17^ Like education, sleep duration is a trait that is not readily amenable to examination in an RCT.

**Figure 1.**
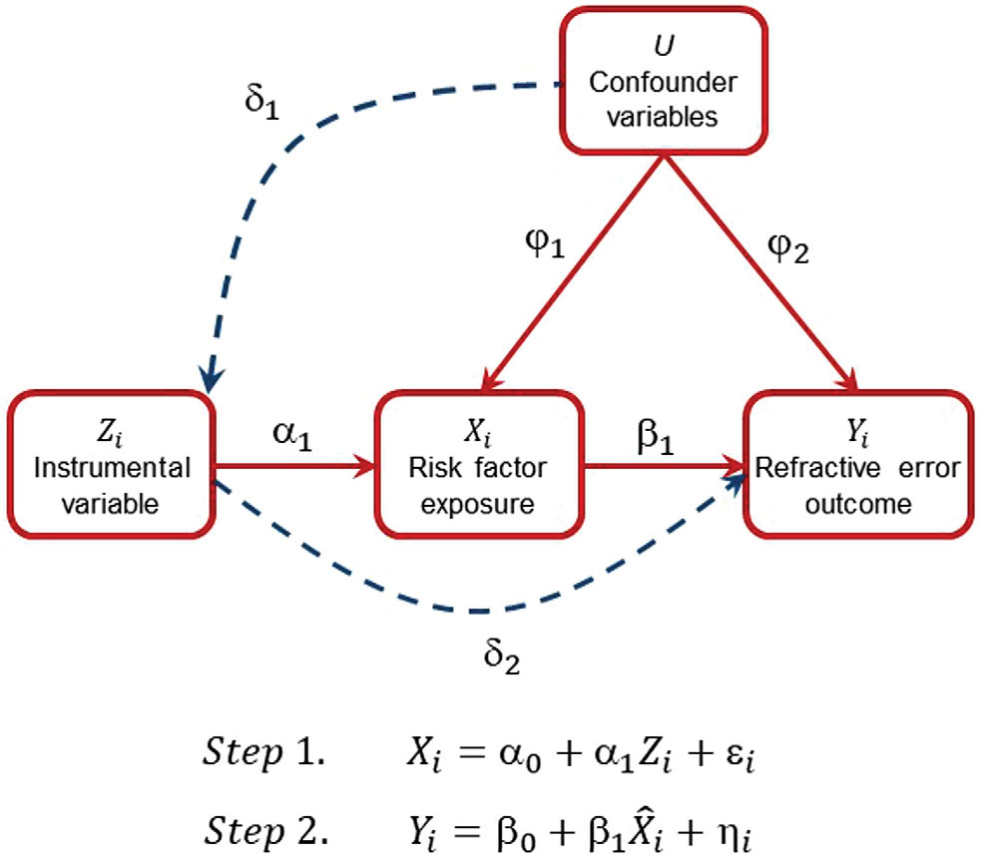
IV analysis. Directed acyclic graph showing assumed causal paths from an IV Z to a risk factor *X* (α_1_), from the risk factor to refractive error *Y* (β_1_), and from confounder variables *U* to the risk factor (φ_1_) and to refractive error (φ_2_). The aim of the analysis was to estimate β_1_ without bias from φ_1_ and φ_2_. The validity of an IV analysis relies on three assumptions: (i) Relevance, a change in the level of the IV causes a robust change in risk factor exposure (α_1_ > 0); (ii) Exclusion restriction, the only path through which the IV affects refractive error is via the risk factor (δ_2_ = 0); and (iii) Exchangeability, there is no causal path between the IV and the confounders (δ_1_ = 0). IV analyses can be performed using a two-step procedure. In step 1, *Z* is regressed on *X*. In step 2, the fitted values of *X* ($\hat{X}$) are regressed on *Y*. For example, consider dog ownership as an IV for time spent outdoors. Step 1 would estimate the difference in time spent outdoors by children whose families do vs. do not own a dog, and step 2 would estimate the shift in refractive error associated with this change in time outdoors. The validity of assumption (i) can be tested by calculating the F-statistic quantifying the strength of association between the IV and the risk factor in step 1.

The challenge of designing RCTs to investigate the role of education and sleep in myopia development reflects two general limitations of RCTs: their high financial cost and the high demands they place on study participants. Most alternatives to RCTs, such as Mendelian randomization and regression discontinuity, are forms of “instrumental variable” (IV) analysis.^18^ Although providing much weaker evidence of causation compared with RCTs, IV methods nevertheless aim to provide causal insight by overcoming the key limitations of “observational” study designs: namely, reverse causation and residual confounding (Box 1). As an example of an IV analysis, Evans and Ringel^19^ used the level of taxes on cigarettes as an IV to examine if reducing the proportion of women who smoked during pregnancy would lead to improved birth outcomes. They found that the rate of smoking during pregnancy did indeed decrease when cigarette taxes were increased and that this led to an increase in the average birth weight.^19^ The theoretical basis of IV methods relies on strong assumptions that are difficult or impossible to verify in practice (Fig. 1). Nevertheless, because IV methods are often subject to different sources of bias compared with observational study designs, they can provide independent evidence for or against a specific risk factor–outcome relationship.^20^^,^^21^

Box 1. **Box 1. Reverse Causation and Residual Confounding in Epidemiology Studies**

Reverse causation describes the situation when an outcome is a cause of a putative risk factor, rather than the converse. As an example, poor mental health may lead to social isolation, which may further worsen mental health: here, the outcome “mental health” is both a cause and a consequence of the risk factor “social isolation.” An observational regression analysis of mental health and social isolation could, therefore, yield an upwardly biased estimate of the causal effect of social isolation on mental health by not accounting for the reciprocal nature of this relationship. Confounding describes the situation when an outcome and a putative risk factor have a common cause. As an example, educational attainment is a common cause of a (lesser) likelihood to smoke and of (higher) household income. Thus, an observational regression analysis of household income on number of cigarettes smoked daily may provide a biased estimate of the causal effect of smoking, resulting from confounding by education level. Residual confounding occurs when a regression analysis seeks to statistically adjust for the effects of a confounder, but where the adjustment is imperfect: this can occur when the confounder is measured with error or if the nature, for example, linearity, of the confounding relationships assumed by the statistical model does not match the true form of these relationships. Returning to the example of cigarette smoking and household income, then if years of education is included as an index of educational attainment in a regression analysis of household income on number of cigarettes smoked daily, this might only partially account for the true confounding effect of education, leaving some (residual) confounding unaccounted for. IV methods aim to decrease the bias from reverse causation, unmeasured confounding, and residual confounding, by leveraging information from a new variable (the “IV”) that is a discrete cause of the putative risk factor. For example, consider the price of cigarettes as an IV for the number of cigarettes smoked daily. With reference to Figure 1, Step 1 would estimate the reduction in the number of cigarettes smoked daily in a study population before vs. after an increase in the price of cigarettes, whereas step 2 would estimate the shift in household income associated with this change in the number of cigarettes smoked daily.

In large-scale studies of myopia development, observational regression models account for only a few percent of the intersubject variation, suggesting that many risk factors and confounders have yet to be discovered or that high levels of residual confounding are present.^22^^–^^24^ In the current work, we compared the results obtained with observational regression analyses and IV regression analyses that incorporated both genetic and nongenetic IVs. We evaluated three different myopia risk factors: time outdoors, time reading, and sleep duration.

## Methods

### Study Sample and Refractive Error (Outcome) Measurement

The Avon Longitudinal Study of Parents and Children (ALSPAC) is an on-going birth cohort study based in the UK, designed to examine a diverse range of lifestyle and genetic risk factors relating to health and well-being.^25^^,^^26^ Pregnant women resident in Avon, UK, with expected dates of delivery between April 1, 1991, and December 31, 1992, were invited to take part in the study. The initial number of pregnancies enrolled was 14,541; this increased to 15,447 pregnancies after an attempt to bolster recruitment when eligible children were aged 7 years. Of these pregnancies, a total of 14,901 children were alive at 1 year of age. Ethical approval for the study was obtained from the ALSPAC Ethics and Law Committee and the Local Research Ethics Committees (ALEC; IRB00003312; registered as “U Bristol IRB #1” on the Office of Human Research Protections database). Informed consent for the use of data collected via questionnaires and clinics was obtained from participants following the recommendations of the ALEC at the time. Detailed information describing how the confidentiality of the cohort is maintained can be found at http://www.bristol.ac.uk/alspac/researchers/research-ethics/. At regular intervals throughout childhood, parents and study participants completed questionnaires about their lifestyle, home environment, and behavior. The ALSPAC website contains details of all the data that are available through a fully searchable data dictionary and variable search tool available at http://www.bristol.ac.uk/alspac/researchers/our-data/.

All participants were invited to a research clinic at the age of 7 years and then annual follow-up visits. Noncycloplegic autorefraction was performed with a Canon R50 instrument (Canon USA, Inc., Lake Success, NY) at the research clinic visits scheduled when the children were aged 7, 10, 11, 12, and 15 years. The refractive error of the participant was calculated as the spherical equivalent (sphere power plus 0.5 × cylinder power), averaged between the two eyes.^27^ For 346 participants who attended the 15-year clinic visit, the noncycloplegic subjective refraction spectacle prescription from the participant's optometrist was available.^28^ The correlation between the noncycloplegic subjective refraction and the noncycloplegic autorefraction measurements was 0.87 and the mean difference between the measurements was −0.22 ± 0.84 diopters (D).

### Time Reading, Time Outdoors, and Sleep Duration (Exposure Variables)

Time outdoors, in discretized categories of hours per day, was reported in parent-completed questionnaires when the study children were aged 3.0, 4.5, 5.5, 6.5, and 8.5 years, as well as in a child-completed questionnaire at age 14.0 years. Time reading books, in discretized categories of hours per day, was reported in parent-completed questionnaires when the study children were aged 4.5, 5.5, 6.5, and 8.5 years, and the child-completed questionnaire at age 14.0 years. Sleep duration, in units of hours per day, was calculated from parent-completed questionnaire responses that asked the time at which study participants went to bed and awoke. The questionnaires were completed when the study children were aged 3.5, 5.0, 6.0, 7.0, 9.5, and 11.5 years. For the time outdoors and time reading books questionnaire items, the responses at some ages were recorded separately for school weekdays, weekend days, and school holidays, as well for these periods in the summer and the winter. The response values were converted from categorical to numerical values (hours per day) and then the weighted average was calculated: for example, nonholiday value = (school weekday value × 5/7) + (weekend value × 2/7). The relationship between demographic characteristics and the time ALSPAC participants spent outdoors, and reading, has been reported previously.^29^^,^^30^ Code to reproduce the derivation of these variables can be found in Supplementary Note 1.

### Nongenetic IVs

IVs were selected based on a literature review of the use of IVs to study sleep duration, time spent outdoors and education. The ownership of a dog by the study participant's family was used as an IV for time outdoors.^31^ Dog ownership is known to promote time outdoors; in the UK, most dog owners take their dog for a walk outside once or twice per day.^31^^,^^32^ We reasoned that some children would take the family dog for a walk or accompany a parent taking the family dog for a walk. This factor would, on average, lead to a slightly greater amount of time spent outdoors for children in families who owned a dog. Ownership of a cat by the study participant's family was used as a negative control IV. Cat ownership was not expected to be associated with additional time outdoors, because children in the UK rarely, if ever, take the family cat for a walk outside. Dog and cat ownership were reported in parent-completed questionnaires when participants were aged 7 and 10 years. The dog ownership variable was coded as 0 or 1, with 1 indicating ownership at age 7 years and/or 10 years, and 0 indicating nonownership at both ages. Cat ownership was coded in the same way. Birth order was used as an IV for time reading books, because birth order is associated with early educational attainment.^33^^–^^35^ Birth order was coded as an integer between 1 and 4. It was calculated as 1 plus the participants’ number of older siblings, as reported by the mother when the study child was 18 months old. This value was truncated to four for birth orders of five and above, owing to the low numbers of higher birth orders. Bedtime variability was used as an IV for sleep duration. Bedtime variability is associated with sleep duration in adults.^36^^,^^37^ Bedtime variability was defined as the standard deviation (SD) of the parent-reported study child's bedtime at the ages of 3.5, 5.0, 6.0, and 7.0 years (for weekdays) and 7.0 years (for weekend days). If data were missing, the SD of the bedtimes across the available time points between age 3.5 and 7.0 years was calculated.

### Genetic IVs

A polygenic score (PGS) for sleep duration in adulthood was tested as an IV for children's sleep duration (although prior work suggested this PGS would have limited instrument strength).^38^ A PGS for time outdoors in adulthood was used as an IV for children's time spent outdoors, as reported in our previous work.^39^ A PGS for years of full-time education was used as an IV for children's time spent reading books.^39^ To calculate each PGS, single nucleotide polymorphism (SNP)–weighting factors were obtained in a sample of 322,643 participants of European ancestry from the UK Biobank,^40^ who had information available for self-reported sleep duration (UK Biobank data field #1160), time spent outdoors in summer (UK Biobank data field #1050), and age completed full-time education (UK Biobank data fields #845 and #6138).^12^^,^^39^ SNP regression coefficients were obtained for approximately 1.1 million HapMap3 variants^41^ in a genome-wide association study for each trait using the ‘—predBetasFile’ function for an infinitesimal model in BOLT-LMM.^42^ The covariates included were: age, age squared, sex, genotyping array, the first 10 ancestry principal components, ‘northing’ geographical coordinate of place of birth, ‘easting’ geographical coordinate of place of birth, and a dummy variable for UK Biobank assessment center. A genetic relatedness matrix was used to account for kinship. PGSs were calculated for ALSPAC study participants using the ‘—score’ function of PLINK v1.9.^43^ PGSs were standardized to have a mean of 0 and a SD of 1.

### Statistical Analyses

Analyses were carried out with R (version 4.3.1). Spearman correlations were calculated using the ‘cor.test’ function. Plots were obtained with the *ggplot2* package (version 3.4.4) and *forestploter* package (version 1.1.1). Ordinary least squares and IV regression models for the outcome ‘refractive error measured at age 15 years’ in the cross-sectional sample were fitted using the *fixest* package (version 0.11.2). IV and non-IV panel data models for the analysis of the longitudinal sample were fitted with the *plm* package (version 2.6.3). R code for the analyses is provided in Supplementary Note 2. Summary data for normally and non-normally distributed variables are presented as mean and 95% confidence interval or the median and interquartile range (IQR), respectively.

Sleep duration (hours per night), time outdoors (hours per day), or time reading books (hours per day) were first considered separately as predictor variables and then included together in a combined model. Models were fitted both with and without the inclusion of covariates to adjust for gender, maternal age and Townsend Deprivation Index quintile (a 5-interval index of UK socioeconomic status). In models that included polygenic risk scores, the first three genetic ancestry principal components were included as additional covariates. The IV models were fitted with either one or all seven IVs (dog ownership, cat ownership, birth order, bedtime variability, PGS for sleep duration, PGS for time outdoors, or PGS for full-time education). In the panel data models, age was fitted as a fixed effect and age at each clinic visit (ages 7, 10, 11, 12, and 15 years) nested within subject was fitted as a random effect. The panel data regressions implemented the random effects model of Nerlove,^44^ with the Balestra and Varadharajan–Krishnakumar IVs estimator.^45^ The predictor variable(s) were assumed to have no effect at baseline, whereas their effects were assumed to increase linearly over the age range of 7.5 to 15.5 years (via inclusion of a linear predictor-by-age interaction for each predictor). IVs were included as discrete terms as well as IV-by-age interaction terms. Age, age^2 and age^3 were included as fixed effects in all panel data models to account for the nonlinear association of refractive error with age. Covariate-adjusted models also included fixed effects terms for gender, maternal age, Townsend Deprivation Index quintile, and the first three genetic ancestry principal components.

## Results

### Demographic Characteristics of the Study Population

ALSPAC participants were invited to a series of five research clinic visits when they were aged 7, 10, 11, 12, and 15 years old. Table 1 lists the demographic characteristics of the 8724 ALSPAC participants who attended at least one research clinic visit and who also had genotype information available. To evaluate risk factors associated with refractive error at age 15 years, we used a cross-sectional sample of 2302 participants who attended the fifth clinic visit, and to evaluate risk factors associated with refractive error trajectory between age 7 years and 15 years, we used a longitudinal sample of 3086 participants who attended at least three of the five visits. Compared with the full sample of 8724 children, individuals in the cross-sectional and longitudinal samples had a higher proportion of females, slightly older mothers, and were from less deprived families; however, the differences were small (Table 1).

**Table 1.** Demographic Characteristics of the Study Samples

Trait^a^	Full Sample^b^	Cross-Sectional Sample	*P* Value^c^^,^^d^	Longitudinal Sample	*P* Value^d^^,^^e^
Sample size	8724	2302	–	3086	–
Female (%)	4262 (48.9%)	1242 (54.0%)	<0.001	1614 (52.3%)	0.001
No. of visits [median (25th,75th)]	4.00 (1.00, 5.00)	5.00 (5.00, 5.00)	<0.001	5.00 (4.00, 5.00)	<0.001
Townsend deprivation index quintile ^f^ [median (25th,75th)]	3.00 (1.00, 4.00)	2.00 (1.00, 4.00)	<0.001	2.00 (1.00, 4.00)	<0.001
Maternal age [median (25th,75th)] (years)	28.00 (25.00, 32.00)	29.00 (27.00, 32.00)	<0.001	29.00 (26.00, 32.00)	<0.001
Birth order [median (25th,75th)]	2.00 (1.00, 2.00)	2.00 (1.00, 2.00)	0.017	2.00 (1.00, 2.00)	0.337
Sleep duration at age 9.5 years [median (25th,75th)] (hours/day)	10.50 (10.00, 10.86)	10.50 (10.00, 10.86)	0.828	10.50 (10.00, 10.86)	0.717
Time outdoors at age 8.5 years [median (25th,75th)] (hours/day)	1.96 (1.69, 2.25)	1.96 (1.69, 2.25)	0.058	1.96 (1.69, 2.25)	0.528
Time reading at age 8.5 years [median (25th,75th)] (hours/day)	0.50 (0.50, 1.15)	0.50 (0.50, 1.15)	0.114	0.50 (0.50, 1.03)	0.943
Bedtime variability [median (25th,75th)] (SD units)	0.50 (0.35, 0.71)	0.50 (0.35, 0.67)	0.097	0.50 (0.35, 0.71)	0.522
Dog ownership by family (%)	2016 (35.5%)	752 (32.7%)	0.019	1028 (33.3%)	0.046
Cat ownership by family (%)	2497 (43.8%)	1023 (44.4%)	0.637	1344 (43.6%)	0.819
PGS for sleep duration [mean (SD)] (SD units)	0.00 (1.00)	0.01 (0.98)	0.851	0.01 (0.98)	0.656
PGS for time outdoors [mean (SD)](SD units)	0.00 (1.00)	−0.12 (1.00)	<0.001	−0.09 (1.00)	<0.001
PGS for full-time education [mean (SD)] (SD units)	0.01 (1.00)	0.21 (1.00)	<0.001	0.15 (0.99)	<0.001
Age at 7-year clinic [mean (SD)] (years)	7.53 (0.31)	–	–	7.45 (0.14)	<0.001
Age at 10-year clinic [mean (SD)] (years)	10.64 (0.25)	–	–	10.59 (0.20)	<0.001
Age at 11-year clinic [mean (SD)] (years)	11.74 (0.23)	–	–	11.71 (0.20)	<0.001
Age at 12-year clinic [mean (SD)] (years)	12.80 (0.23)	–	–	12.78 (0.21)	<0.001
Age at 15-year clinic [mean (SD)] (years)	15.45 (0.32)	15.39 (0.24)	<0.001	15.39 (0.24)	<0.001
Refractive error at 7-year clinic [median (25th,75th)] (D)	0.12 (−0.19, +0.44)	–	–	0.12 (−0.19, +0.44)	0.422
Refractive error at 10-year clinic [median (25th,75th)] (D)	0.00 (−0.31, +0.31)	–	–	0.00 (−0.31, +0.31)	0.567
Refractive error at 11-year clinic [median (25th,75th)] (D)	0.00 (−0.38, +0.31)	–	–	−0.06 (−0.38, +0.25)	0.383
Refractive error at 12-year clinic [median (25th,75th)] (D)	−0.12 (−0.50, +0.19)	–	–	−0.12 (−0.50, +0.19)	0.393
Refractive error at 15-year clinic [median (25th,75th)] (D)	−0.25 (−0.69, +0.06)	−0.31 (−0.69, +0.06)	0.695	−0.31 (−0.69, +0.06)	0.583

a (25^th^, 75th) refers to the 25th and 75th percentiles.

b Values are for the available sample at each visit, since not all participants attend all five visits and because pet ownership was not known for some families.

c *P* value for comparison of full sample vs. cross-sectional sample.

d Two-sample test for independent proportions; Mann–Whitney *U* test for non-normally distributed variables; Independent samples *t* test for normally distributed variables.

e *P* value for comparison of full sample vs. longitudinal sample.

f A higher Townsend Deprivation Index quintile indicates a greater degree of social deprivation.

The three risk factors of interest—sleep duration, time outdoors, and time reading books—were reported in questionnaires completed by the child's parents or the children themselves. Children's sleep duration across childhood was moderately correlated at adjacent age-points (Spearman *r* ≈ 0.5, *P* < 0.001), but became progressively less well-correlated at more widely spaced age-points (Spearman *r* = 0.2 to 0.3, *P* < 0.001) (Supplementary Fig. S1). Patterns of children's time outdoors and time reading books followed similar trends (Supplementary Figs. S2 and S3). For our main analyses, we focused on the three risk factors when they were measured at 8.5 or 9.5 years of age to provide consistency with previous studies in the ALSPAC cohort and because refractive errors often begin to develop at this age in UK children.^27^^,^^29^^,^^30^^,^^46^ At this age, participants spent a median of 10.5 (IQR, 0.9; range, 7.5-14.3) hours asleep each night, a median of 2.0 (IQR, 0.6; range, 0.4-3.0) hours outdoors per day, and a median of 0.50 (IQR, 0.65; range, 0.0-3.0) hours reading books per day.

### Observational Analyses of the Risk Factors for Myopia

**Figure 2.**
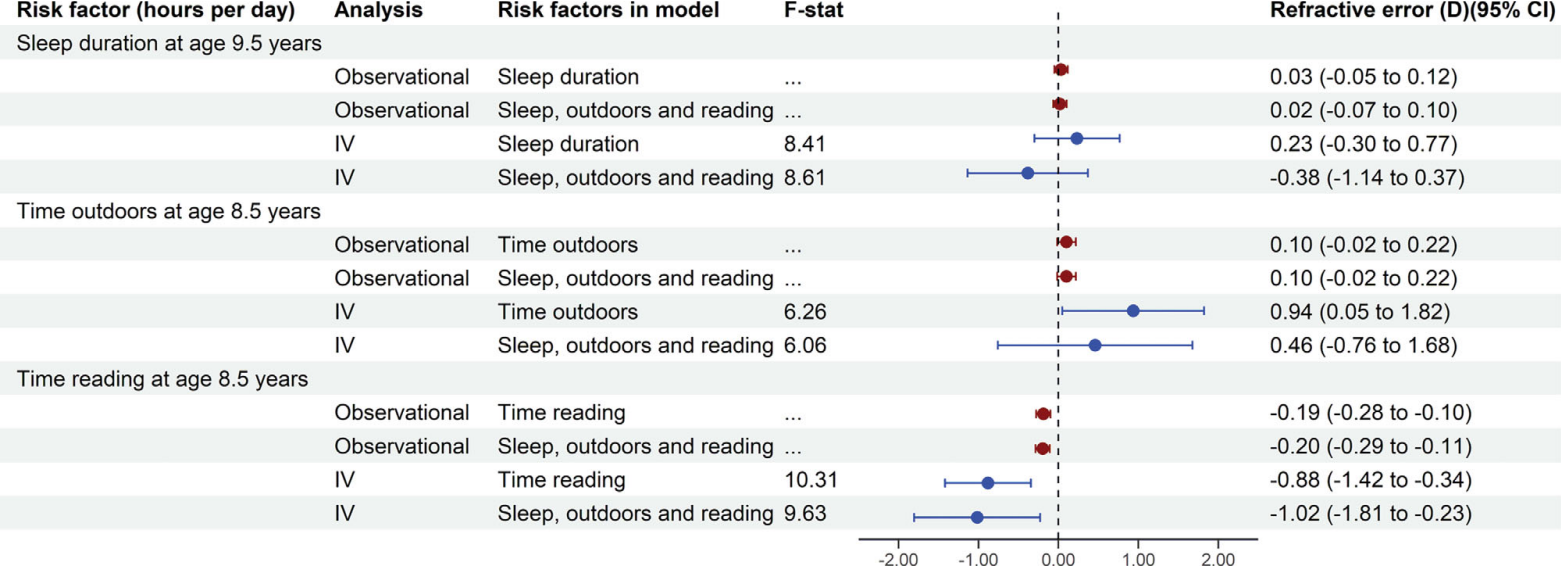
Risk factors associated with the outcome ‘refractive error at age 15 years’ in the cross-sectional sample. Associations are presented in units of *D* per 1 hour of additional activity each day. Risk factor exposure (in units of hours per day) was recorded via questionnaires at the specified age. Risk factors were first assessed one at a time and then together in an analysis that included all three risk factors. The observational analyses adjusted for gender, maternal age, and Townsend Deprivation Index. The IV analyses included seven IVs and adjusted for gender, maternal age, Townsend Deprivation Index, and the first three genetic ancestry principal components. F-stat refers to the F-statistic (combined for all seven IVs) from the first stage of the two step least squares regression; for the model with all three risk factors, the reported F-statistic is conditional on the other risk factors.

The dark red symbols in Figure 2 show the results of standard observational regression analyses in the cross-sectional sample for the outcome ‘refractive error at age 15 years.’ When considering the risk factors one at a time, an additional hour asleep each night was associated with a small, nonsignificant shift toward hyperopia (effect = +0.03 D; *P* = 0.45), as was 1 additional hour spent outdoors each day (effect = +0.10 D, *P* = 0.09). By contrast, 1 additional hour spent reading each day was associated with a small but highly significant shift toward myopia (effect = −0.19 D; *P* < 0.001). The results were very similar when all three risk factors were included together in the model (Fig. 2). The findings from more extensive observational analyses in which the three risk factors were measured at different ages across childhood and with or without adjustment for potential confounders are shown in Supplementary Table S1 and Supplementary Figure S4 and discussed in Supplementary Note S3. The most salient findings from these more detailed analyses were that (i) time reading assessed at age of 5.5 to 14.0 years had an increasingly strong association with future refractive error, (ii) time outdoors over this age range had a consistent, stable association with future refractive error, and (iii) sleep duration at these ages had little association with future refractive error.

**Figure 3.**
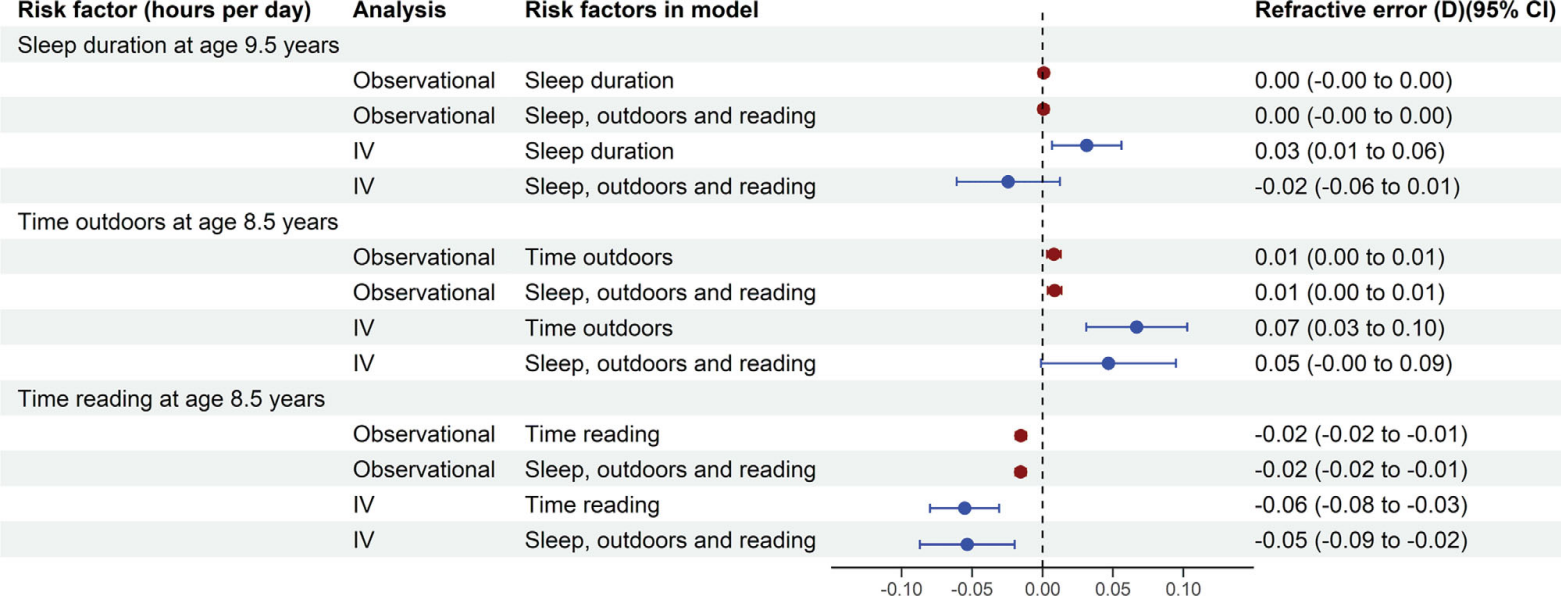
Risk factors associated with the outcome ‘refractive error’ in the longitudinal sample. Associations are presented in units of annual change in refractive error (*D*) per one hour of additional activity each day. Risk factor exposure (in units of hours per day) was recorded via questionnaires at the specified age. Risk factors were first assessed one at a time and then together in an analysis that included all three risk factors. The observational analyses adjusted for gender and Townsend Deprivation Index. The IV analyses included seven IVs and adjusted for gender, maternal age, Townsend Deprivation Index, and the first three genetic ancestry principal components.

As shown in the dark red symbols of Figure 3, observational regression models in the longitudinal sample suggested that one additional hour asleep each night was associated with a very small, nonsignificant annual shift toward hyperopia (effect = +0.001 D; *P* = 0.65). An additional hour spent outdoors each day was associated with a small annual shift toward hyperopia (effect = +0.008 D; *P* = 0.001), while an additional hour spent reading each day was associated with a small annual shift toward myopia (effect = −0.015 D; *P* < 0.001). The results were very similar when all three risk factors were included together in the model and in analyses that did not adjust for the effects of potential confounders (Fig. 3; Supplementary Table S2). Over the 8-year span of the longitudinal study, the cumulative effects of 1 additional hour per day sleeping, being outdoors, or reading were associated with shifts in refractive error of +0.01 D, +0.06 D, and −0.12 D, respectively (calculated as 8 × the annual change in refractive error). These values approximated the effects observed in the cross-sectional analyses.

In summary, the cross-sectional and longitudinal observational regression analyses suggested that an extra hour per day spent reading during childhood was associated with a small shift in refractive error toward myopia (−0.12 to −0.19 D), 1 extra hour per day spent outdoors was associated with a small shift toward hyperopia (+0.06 to +0.10 D) and 1 extra hour asleep was associated with a very small shift toward hyperopia (+0.01 to +0.03 D). Notably, these effect sizes were much lower than the level of myopia often encountered clinically, where values of −3.00 D and −6.00 D are used to classify moderate and high levels of myopia, respectively.^47^

### Evaluation of the IVs

We evaluated a total of seven different variables as potential IVs for the three myopia risk factors. Table 2 lists the first stage F-statistic of each IV for each risk factor, along with its direction of association (the first stage F-statistic is an indicator of the strength of the IV; ideally, an IV should have an F-statistic of >10, which is indicative of a robust association of the IV with the risk factor).^48^ Importantly, the bedtime variability IV was derived from children's bedtimes at age 3.5 to 7.0 years, which was before the age when sleep duration was examined as a risk factor for myopia (age 9.5 years).

**Table 2.** First-Stage F-Statistics Indicating the Strength of Seven Different IVs for Three Myopia Risk Factors

		Risk Factor		
IV	Originally Selected as an IV for the Risk Factor	Sleep Duration at Age 9.5 Years	Time Outdoors at Age 8.5 Years	Time Reading at Age 8.5 Years
Dog ownership by family	Time outdoors	3.55 [+]	6.08 [+]	0.12 [−]
Birth order	Time reading	6.29 [+]	1.57 [+]	27.92 [−]
Bedtime variability	Sleep duration	24.06 [−]	0.42 [+]	0.08 [−]
PGS for time outdoors	Time outdoors	13.86 [+]	25.81 [+]	9.09 [−]
PGS for years spent in education	Time reading	11.17 [−]	27.56 [−]	27.34 [+]
PGS for sleep duration	Sleep duration	1.87 [+]	0.47 [−]	11.52 [−]
Cat ownership by family	(Negative control)	2.40 [−]	0.14 [+]	0.10 [−]

The direction of the association of the IV to the risk factor is shown alongside the F-statistic (positive or negative symbol in square brackets). Calculations were performed in the cross-sectional sample (*n* = 2302) for the outcome ‘refractive error at age 15 years’. Models contained a single myopia risk factor and a single IV, and adjusted for gender, maternal age, Townsend Deprivation Index, and the first three genetic ancestry principal components.

Out of the seven IVs, three were strong IVs for sleep duration, two for time outdoors, and three for time spent reading (Table 2). As seen in Table 3, the magnitude of the IV vs. risk factor associations were all relatively low. For example, family ownership of a dog was associated with children spending an extra 0.05 hours (3 minutes) outdoors each day (*P* = 0.014); a 1-SD increase in the PGS for time outdoors was also associated with children spending an extra 0.05 hours outdoors each day (*P* < 0.001). The IV with the largest effect size was bedtime variability before age 7 years; a 1-SD increase in bedtime variability was associated, on average, with children aged 8.5 years sleeping 0.23 hours (14 minutes) less each night (*P* < 0.001). IV vs. risk factor associations of low magnitude are typical of IV studies, hence the need for large sample sizes.

**Table 3.** Association of Each IV With Each Myopia Risk Factor

	Sleep Duration at Age 9.5 Years (Hours per Night)			Time Outdoors at Age 8.5 Years (Hours per Day)			Time Reading at Age 8.5 Years (Hours per Day)		
Test Variable	BETA	95% Confidence Interval	*P* Value	BETA	95% Confidence Interval	*P* Value	BETA	95% Confidence Interval	*P* Value
Dog ownership by family^a^	0.05	(−0.00 to 0.11)	0.060	0.05	(0.01 to 0.09)	0.014	−0.01	(−0.06 to 0.04)	0.734
Birth order^b^	0.04	(0.01 to 0.07)	0.012	0.01	(−0.01 to 0.04)	0.211	−0.08	(−0.11 to −0.05)	<0.001
Bedtime variability^c^	−0.23	(−0.32 to −0.14)	<0.001	0.02	(−0.04 to 0.09)	0.518	−0.01	(−0.10 to 0.07)	0.784
PGS for time outdoors^c^	0.05	(0.02 to 0.07)	<0.001	0.05	(0.03 to 0.06)	<0.001	−0.04	(−0.06 to −0.01)	0.003
PGS for years spent in education^c^	−0.04	(−0.07 to −0.02)	0.001	−0.05	(−0.07 to −0.03)	<0.001	0.06	(0.04 to 0.09)	<0.001
PGS for sleep duration^c^	0.02	(−0.01 to 0.04)	0.172	−0.01	(−0.02 to 0.01)	0.493	−0.04	(−0.07 to −0.02)	0.001
Cat ownership by family^a^	−0.04	(−0.09 to 0.01)	0.121	0.01	(−0.03 to 0.04)	0.705	−0.01	(−0.05 to 0.04)	0.756

A total of 21 (3 × 7) analyses were performed. In each analysis, one risk factor (*y*; dependent variable) was regressed on one test variable (*x*; independent variable), in the cross-sectional sample (*n* = 2302). Gender, maternal age, Townsend Deprivation Index, and the first three genetic ancestry principal components were included as covariates.

a Reference group = no pet.

b Reference group = first born child.

c Per 1-SD increase.

Furthermore, many of the IVs captured effects relating to more than one risk factor and were correlated with one another (Supplementary Table S5). Supplementary Figures S5, S6, S7, and S8 and Supplementary Note S4 report and discuss these IV evaluations in greater detail. This latter attribute would invalidate them as standalone IVs and instead implied that they would perform better when used in combination with other IVs: because each IV had a different pattern of association with the three myopia risk factor, the use of multiple IVs would allow these differing directions of association to be used to distinguish the independent effect of each risk factor. Hence, we included all seven IVs jointly in the subsequent IV analyses.

### IV Analyses of the Risk Factors for Myopia

The results of IV regression analyses in the cross-sectional sample are shown as the blue symbols of Figure 2 and in Supplementary Table S3. Of the three risk factors, sleep duration was associated with a small, nonsignificant shift in refractive error at age 15 years. Notably, the direction of association reversed in the single risk factor model compared with the model with all three risk factors (effect = +0.23 D [*P* = 0.39] in the single risk factor model; effect = −0.38 D [*P* = 0.32] in the model with all three risk factors). This switch in direction may have arisen if, in the single risk factor model, the IVs inadvertently captured—and attributed to sleep duration—effects that were actually due to time reading or time outdoors. In the IV analyses that examined the risk associated with time outdoors and time readings, the results were largely similar in the single risk factor models compared with the combined three-risk factor model. In the single risk factor models, an additional hour spent outdoors each day was associated with a +0.94 D shift toward hyperopia (*P* = 0.039), while an additional hour spent reading was associated with a −0.88 D shift toward myopia (*P* = 0.001). In the model that included all three myopia risk factors, the effect estimate for time outdoors was attenuated (and no longer statistically significant; effect = +0.46 D; *P* = 0.46), while the effect estimate for time reading was much the same (effect = −1.02 D; *P* = 0.011). Sensitivity analyses in which just the top three IVs were included for each risk factor, instead of including all seven IVs, produced similar results to the analyses that included all seven IVs (Supplementary Fig. S12).

The F-statistic from the first stage of the IV analyses for the seven IVs combined lay within the range 6.0 to 10.3, indicative of moderate statistical power for these analyses. Accordingly, the 95% confidence intervals of the risk factor effect size estimates were relatively wide. A comparison of the consistency of the IV analysis vs. the observational analysis using the Wu-Hausman test suggested the presence of endogeneity in the observational analyses for time outdoors (*P* = 0.052) and time reading (*P* = 0.008), but not for sleep duration (*P* = 0.39). Such endogeneity (defined as a correlation between the risk factor of interest and the statistical model's error term, that is, unexplained variance) is indicative of bias; hence, the Wu–Hausman test findings imply that the IV models provided more reliable estimates of the effects of time outdoors and time reading than the observational models.

The results of IV regression analyses in the longitudinal sample (blue symbols in Fig. 3) mirrored those for the IV analyses in the cross-sectional sample. A 1 hour increase in sleep duration was associated with an effect in opposing directions in the single risk factor analysis compared with the combined risk factor model (effect = +0.03 D [*P* = 0.012] in the single risk factor model; effect = −0.02 D [*P* = 0.19] in the model with all three risk factors). By contrast, time outdoors and time spent reading had similar effect estimates in the single risk factor and combined risk factor models. One additional hour spent outdoors each day was associated with an annual shift toward hyperopia of +0.07 D (*P* < 0.001), whereas 1 additional hour spent reading was associated with an annual shift toward myopia of −0.06 D (*P* < 0.001). Results were similar with or without adjustment for covariates (Supplementary Table S4). Over the 8-year span of the longitudinal study, the cumulative effects of 1 additional hour per day sleeping, being outdoors, or reading were associated with shifts in refractive error of +0.25 D, +0.53 D, and −0.44 D (calculated as 8 × the annual change in refractive error), respectively.

In summary, the IV regression models suggested that over the 8 years from age 7 to 15 years, an additional hour outdoors every day was associated with a shift toward hyperopia in the range +0.53 D to +0.94 D. One additional hour reading every day was associated with a shift toward myopia in the range −0.44 D to −0.88 D. These effect size estimates for time outdoors and time reading were four times to nine times larger than those from the observational models, with evidence that this was due to invalid modeling assumptions in the observational analyses. Sleep duration was not associated significantly with refractive error in most IV models, and its direction of association varied, with longer sleep duration suggesting a shift toward hyperopia in some analyses, yet a shift toward myopia in others.

## Discussion

The current work yielded four main findings. First, risk factor effect sizes were consistently underestimated for all three risk factors when using standard observational analysis methods that are used conventionally in myopia research. This underestimation was likely due to bias from unmeasured confounders or residual confounding. IV analysis methods produced more reliable estimates of risk factor effect sizes (according to the Wu–Hausman test) and suggested time outdoors and time reading had relatively large impacts on myopia development in UK children: up to a +0.94 D shift toward hyperopia for 1 additional hour spent outdoors each day and a −0.88 D shift toward myopia for 1 additional hour spent reading each day. Second, there was only patchy evidence that sleep duration influenced myopia development. The complex pattern of relationships between sleep duration and other myopia risk factors means that a spurious association between sleep duration and myopia may explain previous reports.^17^ Nevertheless, we cannot rule out that sleep had a small effect on refractive error development in the current study cohort, which we were unable to detect reliably (as discussed elsewhere in this article). Third, we confirmed that the genetic contribution to sleep duration in adulthood is distinct from that in childhood.^38^ Thus, valid mendelian randomization (MR) studies of the effects of differences in children's sleep duration on other traits such as myopia will not be possible until researchers identify SNPs robustly associated with sleep duration in childhood. Regrettably, flawed MR studies of the sleep–myopia relationship have recently been published^49^^,^^50^ that wrongly assumed the sleep duration of adults from samples such as UK Biobank would be indicative of children's sleep behavior. Fourth, genetic predisposition to educational attainment and genetic predisposition to spend time outdoors were both associated with children's sleep duration. These findings are indicative of widespread (horizontal) pleiotropy and suggest that IV and MR studies of sleep duration, educational attainment, and time outdoors may inadvertently capture the effects of multiple exposures, resulting in the underestimation or overestimation of causal effects of the index exposure. The use of multiple IVs, as in the current study, or the use of multivariable MR,^39^ may alleviate some of this bias.

The amount of time children sleep is corrective: the deficit caused by too little sleep during one period can be compensated for by more sleep on subsequent nights.^51^ In a previous analysis of the ALSPAC cohort, few children experienced consistently poor sleep duration over childhood.^52^ In this regard, past studies that have examined markers for sleep problems or sleep quality, rather than sleep duration per se, may be more likely to have revealed causal links to myopia.^17^ Also, it has been reported that children noted by their parents as repeatedly snoring, mouth breathing, or displaying apnea during sleep before age 5 years were more likely to have special educational needs when 8 years old.^53^ Thus, if there are differences in the way children with and without special educational needs are educated, this could potentially confer a higher or lower risk of myopia and lead to an association with sleep problems. Children's sleep duration is associated only weakly with socioeconomic status (although UK children from low-income families tend to have a later bedtime and waking time).^52^ Also, children of older mothers (>35 years) typically sleep less than those of younger mothers.^52^ Because older mothers are more highly educated on average,^54^ and because education and myopia are associated causally, a link between sleep and myopia could occur through the confounding pathway: sleep duration ← maternal age ← education → myopia. Our analyses adjusted for maternal age in an attempt to account for this potential source of bias. However, such adjustment could have overcompensated for children's behaviors that were linked to their mother's level of education.

The current work had several limitations relating to the measurement of refractive error and risk factor exposure, which may have contributed to imprecision and led to bias in estimating causal effects. Refractive error at ALSPAC research clinics was assessed by noncycloplegic autorefraction. Lack of cycloplegia decreases precision and, on average, introduces a small bias toward a more negative refractive error measurement, especially at younger ages.^55^ Time outdoors and time reading were assessed by parent-completed questionnaires. Recent studies comparing questionnaires and personal activity monitors have shown that questionnaire responses can be highly inaccurate.^56^^–^^58^ Parent-completed questionnaires also suffer from recall bias. The ALSPAC questionnaires had coarse-grained response options (e.g., “1–2 hours”), which would have compounded errors in assessing time outdoors and reading. Furthermore, our analyses considered the myopia risk factors at a single time point, rather than taking account of behaviors over the whole of childhood. This factor is important, because the age range when time outdoors and time reading exert their greatest influence on refractive development is not yet known.^29^^,^^59^ In addition, we only considered time outdoors and time reading outside of school hours. Individual children will naturally spend different amounts of time engaged in these activities while at school, yet this source of variation would not have been accounted for in our analyses. Much of the school day is spent indoors, and a high proportion of classroom time may be spent performing near work. Hence, the variation in time outdoors and reading outside of school hours may have been dwarfed by long periods performing these activities at school. If the exposure–outcome relationship for either time outdoors and myopia or time reading and myopia plateaus at higher exposure levels, our failure to account for risk factor exposure during the school day could have had a major impact on our results. Finally, the current study had analytical limitations. We were unable to identify IVs that were specific for each of the three risk factors: instead, most of the IVs were associated with more than one of the risk factors. Also, despite the large sample size of the study cohort, statistical power was limited. This was due to the relatively small and, in some cases, relatively weak associations between the variables used as IVs and the myopia risk factor that they were selected for (Tables 2 and 3). Dog ownership was a notably weak IV for time outdoors (first stage F-statistic = 6.08) and the PGS for sleep duration was a very weak IV for sleep duration (first stage F-statistic = 1.87). The use of multiple IVs only partially mitigated this weak instrument phenomenon: the first stage F-statistic for all IVs combined ranged from 6 to 10 (Fig. 2; Supplementary Table S3). The use of weak IVs can lead to weak instrument bias, in which effect size estimates are biased toward the effect size obtained in a conventional observational regression analysis. Thus, in the current work, weak instrument bias would be expected to lead to an underestimation of the true effects of the myopia risk factors.

In summary, we found evidence that the statistical analysis method conventionally used to study risk factors for myopia underestimated the true causal effects of these risk factors. Specifically, compared with analyses using the conventional method, an IV analysis of the same dataset yielded causal effects that were up to nine-fold larger: 1 additional hour spent outdoors per day was associated with a +0.94 D shift toward hyperopia and 1 additional hour spent reading was associated with a shift toward myopia of −0.88 D. The underestimation of causal effects in the conventional, observational analyses likely resulted from residual confounding or unmeasured confounders. As regards sleep duration, the current work found little evidence to support a role for sleep duration in causing myopia. More generally, a notable finding in the current work was widespread horizontal pleiotropy of genetic variants associated with years of education, time outdoors, and sleep duration. SNPs associated with one trait were typically associated with the other two traits as well. As implemented here, future analyses should consider multivariable IV analysis methods to account for this horizontal pleiotropy.

## Supplementary Material

Supplement 1
