# Supplementary material for: Estimating the True Effect of Lifestyle Risk Factors for Myopia: A Longitudinal Study of UK Children
Source: Transl Vis Sci Technol. 2024 Nov 13;13(11):10. doi: 10.1167/tvst.13.11.10 (PMC11575705; doi:10.1167/tvst.13.11.10)

Estimating the true effect of lifestyle risk factors for myopia: A longitudinal study of UK children

Supplementary Information

Table of Contents

Supplementary Note 1. Code for deriving exposure variables. 3

Supplementary Note 2. Code for fitting models. 14

Supplementary Note S3. Observational associations of sleep duration, time outdoors and time reading at early ages with the outcome, 'refractive error at age 15 years'. 61

Supplementary Note S4. Selection of instrumental variables for each myopia risk factor. 62

Supplementary Table S1. Observational analysis of risk factors associated with the outcome 'refractive error at age 15 years' in the full sample. 63

Supplementary Table S2. Observational analysis of risk factors associated with the outcome 'refractive error' in the longitudinal sample. 65

Supplementary Table S3. Instrumental variable analysis of risk factors associated with the outcome 'refractive error at age 15 years' in the cross-sectional. 66

Supplementary Table S4. Instrumental variable analysis of risk factors associated with the outcome 'refractive error' in the longitudinal sample. 67

Supplementary Table S5. Pearson correlation matrix for the instrumental variables in the cross-sectional sample. 68

Supplementary Figure S1. Correlation of sleep duration across childhood. 69

Supplementary Figure S2. Correlation of time outdoors across childhood. 70

Supplementary Figure S3. Correlation of time reading across childhood. 71

Supplementary Figure S4. Observational associations of sleep duration, time outdoors and time reading at early ages with the outcome, 'refractive error at age 15 years'. 72

Supplementary Figure S5. Relationship between sleep duration at age 9.5 years with demographic characteristics and instrumental variables. 73

Supplementary Figure S6. Observational associations between seven different instrumental variables and children's sleep duration at various ages. 74

Supplementary Figure S7. Observational associations between seven different instrumental variables and children's time outdoors at various ages. 75

Supplementary Figure S8. Observational associations between seven different instrumental variables and children's time reading at various ages. 76

Supplementary Figure S9. Instrumental variable analysis of the relationship between sleep duration and refractive error. 77

Supplementary Figure S10. Instrumental variable analysis of the relationship between time outdoors and refractive error. 78

Supplementary Figure S11. Instrumental variable analysis of the relationship between time reading and refractive error. 79

Supplementary Figure S12. Risk factors associated with the outcome ‘refractive error at age 15 years’ in the cross-sectional sample in analyses using the top three instrumental variables or all seven instrumental variables. 80

Supplementary Note 1. Code for deriving exposure variables.

```
library(haven)
library(plyr)
library(data.table)

rm(list=ls())

file_in="B3675.sav"
file_out="B3675_envars_2023-10-23.csv"

data1             <- as.data.frame(read_sav(file=file_in))

# Time outdoors
# -------------

# [AGE 38 months] Hours per Day Outside on a Weekday/Weekend day
# [1=None; 2=Less than one hour; 3=One to two hours; 4=Two or more hours]
# score as 1=0, 2=0.5, 3=1.5, 4=2****
# do NOT merge level 1 (n=8a, n=6b) with level 2, despite small n

data1$TO38a      <- as.factor(data1$kg271)
data1$TO38a      <- revalue(data1$TO38a, c("1"="0", "2"="0.5", "3"="1.5", "4"="2"))
data1$TO38a      <- as.numeric(as.character(data1$TO38a))
data1$TO38b      <- as.factor(data1$KG275)
data1$TO38b      <- revalue(data1$TO38b, c("1"="0", "2"="0.5", "3"="1.5", "4"="2"))
data1$TO38b      <- as.numeric(as.character(data1$TO38b))
data1$TO38       <- ((5*data1$TO38a)+(2*data1$TO38b))/7

# [AGE 54 months] Hours per Day Outside in Summer on Weekday/Weekend Day
# [AGE 54 months] Hours per Day Outside in Winter on Weekday/Weekend Day
# [1=None; 2=Less than one hour; 3=One to two hours; 4=Three or more hours]
# score as 1=0, 2=0.5, 3=1.5, 4=3
# Do NOT merge, as good chance to feed into final var
# weekdays = x 5 } 1 week  # Summer = 13 weeks (3 months), 6 weeks holidays (7 weeks Summer if holiday Q asked)
# weekends = x 2 }         # Winter = 13 weeks (3 months), 3 weeks holidays (10 weeks Winter if holiday Q asked)

data1$TO54a      <- as.factor(data1$kk325)
data1$TO54a      <- revalue(data1$TO54a, c("1"="0", "2"="0.5", "3"="1.5", "4"="3"))
data1$TO54a      <- as.numeric(as.character(data1$TO54a))
data1$TO54b      <- as.factor(data1$kk326)
data1$TO54b      <- revalue(data1$TO54b, c("1"="0", "2"="0.5", "3"="1.5", "4"="3"))
data1$TO54b      <- as.numeric(as.character(data1$TO54b))
```

```
data1$TO54c      <- as.factor(data1$kk328)
data1$TO54c      <- revalue(data1$TO54c, c("1"="0", "2"="0.5", "3"="1.5", "4"="3"))
data1$TO54c      <- as.numeric(as.character(data1$TO54c))
data1$TO54d      <- as.factor(data1$kk329)
data1$TO54d      <- revalue(data1$TO54d, c("1"="0", "2"="0.5", "3"="1.5", "4"="3"))
data1$TO54d      <- as.numeric(as.character(data1$TO54d))
data1$TO54summer <- rowMeans(data1[,c(rep("TO54a",5),rep("TO54b",2))],na.rm=FALSE)
data1$TO54winter <- rowMeans(data1[,c(rep("TO54c",5),rep("TO54d",2))],na.rm=FALSE)
data1$TO54       <- rowMeans(data1[,c("TO54summer","TO54winter")],na.rm=FALSE)

# [AGE 65 months] As above

data1$TO65a      <- as.factor(data1$km3061)
data1$TO65a      <- revalue(data1$TO65a, c("1"="0", "2"="0.5", "3"="1.5", "4"="3"))
data1$TO65a      <- as.numeric(as.character(data1$TO65a))
data1$TO65b      <- as.factor(data1$km3071)
data1$TO65b      <- revalue(data1$TO65b, c("1"="0", "2"="0.5", "3"="1.5", "4"="3"))
data1$TO65b      <- as.numeric(as.character(data1$TO65b))
data1$TO65c      <- as.factor(data1$km3062)
data1$TO65c      <- revalue(data1$TO65c, c("1"="0", "2"="0.5", "3"="1.5", "4"="3"))
data1$TO65c      <- as.numeric(as.character(data1$TO65c))
data1$TO65d      <- as.factor(data1$km3072)
data1$TO65d      <- revalue(data1$TO65d, c("1"="0", "2"="0.5", "3"="1.5", "4"="3"))
data1$TO65d      <- as.numeric(as.character(data1$TO65d))
data1$TO65summer <- rowMeans(data1[,c(rep("TO65a",5),rep("TO65b",2))],na.rm=FALSE)
data1$TO65winter <- rowMeans(data1[,c(rep("TO65c",5),rep("TO65d",2))],na.rm=FALSE)
data1$TO65       <- rowMeans(data1[,c("TO65summer","TO65winter")],na.rm=FALSE)

# [AGE 77 months] Time per Day Outside in Summer on Weekday/Weekend day/Holidays
# [AGE 77 months] Time per Day Outside in Winter on Weekday/Weekend day/Holidays
# [1=None; 2=Less than one hour; 3=One to two hours; 4=Three or more hours]
# score as 1=0, 2=0.5, 3=1.5, 4=3

data1$TO77a       <-  as.factor(data1$kp5021)
data1$TO77a       <-  revalue(data1$TO77a, c("1"="0", "2"="0.5", "3"="1.5", "4"="3"))
data1$TO77a       <-  as.numeric(as.character(data1$TO77a))
data1$TO77b       <-  as.factor(data1$kp5041)
data1$TO77b       <-  revalue(data1$TO77b, c("1"="0", "2"="0.5", "3"="1.5", "4"="3"))
data1$TO77b       <-  as.numeric(as.character(data1$TO77b))
data1$TO77c       <-  as.factor(data1$kp5022)
data1$TO77c       <-  revalue(data1$TO77c, c("1"="0", "2"="0.5", "3"="1.5", "4"="3"))
data1$TO77c       <-  as.numeric(as.character(data1$TO77c))
data1$TO77d       <-  as.factor(data1$kp5042)
data1$TO77d       <-  revalue(data1$TO77d, c("1"="0", "2"="0.5", "3"="1.5", "4"="3"))
```

```
data1$TO77d       <-  as.numeric(as.character(data1$TO77d))
data1$TO77sumhol  <-  as.factor(data1$kp5061) #sum hol
data1$TO77sumhol  <-  revalue(data1$TO77sumhol, c("1"="0", "2"="0.5", "3"="1.5", "4"="3"))
data1$TO77sumhol  <-  as.numeric(as.character(data1$TO77sumhol))
data1$TO77winhol  <-  as.factor(data1$kp5062) #win hol
data1$TO77winhol  <-  revalue(data1$TO77winhol, c("1"="0", "2"="0.5", "3"="1.5", "4"="3"))
data1$TO77winhol  <-  as.numeric(as.character(data1$TO77winhol))
data1$TO77summer  <-  rowMeans(data1[,c(rep("TO77a",5),rep("TO77b",2))],na.rm=FALSE)
data1$TO77winter  <-  rowMeans(data1[,c(rep("TO77c",5),rep("TO77d",2))],na.rm=FALSE)
z                 <-  data1[,c(rep("TO77summer",7), rep("TO77winter",10), rep("TO77sumhol",6), rep("TO77winhol",3))]
data1$TO77        <-  rowMeans(z,na.rm=FALSE)

# [AGE 103 months] As above

data1$TO103a      <-  as.factor(data1$kt1151)
data1$TO103a      <-  revalue(data1$TO103a, c("1"="0", "2"="0.5", "3"="1.5", "4"="3"))
data1$TO103a      <-  as.numeric(as.character(data1$TO103a))
data1$TO103b      <-  as.factor(data1$kt1171)
data1$TO103b      <-  revalue(data1$TO103b, c("1"="0", "2"="0.5", "3"="1.5", "4"="3"))
data1$TO103b      <-  as.numeric(as.character(data1$TO103b))
data1$TO103c      <-  as.factor(data1$kt1152)
data1$TO103c      <-  revalue(data1$TO103c, c("1"="0", "2"="0.5", "3"="1.5", "4"="3"))
data1$TO103c      <-  as.numeric(as.character(data1$TO103c))
data1$TO103d      <-  as.factor(data1$kt1172)
data1$TO103d      <-  revalue(data1$TO103d, c("1"="0", "2"="0.5", "3"="1.5", "4"="3"))
data1$TO103d      <-  as.numeric(as.character(data1$TO103d))
data1$TO103sumhol <-  as.factor(data1$kt1191) #sum hol
data1$TO103sumhol <-  revalue(data1$TO103sumhol, c("1"="0", "2"="0.5", "3"="1.5", "4"="3"))
data1$TO103sumhol <-  as.numeric(as.character(data1$TO103sumhol))
data1$TO103winhol <-  as.factor(data1$kt1192) #win hol
data1$TO103winhol <-  revalue(data1$TO103winhol, c("1"="0", "2"="0.5", "3"="1.5", "4"="3"))
data1$TO103winhol <-  as.numeric(as.character(data1$TO103winhol))
data1$TO103summer <-  rowMeans(data1[,c(rep("TO103a",5),rep("TO103b",2))],na.rm=FALSE)
data1$TO103winter <-  rowMeans(data1[,c(rep("TO103c",5),rep("TO103d",2))],na.rm=FALSE)
z                 <-  data1[,c(rep("TO103summer",7), rep("TO103winter",10), rep("TO103sumhol",6), rep("TO103winhol",3))]
data1$TO103       <-  rowMeans(z,na.rm=FALSE)


data1$TO103x      <-  as.factor(data1$kt1171)
data1$TO103x      <-  revalue(data1$TO103x, c("1"="0", "2"="0", "3"="0", "4"="1")) #  (4=2)  (3=1)  (2=1)  (ELSE=SYSMIS)  INTO
Outdoor1171 .
data1$Outdoor1171 <-  as.numeric(as.character(data1$TO103x))

data1$TO103y      <-  as.factor(data1$kt1151)
```

```
data1$TO103y      <-  revalue(data1$TO103y, c("1"="0", "2"="0", "3"="0", "4"="1")) #  IF (Missing()=0) Outdoor1151 = 1+(kt1151>3)
data1$Outdoor1151 <-  as.numeric(as.character(data1$TO103y))

# Time outdoors: YP self-reported at age 22 years ***Different to envar derivation previously ****
# Responses: 1="None", 2= "<1 hour", 3="1-2 hours", 4="3-4 hours", 5="5-6 hours", 6="7-8 hours", 7="9+ hours"
# xResponses: 0="None", 1= "<1 hour", 2="1-2 hours", 3="3-4 hours", 4="5-6 hours", 5="7-8 hours", 6="9+ hours"
# YPB2064     B7e: Number of hours per day spent outdoors in summer on an average weekday
# YPB2074     B8e: Number of hours per day spent outdoors in summer on an average weekend day
# YPB2065     B7f: Number of hours per day spent outdoors in winter on an average weekday     [NOT REQUESTED]
# YPB2075     B8f: Number of hours per day spent outdoors in winter on an average weekend day [NOT REQUESTED]

data1$TO22sumWK   <-  as.factor(data1$YPB2064)
data1$TO22sumWK   <-  revalue(data1$TO22sumWK, c("1"="0", "2"="0.5", "3"="1.5", "4"="3.5", "5"="5.5", "6"="7.5", "7"="9"))
data1$TO22sumWK   <-  as.numeric(as.character(data1$TO22sumWK))
data1$TO22sumWE   <-  as.factor(data1$YPB2074)
data1$TO22sumWE   <-  revalue(data1$TO22sumWE, c("1"="0", "2"="0.5", "3"="1.5", "4"="3.5", "5"="5.5", "6"="7.5", "7"="9"))
data1$TO22sumWE   <-  as.numeric(as.character(data1$TO22sumWE))

data1$TO22sum     <- rowMeans(data1[,c(rep("TO22sumWK",5),rep("TO22sumWE",2))],na.rm=FALSE)
data1$TO22yrSP    <- data1$TO22sum

# Time outdoors: YP self-reported at age 26 years
# Responses: 0="None", 1= "<1 hour", 2="1-2 hours", 3="3-4 hours", 4="5-6 hours", 5="7-8 hours", 6="9+ hours"
# YPF3120     c7e: Hours per day YP spends outdoors in summer on an average weekday
# YPF3130     c7f: Hours per day YP spends outdoors in winter on an average weekday
# YPF3190     c8e: Hours per day YP spends outdoors in summer on an average weekend day
# YPF3200     c8f: Hours per day YP spends outdoors in winter on an average weekend day

data1$TO26sumWK   <-  as.factor(data1$YPF3120)
data1$TO26sumWK   <-  revalue(data1$TO26sumWK, c("0"="0", "1"="0.5", "2"="1.5", "3"="3.5", "4"="5.5", "5"="7.5", "6"="9"))
data1$TO26sumWK   <-  as.numeric(as.character(data1$TO26sumWK))
data1$TO26winWK   <-  as.factor(data1$YPF3130)
data1$TO26winWK   <-  revalue(data1$TO26winWK, c("0"="0", "1"="0.5", "2"="1.5", "3"="3.5", "4"="5.5", "5"="7.5", "6"="9"))
data1$TO26winWK   <-  as.numeric(as.character(data1$TO26winWK))
data1$TO26sumWE   <-  as.factor(data1$YPF3190)
data1$TO26sumWE   <-  revalue(data1$TO26sumWE, c("0"="0", "1"="0.5", "2"="1.5", "3"="3.5", "4"="5.5", "5"="7.5", "6"="9"))
data1$TO26sumWE   <-  as.numeric(as.character(data1$TO26sumWE))
data1$TO26winWE   <-  as.factor(data1$YPF3200)
data1$TO26winWE   <-  revalue(data1$TO26winWE, c("0"="0", "1"="0.5", "2"="1.5", "3"="3.5", "4"="5.5", "5"="7.5", "6"="9"))
data1$TO26winWE   <-  as.numeric(as.character(data1$TO26winWE))

data1$TO26sum     <- rowMeans(data1[,c(rep("TO26sumWK",5),rep("TO26sumWE",2))],na.rm=FALSE)
data1$TO26win     <- rowMeans(data1[,c(rep("TO26winWK",5),rep("TO26winWE",2))],na.rm=FALSE)
data1$TO26yrSP    <- rowMeans(data1[,c("TO26sum","TO26win")],na.rm=FALSE)
```

```
# [AGE 167 months] Time per Day Outside in Summer on Weekday/Weekend day/Holidays
# [AGE 167 months] Time per Day Outside in Winter on Weekday/Weekend day/Holidays
# [1=None; 2=Less than one hour; 3=One to two hours; 4=Three or more hours]
# score as 1=0, 2=0.5, 3=1.5, 4=3
# ccq101     A1b1: Average time spent on a school weekday out of doors in the summer   **Child Completed**
# ccq102     A1c1: Average time spent on a school weekday out of doors in the winter   **Child Completed**
# ccq121     A1b2: Average time spent on a weekend day out of doors in the summer      **Child Completed**
# ccq122     A1c2: Average time spent on a weekend day out of doors in the winter    **Child Completed**
# ccq141     A1b3: Average time spent on normal days in school holidays out of doors in the summer
# ccq142     A1c3: Average time spent on normal days in school holidays out of doors in the winter
# ccq148     A1i3: Average time spent on normal days in school holidays reading books for pleasure

data1$TO167a       <-  as.factor(data1$ccq101)
data1$TO167a       <-  revalue(data1$TO167a, c("1"="0", "2"="0.5", "3"="1.5", "4"="3"))
data1$TO167a       <-  as.numeric(as.character(data1$TO167a))
data1$TO167b       <-  as.factor(data1$ccq121)
data1$TO167b       <-  revalue(data1$TO167b, c("1"="0", "2"="0.5", "3"="1.5", "4"="3"))
data1$TO167b       <-  as.numeric(as.character(data1$TO167b))
data1$TO167c       <-  as.factor(data1$ccq102)
data1$TO167c       <-  revalue(data1$TO167c, c("1"="0", "2"="0.5", "3"="1.5", "4"="3"))
data1$TO167c       <-  as.numeric(as.character(data1$TO167c))
data1$TO167d       <-  as.factor(data1$ccq122)
data1$TO167d       <-  revalue(data1$TO167d, c("1"="0", "2"="0.5", "3"="1.5", "4"="3"))
data1$TO167d       <-  as.numeric(as.character(data1$TO167d))

data1$TO167sumhol  <-  as.factor(data1$ccq141) #sum hol
data1$TO167sumhol  <-  revalue(data1$TO167sumhol, c("1"="0", "2"="0.5", "3"="1.5", "4"="3"))
data1$TO167sumhol  <-  as.numeric(as.character(data1$TO77sumhol))
data1$TO167winhol  <-  as.factor(data1$ccq142) #win hol
data1$TO167winhol  <-  revalue(data1$TO167winhol, c("1"="0", "2"="0.5", "3"="1.5", "4"="3"))
data1$TO167winhol  <-  as.numeric(as.character(data1$TO167winhol))

data1$TO167summer <- rowMeans(data1[,c(rep("TO167a",5),rep("TO167b",2))],na.rm=FALSE)
data1$TO167winter <- rowMeans(data1[,c(rep("TO167c",5),rep("TO167d",2))],na.rm=FALSE)
z                 <-  data1[,c(rep("TO167summer",7), rep("TO167winter",10), rep("TO167sumhol",6), rep("TO167winhol",3))]
data1$TO167SP     <-  rowMeans(z,na.rm=FALSE)

# [AGE 198 months] Time per Day Outside in Summer on Weekday/Weekend day/Holidays
# [AGE 198 months] Time per Day Outside in Winter on Weekday/Weekend day/Holidays
# [1=None; 2=Less than one hour; 3=One to two hours; 4=Three or more hours]
# score as 1=0, 2=0.5, 3=1.5, 4=3

# ccs1001    A1b1: Average time spent per day out of doors in summer on a typical weekday     **Child Completed**
```

```
# ccs1002    A1c1: Average time spent per day out of doors in winter on a typical weekday     **Child Completed**
# ccs1021    A1b2: Average time spent per day out of doors in summer on a typical weekend day **Child Completed**
# ccs1022    A1c2: Average time spent per day out of doors in winter on a typical weekend day **Child Completed**

data1$TO198a       <- as.factor(data1$ccs1001)
data1$TO198a       <- revalue(data1$TO198a, c("1"="0", "2"="0.5", "3"="1.5", "4"="3"))
data1$TO198a       <- as.numeric(as.character(data1$TO198a))
data1$TO198b       <- as.factor(data1$ccs1021)
data1$TO198b       <- revalue(data1$TO198b, c("1"="0", "2"="0.5", "3"="1.5", "4"="3"))
data1$TO198b       <- as.numeric(as.character(data1$TO198b))
data1$TO198c       <- as.factor(data1$ccs1002)
data1$TO198c       <- revalue(data1$TO198c, c("1"="0", "2"="0.5", "3"="1.5", "4"="3"))
data1$TO198c       <- as.numeric(as.character(data1$TO198c))
data1$TO198d       <- as.factor(data1$ccs1022)
data1$TO198d       <- revalue(data1$TO198d, c("1"="0", "2"="0.5", "3"="1.5", "4"="3"))
data1$TO198d       <- as.numeric(as.character(data1$TO198d))
data1$TO198summer  <- rowMeans(data1[,c(rep("TO198a",5),rep("TO198b",2))],na.rm=FALSE)
data1$TO198winter  <- rowMeans(data1[,c(rep("TO198c",5),rep("TO198d",2))],na.rm=FALSE)
data1$TO198SP      <- rowMeans(data1[,c("TO198summer","TO198winter")],na.rm=FALSE)

# Reading books
# -------------

# [AGE 54 months] Hours per Day Looking at Books on Weekday/Weekend Day
# [1=None; 2=Less than one hour; 3=One to two hours; 4=Three or more hours]
# score as 1=0, 2=0.5, 3=1.5, 4=3

data1$RB54a   <- as.factor(data1$kk343)
data1$RB54a   <- revalue(data1$RB54a, c("1"="0", "2"="0.5", "3"="1.5", "4"="3"))
data1$RB54a   <- as.numeric(as.character(data1$RB54a))
data1$RB54b   <- as.factor(data1$kk344)
data1$RB54b   <- revalue(data1$RB54b, c("1"="0", "2"="0.5", "3"="1.5", "4"="3"))
data1$RB54b   <- as.numeric(as.character(data1$RB54b))
data1$RB54    <- rowMeans(data1[,c(rep("RB54a",5),rep("RB54b",2))],na.rm=FALSE)

# [AGE 65 months] Hours per Day Looking at Books on Weekday/Weekend Day
# [1=None; 2=Less than one hour; 3=One to two hours; 4=Three or more hours]
# score as 1=0, 2=0.5, 3=1.5, 4=3

data1$RB65a   <- as.factor(data1$km3067)
data1$RB65a   <- revalue(data1$RB65a, c("1"="0", "2"="0.5", "3"="1.5", "4"="3"))
data1$RB65a   <- as.numeric(as.character(data1$RB65a))
data1$RB65b   <- as.factor(data1$km3077)
data1$RB65b   <- revalue(data1$RB65b, c("1"="0", "2"="0.5", "3"="1.5", "4"="3"))
```

```
data1$RB65b   <- as.numeric(as.character(data1$RB65b))
data1$RB65    <- rowMeans(data1[,c(rep("RB65a",5),rep("RB65b",2))],na.rm=FALSE)

# [AGE 77 months] Hours per Day Reading Books on Weekday/Weekend Day/Holidays
# [1=None; 2=Less than one hour; 3=One to two hours; 4=Three or more hours]
# score as 1=0, 2=0.5, 3=1.5, 4=3; 17 wks non-hols + 9 wks hols

data1$RB77a       <- as.factor(data1$kp5028)
data1$RB77a       <- revalue(data1$RB77a, c("1"="0", "2"="0.5", "3"="1.5", "4"="3"))
data1$RB77a       <- as.numeric(as.character(data1$RB77a))
data1$RB77b       <- as.factor(data1$kp5048)
data1$RB77b       <- revalue(data1$RB77b, c("1"="0", "2"="0.5", "3"="1.5", "4"="3"))
data1$RB77b       <- as.numeric(as.character(data1$RB77b))
data1$RB77nonhols <- rowMeans(data1[,c(rep("RB77a",5),rep("RB77b",2))],na.rm=FALSE)
data1$RB77hols    <- as.factor(data1$kp5068)
data1$RB77hols    <- revalue(data1$RB77hols, c("1"="0", "2"="0.5", "3"="1.5", "4"="3"))
data1$RB77hols    <- as.numeric(as.character(data1$RB77hols))
data1$RB77        <- rowMeans(data1[,c(rep("RB77nonhols",17),rep("RB77hols",9))],na.rm=FALSE)

# [AGE 103 months] Hours per Day Reading Books on Weekday/Weekend day/Holidays
# [1=None; 2=Less than one hour; 3=One to two hours; 4=Three or more hours]
# score as 1=0, 2=0.5, 3=1.5, 4=3

data1$RB103a       <- as.factor(data1$kt1158)
data1$RB103a       <- revalue(data1$RB103a, c("1"="0", "2"="0.5", "3"="1.5", "4"="3"))
data1$RB103a       <- as.numeric(as.character(data1$RB103a))
data1$RB103b       <- as.factor(data1$kt1178)
data1$RB103b       <- revalue(data1$RB103b, c("1"="0", "2"="0.5", "3"="1.5", "4"="3"))
data1$RB103b       <- as.numeric(as.character(data1$RB103b))
data1$RB103nonhols <- rowMeans(data1[,c("RB103a","RB103a","RB103a","RB103a","RB103a","RB103b","RB103b")],na.rm=FALSE)
data1$RB103hols    <- as.factor(data1$kt1198)
data1$RB103hols    <- revalue(data1$RB103hols, c("1"="0", "2"="0.5", "3"="1.5", "4"="3"))
data1$RB103hols    <- as.numeric(as.character(data1$RB103hols))
data1$RB103        <- rowMeans(data1[,c(rep("RB103nonhols",17),rep("RB103hols",9))],na.rm=FALSE)

data1$RB103x       <- as.factor(data1$kt1198)
data1$RB103x       <- revalue(data1$RB103x, c("1"="0", "2"="0", "3"="1", "4"="1")) #IF (Missing(kt1198)=0) ReadingBinary =
1+(kt1198>2) .
data1$ReadingBinary <- as.numeric(as.character(data1$RB103x))

# [AGE 167 months] Time per Day Outside in Summer on Weekday/Weekend day/Holidays
# [AGE 167 months] Time per Day Outside in Winter on Weekday/Weekend day/Holidays
# [1=None; 2=Less than one hour; 3=One to two hours; 4=Three or more hours]
# score as 1=0, 2=0.5, 3=1.5, 4=3
```

```
# ccq108     A1i1: Average time spent on a school weekday reading books for pleasure                **Child Completed**
# ccq128     A1i2: Average time spent on a weekend day reading books for pleasure                   **Child Completed**
# ccq148     A1i3: Average time spent on normal days in school holidays reading books for pleasure  **Child Completed**

data1$RB167a       <- as.factor(data1$ccq108)
data1$RB167a       <- revalue(data1$RB167a, c("1"="0", "2"="0.5", "3"="1.5", "4"="3"))
data1$RB167a       <- as.numeric(as.character(data1$RB167a))
data1$RB167b       <- as.factor(data1$ccq128)
data1$RB167b       <- revalue(data1$RB167b, c("1"="0", "2"="0.5", "3"="1.5", "4"="3"))
data1$RB167b       <- as.numeric(as.character(data1$RB167b))
data1$RB167nonhols <- rowMeans(data1[,c("RB167a","RB167a","RB167a","RB167a","RB167a","RB167b","RB167b")],na.rm=FALSE)
data1$RB167hols    <- as.factor(data1$ccq148)
data1$RB167hols    <- revalue(data1$RB167hols, c("1"="0", "2"="0.5", "3"="1.5", "4"="3"))
data1$RB167hols    <- as.numeric(as.character(data1$RB167hols))
data1$RB167SP      <- rowMeans(data1[,c(rep("RB167nonhols",17),rep("RB167hols",9))],na.rm=FALSE)

# [AGE 198 months] Time per Day Outside in Summer on Weekday/Weekend day/Holidays
# [AGE 198 months] Time per Day Outside in Winter on Weekday/Weekend day/Holidays
# [1=None; 2=Less than one hour; 3=One to two hours; 4=Three or more hours]
# score as 1=0, 2=0.5, 3=1.5, 4=3
# ccs1008    A1i1: Average time spent per day reading books for pleasure on a typical weekday      **Child Completed**
# ccs1028    A1i2: Average time spent per day reading books for pleasure on a typical weekend day  **Child Completed**

data1$RB198a       <- as.factor(data1$ccs1008)
data1$RB198a       <- revalue(data1$RB198a, c("1"="0", "2"="0.5", "3"="1.5", "4"="3"))
data1$RB198a       <- as.numeric(as.character(data1$RB198a))
data1$RB198b       <- as.factor(data1$ccs1028)
data1$RB198b       <- revalue(data1$RB198b, c("1"="0", "2"="0.5", "3"="1.5", "4"="3"))
data1$RB198b       <- as.numeric(as.character(data1$RB198b))
data1$RB198SP      <- rowMeans(data1[,c("RB198a","RB198a","RB198a","RB198a","RB198a","RB198b","RB198b")],na.rm=FALSE)

# Sleep
# -----

data1$sleeptime_schoolday_TF3   <- as.numeric(data1$fh5440) + (as.numeric(data1$fh5441)/60)
data1$sleeptime_weekend_TF3     <- as.numeric(data1$fh5480) + (as.numeric(data1$fh5481)/60)
data1$SLP186SP                  <-
rowMeans(data1[,c(rep("sleeptime_schoolday_TF3",5),rep("sleeptime_weekend_TF3",2))],na.rm=FALSE)

data1$bedtime_42                <- as.factor(data1$kj207)
data1$bedtime_42                <- revalue(data1$bedtime_42, c("30"  =NA,     "100" =NA,     "200" =NA,     "1530"="3.5",
"1600"="4",    "1630"="4.5",  "1700"="5",
                                                               "1730"="5.5",  "1745"="5.75", "1800"="6",    "1815"="6.25",
"1820"="6.33", "1830"="6.5",  "1845"="6.75",
```

```
                                                               "1850"="6.83", "1900"="7",    "1910"="7.17", "1915"="7.25",
"1920"="7.33", "1930"="7.5",  "1940"="7.66",
                                                               "1945"="7.75", "1950"="7.83", "2000"="8",    "2008"="8.16",
"2010"="8.16", "2015"="8.25", "2030"="8.5",
                                                               "2045"="8.75", "2100"="9",    "2115"="9.25", "2130"="9.5",
"2145"="9.75", "2200"="10",   "2215"="10.25",
                                                               "2230"="10.5", "2300"="11",   "2330"="11.5", "2400"="12"))
data1$bedtime_42                <- as.numeric(as.character(data1$bedtime_42))

data1$waketime_42               <- as.factor(data1$kj208)
data1$waketime_42               <- revalue(data1$waketime_42, c("0"  =NA,     "70" =NA,      "100"=NA,      "230"=NA,
"300"="3",     "400"="4",    "430"="4.5",
                                                                "500" ="5",    "515"="5.25",  "530"="5.5",   "537"="5.5",
"545"="5.75",  "600"="6",     "610"="6.17",
                                                                "615"="6.25",  "620"="6.33",  "630"="6.5",   "640"="6.66",
"644"="6.75",  "645"="6.75",  "650"="6.83",
                                                                "654"="6.9",   "655"="6.9",   "680"="7",     "700"="7",
"710"="7.13",  "715"="7.25",  "720"="7.33",
                                                                "725"="7.5",   "730"="7.5",   "740"="7.66",  "745"="7.75",
"750"="7.83",  "800"="8",     "810"="8.17",
                                                                "815"="8.25",  "830"="8.5",   "845"="8.75",  "850"="8.83",
"900"="9",     "930"="9.5",   "1000"="10",
                                                                "1030"="10.5", "1100"="11",   "1200"="12",   "1400"=NA))
data1$waketime_42               <- as.numeric(as.character(data1$waketime_42))
data1$SLP42                     <- (12 - data1$bedtime_42 ) + data1$waketime_42

data1$bedtime_57                <- as.numeric(ifelse(data1$kl222a==97, NA, data1$kl222a)) + (as.numeric(ifelse(data1$kl222b==97,
NA, data1$kl222b))/60)
data1$waketime_57               <- as.numeric(data1$kl223a) + (as.numeric(data1$kl223b)/60)
data1$SLP57                     <- (12 - data1$bedtime_57) + data1$waketime_57

data1$bedtime_69                <- as.numeric(data1$kn2011a) + (as.numeric(data1$kn2011b)/60)
data1$waketime_69               <- as.numeric(data1$kn2020a) + (as.numeric(data1$kn2020b)/60)
data1$SLP69                     <- (12 - data1$bedtime_69) + data1$waketime_69

data1$bedtime_weekday_81        <- as.numeric(data1$kq252) + (as.numeric(data1$kq253)/60)
data1$bedtime_weekend_81        <- as.numeric(data1$kq254) + (as.numeric(data1$kq255)/60)
data1$bedtime_81                <- rowMeans(data1[,c(rep("bedtime_weekday_81",5),rep("bedtime_weekend_81",2))],na.rm=FALSE)
data1$waketime_weekday_81       <- as.numeric(data1$kq256) + (as.numeric(data1$kq257)/60)
data1$waketime_weekend_81       <- as.numeric(data1$kq258) + (as.numeric(data1$kq259)/60)
data1$waketime_81               <- rowMeans(data1[,c(rep("waketime_weekday_81",5),rep("waketime_weekend_81",2))],na.rm=FALSE)
data1$SLP81                     <- (12 - data1$bedtime_81) + data1$waketime_81
#data1$sleeptime_schoolday_81    <- as.numeric(data1$kq260)
#data1$sleeptime_weekend_81      <- as.numeric(data1$kq261)
```

```
#data1$SLP81                        <-
rowMeans(data1[,c(rep("sleeptime_schoolday_81",5),rep("sleeptime_weekend_81",2))],na.rm=FALSE)

data1$bedtime_weekday_115       <- as.numeric(ifelse(data1$ku341a>12,NA,data1$ku341a)) + (as.numeric(data1$ku341b)/60)
data1$bedtime_weekend_115       <- as.numeric(ifelse(data1$ku343a>12,NA,data1$ku343a)) + (as.numeric(data1$ku343b)/60)
data1$bedtime_115               <- rowMeans(data1[,c(rep("bedtime_weekday_115",5),rep("bedtime_weekend_115",2))],na.rm=FALSE)
data1$waketime_weekday_115      <- as.numeric(data1$ku340a) + (as.numeric(data1$ku340b)/60)
data1$waketime_weekend_115      <- as.numeric(data1$ku342a) + (as.numeric(data1$ku342b)/60)
data1$waketime_115              <- rowMeans(data1[,c(rep("waketime_weekday_115",5),rep("waketime_weekend_115",2))],na.rm=FALSE)
data1$SLP115                    <- (12 - data1$bedtime_115) + data1$waketime_115

data1$bedtime_weekday_140       <- as.numeric(ifelse(data1$kw4061a>12,NA,data1$kw4061a)) + (as.numeric(data1$kw4061b)/60)
data1$bedtime_weekend_140       <- as.numeric(ifelse(data1$kw4063a>12,NA,data1$kw4063a)) + (as.numeric(data1$kw4063b)/60)
data1$bedtime_140               <- rowMeans(data1[,c(rep("bedtime_weekday_140",5),rep("bedtime_weekend_140",2))],na.rm=FALSE)
data1$waketime_weekday_140      <- as.numeric(data1$kw4060a) + (as.numeric(data1$kw4060b)/60)
data1$waketime_weekend_140      <- as.numeric(data1$kw4062a) + (as.numeric(data1$kw4062b)/60)
data1$waketime_140              <- rowMeans(data1[,c(rep("waketime_weekday_140",5),rep("waketime_weekend_140",2))],na.rm=FALSE)
data1$SLP140                    <- (12 - data1$bedtime_140) + data1$waketime_140

data1$bedtime_var42_81          <-
apply(data1[,c("bedtime_42","bedtime_57","bedtime_69","bedtime_weekday_81","bedtime_weekend_81")],1, FUN=sd, na.rm=TRUE)


# Birth order
data1$oldersibs                 <- as.numeric(data1$kd432) + as.numeric(data1$kd433)
data1$youngersibs               <- as.numeric(data1$kd451) + as.numeric(data1$kd452)
data1$BirthOrder                <- (1 + data1$oldersibs)
data1$BirthOrder                <- ifelse(data1$BirthOrder > 4, 4, data1$BirthOrder)
data1$NumSiblings               <- (data1$oldersibs + data1$youngersibs)
data1$NumSiblings               <- ifelse(data1$NumSiblings > 3, 3, data1$NumSiblings)
data1$Parity                    <- (1 + as.numeric(data1$b032))
data1$Parity                    <- ifelse(data1$Parity > 4, 4, data1$Parity)

# Pet ownership
data1$CatOwner85                <- as.numeric(ifelse(data1$m3391==0,0,1))
data1$DogOwner85                <- as.numeric(ifelse(data1$m3392==0,0,1))
data1$CatOwner122               <- as.numeric(ifelse(data1$q3111==0,0,1))
data1$DogOwner122               <- as.numeric(ifelse(data1$q3112==0,0,1))
data1$CatOwner                  <- rowSums(data1[,c("CatOwner85","CatOwner122")], na.rm=TRUE)
data1$CatOwner                  <- ifelse(data1$CatOwner > 0, 1, 0)
data1$CatOwner                  <- ifelse(is.na(data1$CatOwner85) & is.na(data1$CatOwner122), NA, data1$CatOwner)
data1$DogOwner                  <- rowSums(data1[,c("DogOwner85","DogOwner122")], na.rm=TRUE)
data1$DogOwner                  <- ifelse(data1$DogOwner > 0, 1, 0)
data1$DogOwner                  <- ifelse(is.na(data1$DogOwner85) & is.na(data1$DogOwner122), NA, data1$DogOwner)
```

```
data1$CatOwnerSR                <- rowSums(data1[,c("cct1501","cct1511","cct1521","cct1531")], na.rm=TRUE)
data1$CatOwnerSR                <- ifelse(data1$CatOwnerSR > 0, 1, 0)
data1$CatOwnerSR                <- ifelse(is.na(data1$cct1501) & is.na(data1$cct1511) & is.na(data1$cct1521) &
is.na(data1$cct1531), NA, data1$CatOwnerSR)
data1$DogOwnerSR                <- rowSums(data1[,c("cct1502","cct1512","cct1522","cct1532")], na.rm=TRUE)
data1$DogOwnerSR                <- ifelse(data1$DogOwnerSR > 0, 1, 0)
data1$DogOwnerSR                <- ifelse(is.na(data1$cct1502) & is.na(data1$cct1512) & is.na(data1$cct1522) &
is.na(data1$cct1532), NA, data1$DogOwnerSR)

# TDI
data1$TDIQ                      <- as.numeric(data1$jan1997Townsendq5_YP) # quintile of TDI when child age 5 years

# Maternal age
data1$mat_age                   <- data1$mz028b

data2             <- data1[,c("cidB3675","qlet","Outdoor1151","Outdoor1171","ReadingBinary",
                             "TO38","TO54","TO65","TO77","TO103","TO167SP","TO198SP","TO22yrSP","TO26yrSP",
                             "RB54","RB65","RB77","RB103","RB167SP","RB198SP",
                             "SLP42","SLP57","SLP69","SLP81","SLP115","SLP140","SLP186SP","bedtime_var42_81",
                             "BirthOrder","CatOwner","CatOwnerSR","DogOwner","DogOwnerSR",
                             "mat_age","TDIQ")]

fwrite(data2,file=file_out,row.names=FALSE, na = "NA", quote=FALSE)
```

Supplementary Note 2. Code for fitting models.

```
# ALSPAC Sleep 2024-05-05
# ------------------------

library(data.table)
library(stringr)
library(ggplot2)
library(cowplot)
library(ggtext) # markdown text
library(lares) # right(); string function
library(fixest) # Fixest package provides the F-stat from the 1st stage; otherwise, the coefficients are the same as with ivreg
library(forester)
library(WVPlots) # PairPlot
library(plm)

rm(list=ls())

mydir=[path to directory]

###########################################################################################

file_yp              <- paste0(mydir,"alspac_sleep_2023-10-23.csv")
dataYP               <- as.data.frame(fread(file=file_yp, header=TRUE))
dataYP$age7          <- ifelse(dataYP$age7 < 0, NA, dataYP$age7)
dataYPL              <- melt(as.data.table(dataYP[which(dataYP$visits >= 3),]),
id=c("cidB3675","Sex","TDIQ","mat_age","PC1","PC2","PC3","visits","TO103","RB103","SLP115",

"bedtime_var42_81","DogOwner","CatOwner","BirthOrder","scoreTOSpgs","scoreEDUpgs","scoreSLPpgs"),
                                measure=patterns("^Yr", "^age"),value.name = c("avMSE", "Age"), variable.name = "Visit",
value.factor=TRUE)
dataYPL              <- as.data.frame(dataYPL)
dataYPL$Visit        <- as.numeric(dataYPL$Visit)
dataYPL1             <- dataYPL[order(dataYPL$cidB3675),]
dataYPL              <- dataYPL1[complete.cases(dataYPL1),]

dataYPW1             <- dataYP[which(dataYP$visits >= 1),]
dataYPW              <-
dataYPW1[complete.cases(dataYPW1[,c("Yr15_avMSE","Sex","TDIQ","mat_age","PC1","PC2","PC3","TO103","RB103","SLP115",

"bedtime_var42_81","DogOwner","CatOwner","BirthOrder","scoreTOSpgs","scoreEDUpgs","scoreSLPpgs")]),]
length(unique(dataYPL$cidB3675))
```

```
nrow(dataYPW)

# Participant demographics
# ------------------------

num_perc                <- function(trait,value){
trait                   <- as.character(trait)
value                   <- as.numeric(value)
n11                     <- nrow(dataYP[which((dataYP[,trait]==value)),])
n12                     <- nrow(dataYP[which(!is.na(dataYP[,trait])),])
n13                     <- sprintf("%.1f",(n11*100/n12))
n21                     <- nrow(dataYPW[which((dataYPW[,trait]==value)),])
n22                     <- nrow(dataYPW[which(!is.na(dataYPW[,trait])),])
n23                     <- sprintf("%.1f",(n21*100/n22))
n31                     <- nrow(dataYPLU[which((dataYPLU[,trait]==value)),])
n32                     <- nrow(dataYPLU[which(!is.na(dataYPLU[,trait])),])
n33                     <- sprintf("%.1f",(n31*100/n32))
c1                      <- paste0(n11," (",n13,"%)")
c2                      <- paste0(n21," (",n23,"%)")
c3                      <- paste0(n31," (",n33,"%)")
p12                     <- sprintf("%.3f", prop.test(x = c(n11, n21), n = c(n12, n22),alternative = "two.sided", correct =
TRUE)$p.value)
p13                     <- sprintf("%.3f", prop.test(x = c(n11, n31), n = c(n12, n32),alternative = "two.sided", correct =
TRUE)$p.value)
p12                     <- ifelse(p12=="0.000", "<0.001",p12)
p13                     <- ifelse(p13=="0.000", "<0.001",p13)
c4                      <- c(c1,c2,p12,c3,p13)
return(c4)}

mean_sd                 <- function(trait){
trait                   <- as.character(trait)
x1                      <- sprintf("%.2f",mean(dataYP[,trait],na.rm=TRUE))
sd1                     <- sprintf("%.2f",sd(dataYP[,trait],na.rm=TRUE))
x2                      <- sprintf("%.2f",mean(dataYPW[,trait]))
sd2                     <- sprintf("%.2f",sd(dataYPW[,trait]))
x3                      <- sprintf("%.2f",mean(dataYPLU[,trait]))
sd3                     <- sprintf("%.2f",sd(dataYPLU[,trait]))
c1                      <- paste0(x1," (",sd1,")")
c2                      <- paste0(x2," (",sd2,")")
c3                      <- paste0(x3," (",sd3,")")
p12                     <- sprintf("%.3f", t.test(x=dataYP[,trait], y=dataYPW[,trait], alternative = "two.sided", paired =
FALSE, var.equal = FALSE, conf.level = 0.95)$p.value)
p13                     <- sprintf("%.3f", t.test(x=dataYP[,trait], y=dataYPLU[,trait], alternative = "two.sided", paired =
FALSE, var.equal = FALSE, conf.level = 0.95)$p.value)
```

```
p12                        <- ifelse(p12=="0.000", "<0.001",p12)
p13                        <- ifelse(p13=="0.000", "<0.001",p13)
c4                         <- c(c1,c2,p12,c3,p13)
return(c4)}

median_2575                <- function(trait){
trait                      <- as.character(trait)
x1                         <- sprintf("%.2f",median(dataYP[,trait],na.rm=TRUE))
x125                       <- sprintf("%.2f",summary(dataYP[,trait],na.rm=TRUE)[2])
x175                       <- sprintf("%.2f",summary(dataYP[,trait],na.rm=TRUE)[5])
x2                         <- sprintf("%.2f",median(dataYPW[,trait]))
x225                       <- sprintf("%.2f",summary(dataYPW[,trait])[2])
x275                       <- sprintf("%.2f",summary(dataYPW[,trait])[5])
x3                         <- sprintf("%.2f",median(dataYPLU[,trait]))
x325                       <- sprintf("%.2f",summary(dataYPLU[,trait])[2])
x375                       <- sprintf("%.2f",summary(dataYPLU[,trait])[5])
c1                         <- paste0(x1," (",x125,", ",x175,")")
c2                         <- paste0(x2," (",x225,", ",x275,")")
c3                         <- paste0(x3," (",x325,", ",x375,")")
p12                        <- sprintf("%.3f", wilcox.test(x=dataYP[,trait], y=dataYPW[,trait], alternative = "two.sided", paired =
FALSE, conf.level = 0.95)$p.value)
p13                        <- sprintf("%.3f", wilcox.test(x=dataYP[,trait], y=dataYPLU[,trait], alternative = "two.sided", paired =
FALSE, conf.level = 0.95)$p.value)
p12                        <- ifelse(p12=="0.000", "<0.001",p12)
p13                        <- ifelse(p13=="0.000", "<0.001",p13)
c4                         <- c(c1,c2,p12,c3,p13)
return(c4)}

age_sd                     <- function(value){
myage                      <- c(7,10,11,12,15)
value                      <- as.numeric(value)
trait                      <- paste0("age",myage[value])
x1                         <- sprintf("%.2f",mean(dataYP[,trait],na.rm=TRUE))
sd1                        <- sprintf("%.2f",sd(dataYP[,trait],na.rm=TRUE))
x2                         <- sprintf("%.2f",mean(dataYPW$age15))
sd2                        <- sprintf("%.2f",sd(dataYPW$age15))
x3                         <- sprintf("%.2f",mean(dataYPL[which(dataYPL$Visit==value),]$Age))
sd3                        <- sprintf("%.2f",sd(dataYPL[which(dataYPL$Visit==value),]$Age))
c1                         <- paste0(x1," (",sd1,")")
c2                         <- paste0(x2," (",sd2,")")
c3                         <- paste0(x3," (",sd3,")")
p12                        <- sprintf("%.3f", t.test(x=dataYP[,trait], y=dataYPW$age15, alternative = "two.sided", paired = FALSE,
var.equal = FALSE, conf.level = 0.95)$p.value)
```

```
p13                      <- sprintf("%.3f", t.test(x=dataYP[,trait], y=dataYPL[which(dataYPL$Visit==value),]$Age, alternative =
"two.sided", paired = FALSE, var.equal = FALSE, conf.level = 0.95)$p.value)
p12                      <- ifelse(p12=="0.000", "<0.001",p12)
p13                      <- ifelse(p13=="0.000", "<0.001",p13)
c2                       <- ifelse(value==5,c2,"-")
p12                      <- ifelse(value==5,p12,"-")
c4                       <- c(c1,c2,p12,c3,p13)
return(c4)}

ref_2575                 <- function(value){
myage                    <- c(7,10,11,12,15)
value                    <- as.numeric(value)
trait                    <- paste0("Yr",myage[value],"_avMSE")
x1                       <- sprintf("%.2f",median(dataYP[,trait],na.rm=TRUE))
x125                     <- sprintf("%.2f",summary(dataYP[,trait],na.rm=TRUE)[2])
x175                     <- sprintf("%.2f",summary(dataYP[,trait],na.rm=TRUE)[5])
x2                       <- sprintf("%.2f",median(dataYPW$Yr15_avMSE))
x225                     <- sprintf("%.2f",summary(dataYPW$Yr15_avMSE)[2])
x275                     <- sprintf("%.2f",summary(dataYPW$Yr15_avMSE)[5])
x3                       <- sprintf("%.2f",median(dataYPL[which(dataYPL$Visit==value),]$avMSE))
x325                     <- sprintf("%.2f",summary(dataYPL[which(dataYPL$Visit==value),]$avMSE)[2])
x375                     <- sprintf("%.2f",summary(dataYPL[which(dataYPL$Visit==value),]$avMSE)[5])
c1                       <- paste0(x1," (",x125,", ",x175,")")
c2                       <- paste0(x2," (",x225,", ",x275,")")
c3                       <- paste0(x3," (",x325,", ",x375,")")
p12                      <- sprintf("%.3f", wilcox.test(x=dataYP[,trait], y=dataYPW$Yr15_avMSE, alternative = "two.sided", paired
= FALSE, conf.level = 0.95)$p.value)
p13                      <- sprintf("%.3f", wilcox.test(x=dataYP[,trait], y=dataYPL[which(dataYPL$Visit==value),]$avMSE,
alternative = "two.sided", paired = FALSE, conf.level = 0.95)$p.value)
p12                      <- ifelse(p12=="0.000", "<0.001",p12)
p13                      <- ifelse(p13=="0.000", "<0.001",p13)
c2                       <- ifelse(value==5,c2,"-")
p12                      <- ifelse(value==5,p12,"-")
c4                       <- c(c1,c2,p12,c3,p13)
return(c4)}

dataYPLU                 <- dataYPL[which(!duplicated(dataYPL$cidB3675)),]

demotab                  <- as.data.frame(matrix(ncol=6,nrow=25))
names(demotab)           <- c("Trait", "Full sample","Cross-sectional sample","P-value","Longitudinal sample","P-value")
demotab$Trait            <- c("Sample size",
                              "Female (%)",
                              "Number of visits [median (25th,75th)]",
                              "Townsend deprivation index quintile [median (25th,75th)]",
```

```
                              "Maternal age [median (25th,75th)] (years)",
                              "Birth order [median (25th,75th)]",
                              "Sleep duration at age 9.5 years [median (25th,75th)] (hours/day)",
                              "Time outdoors at age 8.5 years [median (25th,75th)] (hours/day)",
                              "Time reading at age 8.5 years [median (25th,75th)] (hours/day)",
                              "Bedtime variability [median (25th,75th)] (SD units)",
                              "Dog ownership by family (%)",
                              "Cat ownership by family (%)",
                              "PGS for sleep duration [mean (SD)] (SD units)",
                              "PGS for time outdoors [mean (SD)](SD units)",
                              "PGS for EduYears [mean (SD)] (SD units)",
                              "Age at 7-year clinic [mean (SD)] (years)",
                              "Age at 10-year clinic [mean (SD)] (years)",
                              "Age at 11-year clinic [mean (SD)] (years)",
                              "Age at 12-year clinic [mean (SD)] (years)",
                              "Age at 15-year clinic [mean (SD)] (years)",
                              "Refractive error at 7-year clinic [median (25th,75th)] (D)",
                              "Refractive error at 10-year clinic [median (25th,75th)] (D)",
                              "Refractive error at 11-year clinic [median (25th,75th)] (D)",
                              "Refractive error at 12-year clinic [median (25th,75th)] (D)",
                              "Refractive error at 15-year clinic [median (25th,75th)] (D)")
demotab[,2:6]             <- "-"
demotab[1,2]              <- nrow(dataYP)
demotab[1,3]              <- nrow(dataYPW)
demotab[1,5]              <- length(unique(dataYPL$cidB3675))
demotab[2,2:6]            <- num_perc("Sex",2)
demotab[3,2:6]            <- median_2575("visits")
demotab[4,2:6]            <- median_2575("TDIQ")
demotab[5,2:6]            <- median_2575("mat_age")
demotab[6,2:6]            <- median_2575("BirthOrder")
demotab[7,2:6]            <- median_2575("SLP115")
demotab[8,2:6]            <- median_2575("TO103")
demotab[9,2:6]            <- median_2575("RB103")
demotab[10,2:6]            <- median_2575("bedtime_var42_81")
demotab[11,2:6]           <- num_perc("DogOwner",1)
demotab[12,2:6]           <- num_perc("CatOwner",1)
demotab[13,2:6]           <- mean_sd("scoreSLPpgs")
demotab[14,2:6]           <- mean_sd("scoreTOSpgs")
demotab[15,2:6]           <- mean_sd("scoreEDUpgs")
demotab[16,2:6]           <- age_sd(1)
demotab[17,2:6]           <- age_sd(2)
demotab[18,2:6]           <- age_sd(3)
demotab[19,2:6]           <- age_sd(4)
demotab[20,2:6]           <- age_sd(5)
```

```
demotab[21,2:6]          <- ref_2575(1)
demotab[22,2:6]          <- ref_2575(2)
demotab[23,2:6]          <- ref_2575(3)
demotab[24,2:6]          <- ref_2575(4)
demotab[25,2:6]          <- ref_2575(5)

demotab
outfile                  <- paste0(mydir,"Table1_2024-05-05.csv")
write.csv(demotab, file=outfile, row.names=FALSE)

median(dataYP$SLP115, na.rm=TRUE)
range(dataYP$TO103, na.rm=TRUE)
range(dataYP$RB103, na.rm=TRUE)

###############################################################################################

# Correlation in behaviours
# -------------------------

theme_qq <- function (base_size = 12, base_family = "") {
    theme_gray(base_size = base_size, base_family = base_family) %+replace%
        theme(
            axis.text = element_text(colour = "black"),
            axis.title.x = element_text(colour = "black", size=rel(0.9), margin = margin(t = 10)),
            axis.title.y = element_text(colour = "black", size=rel(0.9), angle=90, margin = margin(r = 10)),
            axis.text.x = element_text(colour = "black", size=rel(0.8)),
            axis.text.y = element_text(colour = "black", size=rel(0.8), margin = margin(r = 5)),
            plot.title = element_text(hjust = 0.5, size=rel(1), margin=margin(b = 5)),
            panel.grid.minor = element_blank(),
            panel.grid.major = element_blank(),
            plot.background = element_blank(),
            panel.background = element_rect(fill="white"),
            legend.text = element_text(size=rel(1)),
            legend.title = element_text(size=rel(1), hjust=0),
            legend.key.height = unit(0.4, 'cm'),
            legend.key = element_rect(fill = "transparent", colour = "transparent"),
            plot.margin = margin(0.4, 0.4, 0.4, 0.4, "cm"),
    )
}
theme_set(theme_qq())

PairPlotJG <- function(d, meas_vars,  group_var) {
  controlTable           <- data.frame(expand.grid(meas_vars, meas_vars, stringsAsFactors = FALSE))
  colnames(controlTable) <- c("x", "y")
```

```
  controlTable           <- cbind(data.frame(pair_key = paste(controlTable[[1]], controlTable[[2]]), stringsAsFactors = FALSE),
controlTable)
  d_aug                  <- cdata::rowrecs_to_blocks(d, controlTable, columnsToCopy = group_var)
  splt                   <- strsplit(d_aug$pair_key, split = " ", fixed = TRUE)
  d_aug$xv               <- vapply(splt, function(si) si[[1]], character(1))
  d_aug$yv               <- vapply(splt, function(si) si[[2]], character(1))
  d_aug$xv               <- factor(as.character(d_aug$xv), meas_vars)
  d_aug$yv               <- factor(as.character(d_aug$yv), meas_vars)
  return(d_aug)
}

# Correlation in sleep duration over childhood
# ---------------------------------------------

exposure_list            <- c("SLP42","SLP57","SLP69","SLP81","SLP115","SLP140")
exposure_full            <- c("Age 3.5 years","Age 5.0 years","Age 6.0 years","Age 7.0 years","Age 9.5 years","Age 11.5 years")
dataP                    <- dataYP[which(!is.na(dataYP$Sex)),c("Sex",exposure_list)]
dataP$Sex                <- as.factor(dataP$Sex)
d_aug                    <- PairPlotJG(dataP, exposure_list, "Sex")
d2                       <- d_aug
levels(d2$xv)            <- list("Age 3.5 years"="SLP42", "Age 5.0 years"="SLP57",  "Age 6.0 years"="SLP69",
                                 "Age 7.0 years"="SLP81", "Age 9.5 years"="SLP115", "Age 11.5 years"="SLP140")
levels(d2$yv)            <- list("Age 3.5 years"="SLP42", "Age 5.0 years"="SLP57",  "Age 6.0 years"="SLP69",
                                 "Age 7.0 years"="SLP81", "Age 9.5 years"="SLP115", "Age 11.5 years"="SLP140", "Age 15.5
years"="SLP186SP")
d2$xv                    <- ordered(d2$xv, levels=c("Age 3.5 years", "Age 5.0 years", "Age 6.0 years", "Age 7.0 years", "Age 9.5
years", "Age 11.5 years"))
d2$yv                    <- ordered(d2$yv, levels=c("Age 3.5 years", "Age 5.0 years", "Age 6.0 years", "Age 7.0 years", "Age 9.5
years", "Age 11.5 years"))

rmat            <- as.data.frame(matrix(nrow=(length(exposure_full)^2), ncol=3))
pmat            <- as.data.frame(matrix(nrow=(length(exposure_full)^2), ncol=3))
myrow           <- 1
for (i in 1:(length(exposure_full))){
for (j in 1:(length(exposure_full))){
 ct                 <- cor.test(x=dataYP[,exposure_list[i]], y=dataYP[,exposure_list[j]], method = "spearman", use =
"pairwise.complete.obs")
 rmat[myrow,1]      <- exposure_full[i]
 rmat[myrow,2]      <- exposure_full[j]
 rmat[myrow,3]      <- ct$estimate
 pmat[myrow,1]      <- exposure_full[i]
 pmat[myrow,2]      <- exposure_full[j]
 pmat[myrow,3]      <- ct$p.value
 myrow              <- myrow + 1
```

```
}}
names(rmat)          <- c("xv","yv","cm")
rmat$cm              <- sprintf("%.2f",as.numeric(rmat$cm))
rmat$mylabel         <- paste(rmat$cm,"<br>(*P* < 0.001)")
rmat$xv              <- ordered(rmat$xv, levels=c("Age 3.5 years", "Age 5.0 years", "Age 6.0 years", "Age 7.0 years", "Age 9.5 
years", "Age 11.5 years"))
rmat$yv              <- ordered(rmat$yv, levels=c("Age 3.5 years", "Age 5.0 years", "Age 6.0 years", "Age 7.0 years", "Age 9.5 
years", "Age 11.5 years"))

rmat[which(rmat$xv==exposure_full[length(exposure_full)] & rmat$yv==exposure_full[length(exposure_full)]),]$mylabel <- NA
d2[which(d2$xv==exposure_full[length(exposure_full)] & d2$yv==exposure_full[length(exposure_full)]),]               <- NA
for (i in 1:(length(exposure_full))){
for (j in 1:(length(exposure_full))){
 if(j <= i){ rmat[which(rmat$xv==exposure_full[i] & rmat$yv==exposure_full[j]),]$mylabel <- NA
 } else {       d2[which(d2$xv==exposure_full[i] & d2$yv==exposure_full[j]),]              <- NA
 }
}}

plot1                    <- ggplot(data = d2[which(!is.na(d2$xv)),], aes(x=x, y=y))+
                              theme(legend.position=c(0.91,0.09))+
                              theme(legend.title = element_blank())+
                              theme(legend.key.width = unit(0.2, 'cm'))+
                              geom_richtext(data = rmat, aes(x = 11, y = 11, label = mylabel),hjust = 0.5, vjust   = 0.5, fill = 
NA, label.colour = NA)+
                              scale_colour_manual(name=NULL, labels=c("Male", "Female"), values=c("dark blue","orange"))+
                              geom_point(alpha=0.3, aes(!!!simulate_aes_string(color="Sex")))+
                              scale_x_continuous(limits=c(7,15), breaks=c(8,10,12,14))+
                              scale_y_continuous(limits=c(7,15), breaks=c(8,10,12,14))+
                              theme(strip.background.x = element_blank())+
                              theme(strip.background.y = element_blank())+
                              theme(strip.text.x = element_text(face="bold", size=rel(1.1)))+
                              theme(strip.text.y = element_text(face="bold", size=rel(1.1)))+
                              labs(x="Sleep duration (hours)",y="Sleep duration (hours)")+
                              facet_grid(yv~xv)
#plot1
outfile   <- paste0(mydir,"FigureS1_2024-05-05.tiff")
tiff(outfile, width = 22, height = 22, units = "cm", compression = "lzw", res=300)
plot_grid(plot1, ncol=1)
dev.off()


# Correlation in time outdoors over childhood
# ---------------------------------------------
```

```
exposure_list            <- c("TO38","TO54","TO65","TO77","TO103","TO167SP")
exposure_full            <- c("Age 3.0 years","Age 4.5 years","Age 5.5 years","Age 6.5 years","Age 8.5 years","Age 14.0 years")
dataP                    <- dataYP[which(!is.na(dataYP$Sex)),c("Sex",exposure_list)]
dataP$Sex                <- as.factor(dataP$Sex)
d_aug                    <- PairPlotJG(dataP, exposure_list, "Sex")
d2                       <- d_aug
levels(d2$xv)            <- list("Age 3.0 years"="TO38", "Age 4.5 years"="TO54",  "Age 5.5 years"="TO65",  "Age 6.5
years"="TO77", "Age 8.5 years"="TO103",  "Age 14.0 years"="TO167SP")
levels(d2$yv)            <- list("Age 3.0 years"="TO38", "Age 4.5 years"="TO54",  "Age 5.5 years"="TO65",  "Age 6.5
years"="TO77", "Age 8.5 years"="TO103",  "Age 14.0 years"="TO167SP")
d2$xv                    <- ordered(d2$xv, levels=c("Age 3.0 years","Age 4.5 years","Age 5.5 years","Age 6.5 years","Age 8.5
years","Age 14.0 years"))
d2$yv                    <- ordered(d2$yv, levels=c("Age 3.0 years","Age 4.5 years","Age 5.5 years","Age 6.5 years","Age 8.5
years","Age 14.0 years"))

rmat            <- as.data.frame(matrix(nrow=(length(exposure_full)^2), ncol=3))
pmat            <- as.data.frame(matrix(nrow=(length(exposure_full)^2), ncol=3))
myrow           <- 1
for (i in 1:(length(exposure_full))){
for (j in 1:(length(exposure_full))){
 ct                <- cor.test(x=dataYP[,exposure_list[i]], y=dataYP[,exposure_list[j]], method = "spearman", use =
"pairwise.complete.obs")
 rmat[myrow,1]     <- exposure_full[i]
 rmat[myrow,2]     <- exposure_full[j]
 rmat[myrow,3]     <- ct$estimate
 pmat[myrow,1]     <- exposure_full[i]
 pmat[myrow,2]     <- exposure_full[j]
 pmat[myrow,3]     <- ct$p.value
 myrow             <- myrow + 1
}}
names(rmat)        <- c("xv","yv","cm")
rmat$cm            <- sprintf("%.2f",as.numeric(rmat$cm))
rmat$mylabel       <- paste(rmat$cm,"<br>(*P* < 0.001)")
rmat$xv            <- ordered(rmat$xv, levels=c("Age 3.0 years","Age 4.5 years","Age 5.5 years","Age 6.5 years","Age 8.5
years","Age 14.0 years"))
rmat$yv            <- ordered(rmat$yv, levels=c("Age 3.0 years","Age 4.5 years","Age 5.5 years","Age 6.5 years","Age 8.5
years","Age 14.0 years"))

rmat[which(rmat$xv==exposure_full[length(exposure_full)] & rmat$yv==exposure_full[length(exposure_full)]),]$mylabel <- NA
d2[which(d2$xv==exposure_full[length(exposure_full)] & d2$yv==exposure_full[length(exposure_full)]),]              <- NA
for (i in 1:(length(exposure_full))){
for (j in 1:(length(exposure_full))){
 if(j <= i){ rmat[which(rmat$xv==exposure_full[i] & rmat$yv==exposure_full[j]),]$mylabel <- NA
 } else {      d2[which(d2$xv==exposure_full[i] & d2$yv==exposure_full[j]),]              <- NA
```

```
 }
}}

plot2                    <- ggplot(data = d2[which(!is.na(d2$xv)),], aes(x=x, y=y))+
                            theme(legend.position=c(0.91,0.09))+
                            theme(legend.title = element_blank())+
                            theme(legend.key.width = unit(0.2, 'cm'))+
                            geom_richtext(data = rmat, aes(x = 1.75, y = 1.75, label = mylabel),hjust = 0.5, vjust   = 0.5, fill
= NA, label.colour = NA)+
                            scale_colour_manual(name=NULL, labels=c("Male", "Female"), values=c("dark blue","orange"))+
                            geom_point(alpha=0.2, aes(!!!simulate_aes_string(color="Sex")))+
                            scale_x_continuous(limits=c(0,3.5), breaks=c(0,1,2,3))+
                            scale_y_continuous(limits=c(0,3.5), breaks=c(0,1,2,3))+
                            theme(strip.background.x = element_blank())+
                            theme(strip.background.y = element_blank())+
                            theme(strip.text.x = element_text(face="bold", size=rel(1.1)))+
                            theme(strip.text.y = element_text(face="bold", size=rel(1.1)))+
                            labs(x="Time outdoors (hours per day)",y="Time outdoors (hours per day)")+
                            facet_grid(yv~xv)
#plot2
outfile   <- paste0(mydir,"FigureS2_2024-05-05.tiff")
tiff(outfile, width = 22, height = 22, units = "cm", compression = "lzw", res=300)
plot_grid(plot2, ncol=1)
dev.off()


# Correlation in time reading over childhood
# -----------------------------------------

exposure_list            <- c("RB54","RB65","RB77","RB103","RB167SP")
exposure_full            <- c("Age 4.5 years","Age 5.5 years","Age 6.5 years","Age 8.5 years","Age 14.0 years")
dataP                    <- dataYP[which(!is.na(dataYP$Sex)),c("Sex",exposure_list)]
dataP$Sex                <- as.factor(dataP$Sex)
d_aug                    <- PairPlotJG(dataP, exposure_list, "Sex")
d2                       <- d_aug
levels(d2$xv)            <- list("Age 4.5 years"="RB54",  "Age 5.5 years"="RB65",  "Age 6.5 years"="RB77", "Age 8.5
years"="RB103",  "Age 14.0 years"="RB167SP")
levels(d2$yv)            <- list("Age 4.5 years"="RB54",  "Age 5.5 years"="RB65",  "Age 6.5 years"="RB77", "Age 8.5
years"="RB103",  "Age 14.0 years"="RB167SP")
d2$xv                    <- ordered(d2$xv, levels=c("Age 4.5 years","Age 5.5 years","Age 6.5 years","Age 8.5 years","Age 14.0
years"))
d2$yv                    <- ordered(d2$yv, levels=c("Age 4.5 years","Age 5.5 years","Age 6.5 years","Age 8.5 years","Age 14.0
years"))
```

```
rmat            <- as.data.frame(matrix(nrow=(length(exposure_full)^2), ncol=3))
pmat            <- as.data.frame(matrix(nrow=(length(exposure_full)^2), ncol=3))
myrow           <- 1
for (i in 1:(length(exposure_full))){
for (j in 1:(length(exposure_full))){
 ct                 <- cor.test(x=dataYP[,exposure_list[i]], y=dataYP[,exposure_list[j]], method = "spearman", use =
"pairwise.complete.obs")
 rmat[myrow,1]      <- exposure_full[i]
 rmat[myrow,2]      <- exposure_full[j]
 rmat[myrow,3]      <- ct$estimate
 pmat[myrow,1]      <- exposure_full[i]
 pmat[myrow,2]      <- exposure_full[j]
 pmat[myrow,3]      <- ct$p.value
 myrow              <- myrow + 1
}}
names(rmat)         <- c("xv","yv","cm")
rmat$cm             <- sprintf("%.2f",as.numeric(rmat$cm))
rmat$mylabel        <- paste(rmat$cm,"<br>(*P* < 0.001)")
rmat$xv             <- ordered(rmat$xv, levels=c("Age 4.5 years","Age 5.5 years","Age 6.5 years","Age 8.5 years","Age 14.0
years"))
rmat$yv             <- ordered(rmat$yv, levels=c("Age 4.5 years","Age 5.5 years","Age 6.5 years","Age 8.5 years","Age 14.0
years"))

rmat[which(rmat$xv==exposure_full[length(exposure_full)] & rmat$yv==exposure_full[length(exposure_full)]),]$mylabel <- NA
d2[which(d2$xv==exposure_full[length(exposure_full)] & d2$yv==exposure_full[length(exposure_full)]),]              <- NA
for (i in 1:(length(exposure_full))){
for (j in 1:(length(exposure_full))){
 if(j <= i){ rmat[which(rmat$xv==exposure_full[i] & rmat$yv==exposure_full[j]),]$mylabel <- NA
 } else {       d2[which(d2$xv==exposure_full[i] & d2$yv==exposure_full[j]),]              <- NA
 }
}}

plot3                  <- ggplot(data = d2[which(!is.na(d2$xv)),], aes(x=x, y=y))+
                          theme(legend.position=c(0.91,0.09))+
                          theme(legend.title = element_blank())+
                          theme(legend.key.width = unit(0.2, 'cm'))+
                          geom_richtext(data = rmat, aes(x = 1.75, y = 1.75, label = mylabel),hjust = 0.5, vjust   = 0.5, fill
= NA, label.colour = NA)+
                          scale_colour_manual(name=NULL, labels=c("Male", "Female"), values=c("dark blue","orange"))+
                          geom_jitter(alpha=0.1, height=0.2, width=0.2, aes(!!!simulate_aes_string(color="Sex")))+
                          scale_x_continuous(limits=c(0,3.5), breaks=c(0,1,2,3))+
                          scale_y_continuous(limits=c(0,3.5), breaks=c(0,1,2,3))+
                          theme(strip.background.x = element_blank())+
                          theme(strip.background.y = element_blank())+
```

```
                            theme(strip.text.x = element_text(face="bold", size=rel(1.1)))+
                            theme(strip.text.y = element_text(face="bold", size=rel(1.1)))+
                            labs(x="Time reading (hours per day)",y="Time reading (hours per day)")+
                            facet_grid(yv~xv)
#plot3
outfile   <- paste0(mydir,"FigureS3_2024-05-05.tiff")
tiff(outfile, width = 22, height = 22, units = "cm", compression = "lzw", res=300)
plot_grid(plot3, ncol=1)
dev.off()


# Correlation between sleep and duration and other traits
# -------------------------------------------------------

theme_jg <- function (base_size = 10, base_family = "") {
    theme_gray(base_size = base_size, base_family = base_family) %+replace%
        theme(
            axis.text = element_text(colour = "black"),
            axis.title.x = element_blank(),
            axis.title.y = element_text(colour = "black", size=rel(0.8), angle=90, margin = margin(r = 10)),
            axis.text.x = element_text(colour = "black", size=rel(0.8)),
            axis.text.y = element_text(colour = "black", size=rel(0.8), margin = margin(r = 5)),
            plot.title = element_text(face="bold", hjust = 0.5, size=rel(0.8), margin=margin(b = 5)),
            panel.grid.minor = element_blank(),
            panel.grid.major = element_blank(),
            plot.background = element_blank(),
            panel.background = element_rect(fill="white"),
            legend.text = element_text(size=rel(1)),
            legend.title = element_text(size=rel(1), hjust=0),
            legend.key.height = unit(0.4, 'cm'),
            legend.key = element_rect(fill = "transparent", colour = "transparent"),
            plot.margin = margin(0.4, 0.2, 0.4, 0.2, "cm"),
    )
}
theme_set(theme_jg())

exposure_list            <-
c("SLP115","TO103","RB103","Sex","TDIQ","CatOwner","DogOwner","BirthOrder","bedtime_var42_81","scoreSLPpgs","scoreTOSpgs","scoreE
DUpgs")
exposure_full            <- NULL
dataP                    <- dataYP[,exposure_list]
dataP$Sex                <- as.factor(dataP$Sex)
dataP$CatOwner           <- as.factor(dataP$CatOwner)
dataP$DogOwner           <- as.factor(dataP$DogOwner)
```

```
dataP$BirthOrder          <- as.factor(dataP$BirthOrder)
levels(dataP$Sex)         <- list("Male"="1", "Female"="2")
levels(dataP$CatOwner)    <- list("No"="0", "Yes"="1")
levels(dataP$DogOwner)    <- list("No"="0", "Yes"="1")
levels(dataP$BirthOrder) <- list("1"="1", "2"="2", "3"="3","4+"="4")

dataP2                    <- dataP[complete.cases(dataP[,"SLP115"]),]
mymod                     <- t.test(SLP115 ~ Sex, data=dataP2[which(!is.na(dataP2$Sex)),], alternative="two.sided")
labelSex                  <- paste("\u394 =",sprintf("%.2f",mymod$estimate[[2]]-mymod$estimate[[1]])," hours<br>*P* < 0.001")
mymod                     <- summary(aov(SLP115 ~ TDIQ, data=dataP2[which(!is.na(dataP2$TDIQ)),]))
labelTDI                  <- paste("ANOVA<br>*P* = ", sprintf("%.3f",unlist(mymod)[[9]]))
mymod                     <- cor.test(x=dataP2[which(!is.na(dataP2$TO103)),]$SLP115, y=dataP2[which(!is.na(dataP2$TO103)),]$TO103,
method="spearman", exact = FALSE)
labelTO                   <- paste("*r* =", sprintf("%.2f",mymod$estimate), "<br>*P* = ", sprintf("%.3f",mymod$p.value))
mymod                     <- cor.test(x=dataP2[which(!is.na(dataP2$RB103)),]$SLP115, y=dataP2[which(!is.na(dataP2$RB103)),]$RB103,
method="spearman", exact = FALSE)
labelRB                   <- paste("*r* =", sprintf("%.2f",mymod$estimate), "<br>*P* = ", sprintf("%.3f",mymod$p.value))
mymod                     <- t.test(SLP115 ~ DogOwner, data=dataP2[which(!is.na(dataP2$DogOwner)),], alternative="two.sided")
labelDog                  <- paste("\u394 =",sprintf("%.2f",mymod$estimate[[2]]-mymod$estimate[[1]])," hours<br>*P* = ",
sprintf("%.3f",mymod$p.value))
mymod                     <- t.test(SLP115 ~ CatOwner, data=dataP2[which(!is.na(dataP2$CatOwner)),], alternative="two.sided")
labelCat                  <- paste("\u394 =",sprintf("%.2f",mymod$estimate[[2]]-mymod$estimate[[1]])," hours<br>*P* = ",
sprintf("%.3f",mymod$p.value))
mymod                     <- summary(aov(SLP115 ~ BirthOrder, data=dataP2[which(!is.na(dataP2$BirthOrder)),]))
labelBirth                <- paste("ANOVA<br>*P* = ", sprintf("%.3f",unlist(mymod)[[9]]))
mymod                     <- cor.test(x=dataP2[which(!is.na(dataP2$bedtime_var42_81)),]$SLP115,
y=dataP2[which(!is.na(dataP2$bedtime_var42_81)),]$bedtime_var42_81, method="spearman", exact = FALSE)
labelBed                  <- paste("*r* =", sprintf("%.2f",mymod$estimate), "<br>*P* < 0.001")
mymod                     <- cor.test(x=dataP2[which(!is.na(dataP2$scoreSLPpgs)),]$SLP115,
y=dataP2[which(!is.na(dataP2$scoreSLPpgs)),]$scoreSLPpgs, method="spearman", exact = FALSE)
labelPGSslp               <- paste("*r* =", sprintf("%.2f",mymod$estimate), "<br>*P* = ", sprintf("%.3f",mymod$p.value))
mymod                     <- cor.test(x=dataP2[which(!is.na(dataP2$scoreTOSpgs)),]$SLP115,
y=dataP2[which(!is.na(dataP2$scoreTOSpgs)),]$scoreTOSpgs, method="spearman", exact = FALSE)
labelPGStos               <- paste("*r* =", sprintf("%.2f",mymod$estimate), "<br>*P* < 0.001")
mymod                     <- cor.test(x=dataP2[which(!is.na(dataP2$scoreEDUpgs)),]$SLP115,
y=dataP2[which(!is.na(dataP2$scoreEDUpgs)),]$scoreEDUpgs, method="spearman", exact = FALSE)
labelPGSedu               <- paste("*r* =", sprintf("%.2f",mymod$estimate), "<br>*P* < 0.001")

plot2A                    <- ggplot(dataP2[which(!is.na(dataP2$Sex)),], aes(y=SLP115, x=Sex, fill=Sex))+
                             scale_y_continuous(limits=c(7,15), breaks=c(8,10,12,14))+
                             labs(y="Sleep duration at age 9.5 years (hours)")+
                             ggtitle("Gender")+
                             geom_violin(colour="black") +
                             stat_summary(fun.data=mean_sdl, geom="pointrange", colour = "black", linewidth = 1.5, size=1)+
```

```
                            scale_fill_manual(guide="none", values=c("yellow","yellow"))+
                            annotate("richtext", x = 1.5, y = 15, size=3, hjust=0.5, fill = NA, label.colour = NA,
label=labelSex)
plot2B                   <- ggplot(dataP2[which(!is.na(dataP2$DogOwner)),], aes(y=SLP115, x=DogOwner, fill=DogOwner))+
                            scale_y_continuous(limits=c(7,15), breaks=c(8,10,12,14))+
                            theme(axis.title.y = element_blank(), axis.text.y = element_blank())+
                            labs(y="Sleep duration at age 9.5 years (hours)")+
                            ggtitle("Dog owner")+
                            geom_violin(colour="black") +
                            stat_summary(fun.data=mean_sdl, geom="pointrange", colour = "black", linewidth = 1.5, size=1)+
                            scale_fill_manual(guide="none", values=c("light green","light green"))+
                            annotate("richtext", x = 1.5, y = 15, size=3, hjust=0.5, fill = NA, label.colour = NA,
label=labelDog)
plot2C                   <- ggplot(dataP2[which(!is.na(dataP2$CatOwner)),], aes(y=SLP115, x=CatOwner, fill=CatOwner))+
                            scale_y_continuous(limits=c(7,15), breaks=c(8,10,12,14))+
                            theme(axis.title.y = element_blank(), axis.text.y = element_blank())+
                            labs(y="Sleep duration at age 9.5 years (hours)")+
                            ggtitle("Cat owner")+
                            geom_violin(colour="black") +
                            stat_summary(fun.data=mean_sdl, geom="pointrange", colour = "black", linewidth = 1.5, size=1)+
                            scale_fill_manual(guide="none", values=c("light blue","light blue"))+
                            annotate("richtext", x = 1.5, y = 15, size=3, hjust=0.5, fill = NA, label.colour = NA,
label=labelCat)
plot2D                   <- ggplot(dataP2[which(!is.na(dataP2$BirthOrder)),], aes(y=SLP115, x=BirthOrder, fill=BirthOrder))+
                            scale_y_continuous(limits=c(7,15), breaks=c(8,10,12,14))+
                            theme(axis.title.y = element_blank(), axis.text.y = element_blank())+
                            labs(y="Sleep duration at age 9.5 years (hours)")+
                            ggtitle("Birth order")+
                            geom_violin(colour="black") +
                            stat_summary(fun.data=mean_sdl, geom="pointrange", colour = "black", linewidth = 1.5, size=1)+
                            scale_fill_manual(guide="none", values=c("pink","pink","pink","pink"))+
                            annotate("richtext", x = 2.5, y = 15, size=3, hjust=0.5, fill = NA, label.colour = NA,
label=labelBirth)
plot2E                   <- ggplot(dataP2[which(!is.na(dataP2$TDIQ)),], aes(y=SLP115, x=factor(TDIQ), fill=factor(TDIQ)))+
                            scale_y_continuous(limits=c(7,15), breaks=c(8,10,12,14))+
                            theme(axis.title.y = element_blank(), axis.text.y = element_blank())+
                            labs(y="Sleep duration at age 9.5 years (hours)")+
                            ggtitle("Townsend Deprivation Index\n(quintile)")+
                            geom_violin(colour="black") +
                            stat_summary(fun.data=mean_sdl, geom="pointrange", colour = "black", linewidth = 1.5, size=1)+
                            scale_fill_manual(guide="none", values=c("dark orange","dark orange","dark orange","dark
orange","dark orange"))+
                            annotate("richtext", x = 3, y = 15, size=3, hjust=0.5, fill = NA, label.colour = NA, label=labelTDI)
plot2F                   <- ggplot(dataP2[which(!is.na(dataP2$TO103)),], aes(y=SLP115, x=TO103))+
```

```
                              scale_y_continuous(limits=c(7,15), breaks=c(8,10,12,14))+
                              labs(y="Sleep duration at age 9.5 years (hours)")+
                              ggtitle("Time outdoors\n(hours per day)")+
                              geom_point(shape=21, colour="black", fill="yellow",alpha=0.3,
position=position_jitter(width=0.15,height=0.15))+
                              geom_smooth(formula="y~x", colour="black", linewidth=1, method = "lm", se = FALSE)+
                              annotate("richtext", x = 1.8, y = 15, size=3, hjust=0.5, fill = NA, label.colour = NA, label=labelTO)
plot2G                     <- ggplot(dataP2[which(!is.na(dataP2$RB103)),], aes(y=SLP115, x=RB103))+
                              scale_y_continuous(limits=c(7,15), breaks=c(8,10,12,14))+
                              theme(axis.title.y = element_blank(), axis.text.y = element_blank())+
                              labs(y="Sleep duration at age 9.5 years (hours)")+
                              ggtitle("Time reading\n(hours per day)")+
                              geom_point(shape=21, colour="black", fill="light green",alpha=0.3,
position=position_jitter(width=0.15,height=0.15))+
                              geom_smooth(formula="y~x", colour="black", linewidth=1, method = "lm", se = FALSE)+
                              annotate("richtext", x = 1.5, y = 15, size=3, hjust=0.5, fill = NA, label.colour = NA, label=labelRB)
plot2H                     <- ggplot(dataP2[which(!is.na(dataP2$bedtime_var42_81)),], aes(y=SLP115, x=bedtime_var42_81))+
                              scale_y_continuous(limits=c(7,15), breaks=c(8,10,12,14))+
                              theme(axis.title.y = element_blank(), axis.text.y = element_blank())+
                              labs(y="Sleep duration at age 9.5 years (hours)")+
                              ggtitle("Bedtime variation\n(arbitrary units)")+
                              scale_x_continuous(limits=c(0,2.5), breaks=c(0,1,2))+
                              geom_point(shape=21, colour="black", fill="light blue",alpha=0.3,
position=position_jitter(width=0.15,height=0.15))+
                              geom_smooth(formula="y~x", colour="black", linewidth=1, method = "lm", se = FALSE)+
                              annotate("richtext", x = 1.2, y = 15, size=3, hjust=0.5, fill = NA, label.colour = NA,
label=labelBed)
plot2I                     <- ggplot(dataP2[which(!is.na(dataP2$bedtime_var42_81)),], aes(y=SLP115, x=scoreSLPpgs))+
                              scale_y_continuous(limits=c(7,15), breaks=c(8,10,12,14))+
                              theme(axis.title.y = element_blank(), axis.text.y = element_blank())+
                              labs(y="Sleep duration at age 9.5 years (hours)")+
                              ggtitle("PGS for sleep duration\n(arbitrary units)")+
                              scale_x_continuous(limits=c(-3.2,3.2), breaks=c(-2,0,2))+
                              geom_point(shape=21, colour="black", fill="pink",alpha=0.3,
position=position_jitter(width=0.15,height=0.15))+
                              geom_smooth(formula="y~x", colour="black", linewidth=1, method = "lm", se = FALSE)+
                              annotate("richtext", x = 0, y = 15, size=3, hjust=0.5, fill = NA, label.colour = NA,
label=labelPGSslp)
plot2J                     <- ggplot(dataP2[which(!is.na(dataP2$bedtime_var42_81)),], aes(y=SLP115, x=scoreTOSpgs))+
                              scale_y_continuous(limits=c(7,15), breaks=c(8,10,12,14))+
                              theme(axis.title.y = element_blank(), axis.text.y = element_blank())+
                              labs(y="Sleep duration at age 9.5 years (hours)")+
                              ggtitle("PGS for time outdoors\n(arbitrary units)")+
                              scale_x_continuous(limits=c(-3.2,3.2), breaks=c(-2,0,2))+
```

```
                            geom_point(shape=21, colour="black", fill="dark orange",alpha=0.3,
position=position_jitter(width=0.15,height=0.15))+
                            geom_smooth(formula="y~x", colour="black", linewidth=1, method = "lm", se = FALSE)+
                            annotate("richtext", x = 0, y = 15, size=3, hjust=0.5, fill = NA, label.colour = NA,
label=labelPGStos)
plot2K                   <- ggplot(dataP2[which(!is.na(dataP2$bedtime_var42_81)),], aes(y=SLP115, x=scoreEDUpgs))+
                            scale_y_continuous(limits=c(7,15), breaks=c(8,10,12,14))+
                            theme(axis.title.y = element_blank(), axis.text.y = element_blank())+
                            labs(y="Sleep duration at age 9.5 years (hours)")+
                            ggtitle("PGS for EduYears\n(arbitrary units)")+
                            scale_x_continuous(limits=c(-3.2,3.2), breaks=c(-2,0,2))+
                            geom_point(shape=21, colour="black", fill="lightslateblue",alpha=0.3,
position=position_jitter(width=0.15,height=0.15))+
                            geom_smooth(formula="y~x", colour="black", linewidth=1, method = "lm", se = FALSE)+
                            annotate("richtext", x = 0, y = 15, size=3, hjust=0.5, fill = NA, label.colour = NA,
label=labelPGSedu)

plot4     <- plot_grid(plot2A,plot2B,plot2C,plot2D,plot2E, NULL,
                       plot2F,plot2G,plot2H,plot2I,plot2J,plot2K, ncol=6, align="h", rel_widths=c(1.4,1,1,1,1,1,1.4,1,1,1,1,1))
outfile   <- paste0(mydir,"FigureS5_2024-05-05.tiff")
tiff(outfile, width = 22, height = 22, units = "cm", compression = "lzw", res=300)
plot_grid(plot4, ncol=1)
dev.off()


##########################################################################################################################

# Is sleep duration/time reading/time outdoors associated with refractive error at age 15 years? Observational in full cohort
# ----------------------------------------------------------------------------------------------------------------------

outcome_list             <- c("Yr15_avMSE")
num_outcomes             <- length(outcome_list)
exposure_list            <- c("SLP42","SLP57","SLP69","SLP81","SLP115","SLP140","SLP186SP",
                              "TO38","TO54","TO65","TO77","TO103","TO167SP","TO198SP","TO22yrSP","TO26yrSP",
                              "RB54","RB65","RB77","RB103","RB167SP","RB198SP")
num_exposures            <- length(exposure_list)

results                  <- as.data.frame(matrix(ncol=11, nrow=(num_outcomes*num_exposures)))
names(results)           <- c("Outcome","Exposure","ExposureAge","BETA","SE","LCI","UCI","P","N","Adjusted","SelfRep")
myrow                    <- 1
for(e in 1:num_exposures){
  formExposure           <- as.character(exposure_list[e])
  selfrep                <- 0
  if(right(formExposure, n=2)=="SP"){
```

```
    selfrep                 <- 1
    formExposure            <- left(formExposure, (nchar(formExposure) - 2))}
  if(left(formExposure, n=2)=="TO"){
    formExp                 <- "Time outdoors"
    formAge                 <- as.character(substr(formExposure,3,nchar(formExposure)))}
  if(left(formExposure, n=2)=="RB"){
    formExp                 <- "Time reading"
    formAge                 <- as.character(substr(formExposure,3,nchar(formExposure)))}
  if(left(formExposure, n=3)=="SLP"){
    formExp                 <- "Sleep duration"
    formAge                 <- as.character(substr(formExposure,4,nchar(formExposure)))}
  formExposure            <- as.character(exposure_list[e])
for(f in 1:num_outcomes){
  formOut                 <- as.character(outcome_list[f])
  formNonAdj              <- as.formula(paste(formOut,"~",formExposure))
  formAdj                 <- as.formula(paste(formOut,"~",formExposure,"+ Sex + TDIQ + mat_age"))
  myMod                   <- summary(lm(formNonAdj, data = dataYP))
  myModAdj                <- summary(lm(formAdj, data = dataYP))
  results[myrow,1]        <- formOut
  results[myrow,2]        <- formExp
  results[myrow,3]        <- formAge
  results[myrow,4]        <- myMod$coefficients[2,1]
  results[myrow,5]        <- myMod$coefficients[2,2]
  results[myrow,6]        <- myMod$coefficients[2,1] - (1.96*myMod$coefficients[2,2])
  results[myrow,7]        <- myMod$coefficients[2,1] + (1.96*myMod$coefficients[2,2])
  results[myrow,8]        <- myMod$coefficients[2,4]
  results[myrow,9]        <- myMod$df[1] + myMod$df[2]
  results[myrow,10]       <- 0
  results[myrow,11]       <- selfrep
  myrow                   <- myrow + 1
  results[myrow,1]        <- formOut
  results[myrow,2]        <- formExp
  results[myrow,3]        <- formAge
  results[myrow,4]        <- myModAdj$coefficients[2,1]
  results[myrow,5]        <- myModAdj$coefficients[2,2]
  results[myrow,6]        <- myModAdj$coefficients[2,1] - (1.96*myModAdj$coefficients[2,2])
  results[myrow,7]        <- myModAdj$coefficients[2,1] + (1.96*myModAdj$coefficients[2,2])
  results[myrow,8]        <- myModAdj$coefficients[2,4]
  results[myrow,9]        <- myModAdj$df[1] + myModAdj$df[2]
  results[myrow,10]       <- 1
  results[myrow,11]       <- selfrep
  myrow                   <- myrow + 1
}
}
```

```
results

results2                 <- results[which(results$Adjusted==1),]
results2$Exposure        <- as.factor(results2$Exposure)
num_exps                 <- length(levels(results2$Exposure))
myrow                    <- 1
results3                 <- results2[0,]
for (n in 1:num_exps){
  myexposure                           <- levels(results2$Exposure)[n]
  myrows                               <-
results2[which(results2$Exposure==myexposure),c("Outcome","ExposureAge","N","BETA","LCI","UCI")]
  myextra                              <- nrow(myrows)
  results3[myrow,]                     <- NA
  results3$Exposure[myrow]             <- myexposure
  results3[(myrow + 1):(myrow + myextra),c("Outcome","ExposureAge","N","BETA","LCI","UCI")] <- myrows
  myrow                                <- myrow + myextra + 1
  }

results3$ExposureAge         <- as.factor(results3$ExposureAge)
levels(results3$ExposureAge) <- list("8.5"="103", "9.5"="115", "11.5"="140", "14.0"="167", "15.5"="186", "16.5"="198",
"22.0"="22yr", "26.0"="26yr",
                                 "3.0"="38", "3.0"="42", "4.5"="54", "4.5"="57", "5.5"="65", "5.5"="69", "6.5"="77", "6.5"="81")
results3$ExposureAge         <- ordered(results3$ExposureAge,
levels=c("26.0","22.0","16.5","15.5","14.0","11.5","9.5","8.5","6.5","5.5","4.5","3.0"))
results4                     <- results3[which(!results3$ExposureAge %in% c("26.0","22.0","16.5","15.5")),]

names(results4)[names(results4) == "Exposure"]    <- "Activity (hours per day)"
names(results4)[names(results4) == "ExposureAge"] <- "Age (years)"

results$ExposureAge          <- as.factor(results$ExposureAge)
levels(results$ExposureAge)  <- list("8.5"="103", "9.5"="115", "11.5"="140", "14.0"="167", "15.5"="186", "16.5"="198",
"22.0"="22yr", "26.0"="26yr",
                                 "3.0"="38", "3.0"="42", "4.5"="54", "4.5"="57", "5.5"="65", "5.5"="69", "6.5"="77", "6.5"="81")
results5                     <- results[which(!results$ExposureAge %in% c("26.0","22.0","16.5","15.5")),]

outfile                      <- paste0(mydir,"FigureS4_2024-05-05.png")
outcsv                       <- paste0(mydir,"TableS1_2024-05-05.csv")
write.csv(results5, file=outcsv, row.names=FALSE)

theme_set(theme_classic())
forester(left_side_data = results4[,c("Activity (hours per day)","Age (years)","N")],
           estimate_col_name="Refractive error in diopters (95% CI)",
           estimate = results4$BETA,
           ci_low = results4$LCI,
```

```
           ci_high = results4$UCI,
           estimate_precision = 2,
           display = TRUE,
           xlim = c(-0.5, 0.5),
           null_line_at = 0,
           font_family = "sans",
           render_as="png",
           file_path=outfile)

# Is sleep duration/time reading/time outdoors associated with refractive error at age 15 years? Observational in cross-sectional sample
# ----------------------------------------------------------------------------------------------------------

form3NonAdj              <- as.formula("Yr15_avMSE ~ SLP115 + RB103 + TO103")
form3Adj                 <- as.formula("Yr15_avMSE ~ SLP115 + RB103 + TO103 + Sex + TDIQ + mat_age")
myMod3                   <- summary(lm(form3NonAdj, data = dataYPW))
myMod3Adj                <- summary(lm(form3Adj, data = dataYPW))

outcome_list             <- c("Yr15_avMSE")
num_outcomes             <- length(outcome_list)
exposure_list            <- c("SLP115","TO103","RB103")
num_exposures            <- length(exposure_list)

results                  <- as.data.frame(matrix(ncol=10, nrow=(num_outcomes*num_exposures)))
names(results)           <- c("Outcome","Exposure","BETA","SE","LCI","UCI","P","N","Adjusted","Combined")
myrow                    <- 1
for(e in 1:num_exposures){
  formExposure           <- exposure_list[e]
for(f in 1:num_outcomes){
  formOut                <- outcome_list[f]
  formNonAdj             <- as.formula(paste(formOut,"~",formExposure))
  formAdj                <- as.formula(paste(formOut,"~",formExposure,"+ Sex + TDIQ"))
  myMod                  <- summary(lm(formNonAdj, data = dataYPW))
  myModAdj               <- summary(lm(formAdj, data = dataYPW))
  results[myrow,1]       <- formOut
  results[myrow,2]       <- formExposure
  results[myrow,3]       <- myMod$coefficients[2,1]
  results[myrow,4]       <- myMod$coefficients[2,2]
  results[myrow,5]       <- myMod$coefficients[2,1] - (1.96*myMod$coefficients[2,2])
  results[myrow,6]       <- myMod$coefficients[2,1] + (1.96*myMod$coefficients[2,2])
  results[myrow,7]       <- myMod$coefficients[2,4]
  results[myrow,8]       <- myMod$df[1] + myMod$df[2]
  results[myrow,9]       <- 0
```

```
  results[myrow,10]       <- 0
  myrow                   <- myrow + 1
  results[myrow,1]        <- formOut
  results[myrow,2]        <- formExposure
  results[myrow,3]        <- myModAdj$coefficients[2,1]
  results[myrow,4]        <- myModAdj$coefficients[2,2]
  results[myrow,5]        <- myModAdj$coefficients[2,1] - (1.96*myModAdj$coefficients[2,2])
  results[myrow,6]        <- myModAdj$coefficients[2,1] + (1.96*myModAdj$coefficients[2,2])
  results[myrow,7]        <- myModAdj$coefficients[2,4]
  results[myrow,8]        <- myModAdj$df[1] + myModAdj$df[2]
  results[myrow,9]        <- 1
  results[myrow,10]       <- 0
  myrow                   <- myrow + 1

  outrow                  <- grep(formExposure,row.names(myMod3$coefficients))
  results[myrow,1]        <- "Yr15_avMSE"
  results[myrow,2]        <- formExposure
  results[myrow,3]        <- myMod3$coefficients[outrow,1]
  results[myrow,4]        <- myMod3$coefficients[outrow,2]
  results[myrow,5]        <- myMod3$coefficients[outrow,1] - (1.96*myMod3$coefficients[outrow,2])
  results[myrow,6]        <- myMod3$coefficients[outrow,1] + (1.96*myMod3$coefficients[outrow,2])
  results[myrow,7]        <- myMod3$coefficients[outrow,4]
  results[myrow,8]        <- myMod3$df[1] + myMod3$df[2]
  results[myrow,9]        <- 0
  results[myrow,10]       <- 1
  myrow                   <- myrow + 1
  outrow                  <- grep(formExposure,row.names(myMod3Adj$coefficients))
  results[myrow,1]        <- "Yr15_avMSE"
  results[myrow,2]        <- formExposure
  results[myrow,3]        <- myMod3Adj$coefficients[outrow,1]
  results[myrow,4]        <- myMod3Adj$coefficients[outrow,2]
  results[myrow,5]        <- myMod3Adj$coefficients[outrow,1] - (1.96*myMod3Adj$coefficients[outrow,2])
  results[myrow,6]        <- myMod3Adj$coefficients[outrow,1] + (1.96*myMod3Adj$coefficients[outrow,2])
  results[myrow,7]        <- myMod3Adj$coefficients[outrow,4]
  results[myrow,8]        <- myMod3Adj$df[1] + myMod3Adj$df[2]
  results[myrow,9]        <- 1
  results[myrow,10]       <- 1
  myrow                   <- myrow + 1
}
}
results

results2                  <- results[which(results$Adjusted==1),]
combined_obs_cs           <- results2
```

```
# Is sleep duration/time reading/time outdoors associated with refractive error trajectory? Observational in longitudinal sample
# ------------------------------------------------------------------------------------------------------------------------------

dataYPL$AgeP1          <- scale(dataYPL$Age)
dataYPL$AgeP2          <- scale((dataYPL$Age)^2)
dataYPL$AgeP3          <- scale((dataYPL$Age)^3)
dataYPL$AgeP4          <- scale((dataYPL$Age)^4)
formNonAdj             <- as.formula("avMSE ~ AgeP1 +AgeP2 + AgeP3 +                       Age:SLP115 + Age:RB103 + Age:TO103")
formAdj                <- as.formula("avMSE ~ AgeP1 +AgeP2 + AgeP3 + Sex + TDIQ + mat_age + Age:SLP115 + Age:RB103 + Age:TO103")
myMod3                 <- summary(plm(formNonAdj, data = dataYPL, index = c("cidB3675","Visit"), model = "random"))
myMod3Adj              <- summary(plm(formAdj,    data = dataYPL, index = c("cidB3675","Visit"), model = "random"))

exposure_list            <- c("SLP115","TO103","RB103")
num_exposures            <- length(exposure_list)

results                  <- as.data.frame(matrix(ncol=10, nrow=(2*num_exposures)))
names(results)           <- c("Outcome","Exposure","BETA_INT","SE_INT","LCI_INT","UCI_INT","P_INT","N","Adjusted","Combined")
myrow                    <- 1
for(e in 1:num_exposures){
  formExposure           <- exposure_list[e]
  formNonAdj             <- as.formula(paste0("avMSE ~ AgeP1 +AgeP2 + AgeP3 +                       Age:",formExposure))
  formAdj                <- as.formula(paste0("avMSE ~ AgeP1 +AgeP2 + AgeP3 + Sex + TDIQ + mat_age + Age:",formExposure))
  myMod                  <- summary(plm(formNonAdj, data = dataYPL, index = c("cidB3675","Visit"), model = "random"))
  myModAdj               <- summary(plm(formAdj,    data = dataYPL, index = c("cidB3675","Visit"), model = "random"))
  outrow                 <- grep(formExposure,row.names(myMod$coefficients))
  results[myrow,1]       <- "Refractive error trajectory"
  results[myrow,2]       <- formExposure
  results[myrow,3]       <- myMod$coefficients[outrow,1]
  results[myrow,4]       <- myMod$coefficients[outrow,2]
  results[myrow,5]       <- myMod$coefficients[outrow,1] - (1.96*myMod$coefficients[outrow,2])
  results[myrow,6]       <- myMod$coefficients[outrow,1] + (1.96*myMod$coefficients[outrow,2])
  results[myrow,7]       <- myMod$coefficients[outrow,4]
  results[myrow,8]      <- 3086
  results[myrow,9]       <- 0
  results[myrow,10]      <- 0
  myrow                  <- myrow + 1
  outrow                 <- grep(formExposure,row.names(myModAdj$coefficients))
  results[myrow,1]       <- "Refractive error trajectory"
  results[myrow,2]       <- formExposure
  results[myrow,3]       <- myModAdj$coefficients[outrow,1]
  results[myrow,4]       <- myModAdj$coefficients[outrow,2]
  results[myrow,5]       <- myModAdj$coefficients[outrow,1] - (1.96*myModAdj$coefficients[outrow,2])
```

```
  results[myrow,6]       <- myModAdj$coefficients[outrow,1] + (1.96*myModAdj$coefficients[outrow,2])
  results[myrow,7]       <- myModAdj$coefficients[outrow,4]
  results[myrow,8]       <- 3086
  results[myrow,9]       <- 1
  results[myrow,10]      <- 0
  myrow                  <- myrow + 1

  outrow                 <- grep(formExposure,row.names(myMod3$coefficients))
  results[myrow,1]       <- "Refractive error trajectory"
  results[myrow,2]       <- formExposure
  results[myrow,3]       <- myMod3$coefficients[outrow,1]
  results[myrow,4]       <- myMod3$coefficients[outrow,2]
  results[myrow,5]       <- myMod3$coefficients[outrow,1] - (1.96*myMod3$coefficients[outrow,2])
  results[myrow,6]       <- myMod3$coefficients[outrow,1] + (1.96*myMod3$coefficients[outrow,2])
  results[myrow,7]       <- myMod3$coefficients[outrow,4]
  results[myrow,8]       <- 3086
  results[myrow,9]       <- 0
  results[myrow,10]      <- 1
  myrow                  <- myrow + 1
  outrow                 <- grep(formExposure,row.names(myMod3Adj$coefficients))
  results[myrow,1]       <- "Refractive error trajectory"
  results[myrow,2]       <- formExposure
  results[myrow,3]       <- myMod3Adj$coefficients[outrow,1]
  results[myrow,4]       <- myMod3Adj$coefficients[outrow,2]
  results[myrow,5]       <- myMod3Adj$coefficients[outrow,1] - (1.96*myMod3Adj$coefficients[outrow,2])
  results[myrow,6]       <- myMod3Adj$coefficients[outrow,1] + (1.96*myMod3Adj$coefficients[outrow,2])
  results[myrow,7]       <- myMod3Adj$coefficients[outrow,4]
  results[myrow,8]       <- 3086
  results[myrow,9]       <- 1
  results[myrow,10]      <- 1
  myrow                  <- myrow + 1
}
results

results2                 <- results[which(results$Adjusted==1),]
combined_obs_long         <- results2
myrow                    <- 1
results3                 <- results2[0,]
for (n in 1:num_exposures){
  myexposure                                <- exposure_list[n]
  myrows                                    <-
results2[which(results2$Exposure==myexposure),c("Outcome","N","BETA_INT","LCI_INT","UCI_INT","Combined")]
  myextra                                   <- nrow(myrows)
  results3[myrow,]                          <- NA
```

```
  results3$Exposure[myrow]                 <- myexposure
  results3[(myrow + 1):(myrow + myextra),c("Outcome","N","BETA_INT","LCI_INT","UCI_INT","Combined")] <- myrows
  myrow                                    <- myrow + myextra + 1
  }

results3$Exposure            <- as.character(results3$Exposure)
results3$Exposure            <- c("Sleep duration at age 9.5 years",NA,NA,"Time outdoors at age 8.5 years",NA,NA,"Time reading at
age 8.5 years",NA,NA)
results3$Combined            <- as.character(results3$Combined)
results3$Combined            <- c(NA, "Sleep duration", "Sleep, outdoors and reading", NA, "Time outdoors", "Sleep, outdoors and
reading", NA, "Time reading", "Sleep, outdoors and reading")
results4                     <- results3
names(results4)[names(results4) == "Exposure"]    <- "Activity (hours per day)"
names(results4)[names(results4) == "Combined"]    <- "Risk factors in model"

outcsv                       <- paste0(mydir,"TableS2_2024-07-17.csv")
write.csv(results, file=outcsv, row.names=FALSE)

################################################################################################################################

# IV first stage F-statistics
# ----------------------------

exposure_list            <- c("SLP115","TO103","RB103")
num_exposures            <- length(exposure_list)
iv_list                  <- c("CatOwner","DogOwner","BirthOrder","bedtime_var42_81","scoreTOSpgs","scoreEDUpgs","scoreSLPpgs")
num_ivs                  <- length(iv_list)
cov_list                 <- c("Sex","TDIQ","mat_age","PC1","PC2","PC3")

results                  <- as.data.frame(matrix(nrow=num_ivs,ncol=(1 + num_exposures) ))
names(results)           <- c("IV",exposure_list)
results$IV               <- iv_list
for(e in 1:num_exposures){
  formExposure           <- exposure_list[e]
for(f in 1:num_ivs){
  formIV                 <- iv_list[f]
  formAdj                <- as.formula(paste0("Yr15_avMSE ~ ", paste(cov_list, collapse=" + "), " | ", formExposure, " ~ ",
formIV))
  myModAdj               <- summary(feols(formAdj, data = dataYPW, notes=FALSE))
  formDir                <- as.formula(paste0(formExposure, " ~ ", formIV, " + ", paste(cov_list, collapse=" + ")))
  myModDir               <- summary(lm(formDir, data = dataYPW))
  a                      <- sprintf("%.2f", fitstat(myModAdj,type="ivf")[[1]]$stat)
  b                      <- ifelse(myModDir$coefficients[2,1]>=0, "[+]", "[-]")
  results[f,(1 + e)]     <- paste(a,b)
```

```
}
}
results

outcsv                    <- paste0(mydir,"Table2_2024-05-05.csv")
write.csv(results, file=outcsv, row.names=FALSE)


# IV associations with exposure variables
# ---------------------------------------

exposure_list             <- sort(c("RB103","SLP115","TO103"))
num_exposures             <- length(exposure_list)
iv_list                   <- c("CatOwner","DogOwner","BirthOrder","bedtime_var42_81","scoreTOSpgs","scoreEDUpgs","scoreSLPpgs")
num_ivs                   <- length(iv_list)
cov_list                  <- c("Sex","TDIQ","mat_age","PC1","PC2","PC3")

results                   <- as.data.frame(matrix(nrow=num_ivs,ncol=(1 + (3*num_exposures)) ))
names(results)[1]         <- "IV"
names(results)[2:ncol(results)] <- paste(sort(rep(exposure_list,3)), c("_BETA","_95%CI","_P"),sep="")
results$IV                <- iv_list
for(e in 1:num_exposures){
  formExposure            <- exposure_list[e]
for(f in 1:num_ivs){
  formIV                  <- iv_list[f]
  formDir                 <- as.formula(paste0(formExposure, " ~ ", formIV, " + ", paste(cov_list, collapse=" + ")))
  myModDir                <- summary(lm(formDir, data = dataYPW))
  a                       <- sprintf("%.2f", myModDir$coefficients[2,1])
  b                       <- sprintf("%.2f", myModDir$coefficients[2,1] - (1.96*myModDir$coefficients[2,2]))
  c                       <- sprintf("%.2f", myModDir$coefficients[2,1] + (1.96*myModDir$coefficients[2,2]))
  d                       <- sprintf("%.3f", myModDir$coefficients[2,4])
  results[f,((3*e)-1)]    <- a
  results[f,((3*e))]      <- paste("(",b," to ",c,")",sep="")
  results[f,((3*e)+1)]    <- d
}
}
results

outcsv                    <- paste0(mydir,"Table3_2024-05-05.csv")
write.csv(results, file=outcsv, row.names=FALSE)

##############################################################################################################################
#######
```

```
# Multiple IV analysis: Cross-sectional sample
# ----------------------------------------------

form3NonAdj              <- as.formula("Yr15_avMSE ~ 1                                    | SLP115 + TO103 + RB103 ~
                                        CatOwner + DogOwner + BirthOrder + bedtime_var42_81 + scoreTOSpgs +
scoreEDUpgs + scoreSLPpgs")
form3Adj                 <- as.formula("Yr15_avMSE ~ Sex + TDIQ + mat_age + PC1 + PC2 + PC3 | SLP115 + TO103 + RB103 ~
                                        CatOwner + DogOwner + BirthOrder + bedtime_var42_81 + scoreTOSpgs +
scoreEDUpgs + scoreSLPpgs")

myMod3                   <- summary(feols(form3NonAdj, data = dataYPW, notes=FALSE))
myMod3Adj                <- summary(feols(form3Adj,    data = dataYPW, notes=FALSE))

results                  <- as.data.frame(matrix(nrow=1,ncol=13))
names(results)           <- c("Outcome","Exposure","IV","F","BETA","SE","LCI","UCI","P","N","WHpval","Adjusted","Combined")
myrow                    <- 1

formOut                  <- c("Yr15_avMSE")
formIV                   <- c("CatOwner + DogOwner + BirthOrder + bedtime_var42_81 + scoreTOSpgs + scoreEDUpgs + scoreSLPpgs")
exposure_list            <- c("SLP115","TO103","RB103")
num_exposures            <- length(exposure_list)
cov_list                 <- c("Sex","TDIQ","mat_age","PC1","PC2","PC3")

for(e in 1:num_exposures){
  formExposure           <- exposure_list[e]
  otherCovs              <- exposure_list[-e]
  formNonAdj             <- as.formula(paste0("Yr15_avMSE ~ 1 | ",formExposure,"  ~ ", formIV))
  formAdj                <- as.formula(paste0("Yr15_avMSE ~ ", paste(cov_list, collapse=" + "), " + ", paste(otherCovs,
collapse=" + "), " | ",formExposure," ~ ",formIV))
  myMod                  <- summary(feols(formNonAdj, data = dataYPW, notes=FALSE))
  myModAdj               <- summary(feols(formAdj, data = dataYPW, notes=FALSE))
  outrow                 <- grep(formExposure,row.names(myMod$coeftable))
  results[myrow,1]       <- formOut
  results[myrow,2]       <- formExposure
  results[myrow,3]       <- "All seven IVs"
  results[myrow,4]       <- sprintf("%.2f", fitstat(myMod,type="ivf",simplify=TRUE)$stat)
  results[myrow,5]       <- myMod$coeftable[outrow,1]
  results[myrow,6]       <- myMod$coeftable[outrow,2]
  results[myrow,7]       <- myMod$coeftable[outrow,1] - (1.96*myMod$coeftable[outrow,2])
  results[myrow,8]       <- myMod$coeftable[outrow,1] + (1.96*myMod$coeftable[outrow,2])
  results[myrow,9]       <- myMod$coeftable[outrow,4]
  results[myrow,10]      <- myMod$nobs
  results[myrow,11]      <- myMod$iv_wh$p
  results[myrow,12]      <- 0
```

```
results[myrow,13]       <- 0
myrow                   <- myrow + 1
outrow                  <- grep(formExposure,row.names(myModAdj$coeftable))
results[myrow,1]        <- formOut
results[myrow,2]        <- formExposure
results[myrow,3]        <- "All seven IVs"
results[myrow,4]        <- sprintf("%.2f", fitstat(myModAdj,type="ivf",simplify=TRUE)$stat)
results[myrow,5]        <- myModAdj$coeftable[outrow,1]
results[myrow,6]        <- myModAdj$coeftable[outrow,2]
results[myrow,7]        <- myModAdj$coeftable[outrow,1] - (1.96*myModAdj$coeftable[outrow,2])
results[myrow,8]        <- myModAdj$coeftable[outrow,1] + (1.96*myModAdj$coeftable[outrow,2])
results[myrow,9]        <- myModAdj$coeftable[outrow,4]
results[myrow,10]       <- myModAdj$nobs
results[myrow,11]       <- myModAdj$iv_wh$p
results[myrow,12]       <- 1
results[myrow,13]       <- 0
myrow                   <- myrow + 1

outrow                  <- grep(formExposure,row.names(myMod3$coeftable))
frow                    <- grep(formExposure,names(fitstat(myMod3,type="ivf")))
results[myrow,1]        <- formOut
results[myrow,2]        <- formExposure
results[myrow,3]        <- "All seven IVs"
results[myrow,4]        <- sprintf("%.2f", fitstat(myMod3,type="ivf")[[frow]]$stat)
results[myrow,5]        <- myMod3$coeftable[outrow,1]
results[myrow,6]        <- myMod3$coeftable[outrow,2]
results[myrow,7]        <- myMod3$coeftable[outrow,1] - (1.96*myMod3$coeftable[outrow,2])
results[myrow,8]        <- myMod3$coeftable[outrow,1] + (1.96*myMod3$coeftable[outrow,2])
results[myrow,9]        <- myMod3$coeftable[outrow,4]
results[myrow,10]       <- myMod3$nobs
results[myrow,11]       <- myMod3$iv_wh$p
results[myrow,12]       <- 0
results[myrow,13]       <- 1
myrow                   <- myrow + 1
outrow                  <- grep(formExposure,row.names(myMod3Adj$coeftable))
results[myrow,1]        <- formOut
results[myrow,2]        <- formExposure
results[myrow,3]        <- "All seven IVs"
results[myrow,4]        <- sprintf("%.2f", fitstat(myMod3Adj,type="ivf")[[frow]]$stat)
results[myrow,5]        <- myMod3Adj$coeftable[outrow,1]
results[myrow,6]        <- myMod3Adj$coeftable[outrow,2]
results[myrow,7]        <- myMod3Adj$coeftable[outrow,1] - (1.96*myMod3Adj$coeftable[outrow,2])
results[myrow,8]        <- myMod3Adj$coeftable[outrow,1] + (1.96*myMod3Adj$coeftable[outrow,2])
results[myrow,9]        <- myMod3Adj$coeftable[outrow,4]
```

```
  results[myrow,10]      <- myMod3Adj$nobs
  results[myrow,11]      <- myMod3Adj$iv_wh$p
  results[myrow,12]      <- 1
  results[myrow,13]      <- 1
  myrow                  <- myrow + 1
}

results2                 <- results[which(results$Adjusted==1),]
combined_iv_cs           <- results2

myrow                    <- 1
results3                 <- results2[0,]
for (n in 1:num_exposures){
  myexposure                            <- exposure_list[n]
  myrows                                <-
results2[which(results2$Exposure==myexposure),c("Exposure","F","BETA","LCI","UCI","Combined")]
  myextra                               <- nrow(myrows)
  results3[myrow,]                      <- NA
  results3$Exposure[myrow]              <- myexposure
  results3[(myrow + 1):(myrow + myextra),c("Exposure","F","BETA","LCI","UCI","Combined")] <- myrows
  myrow                                 <- myrow + myextra + 1
  }

results3$Exposure            <- as.character(results3$Exposure)
results3$Exposure            <- c("Sleep duration at age 9.5 years",NA,NA,"Time outdoors at age 8.5 years",NA,NA,"Time reading at
age 8.5 years",NA,NA)
results3$Combined            <- as.character(results3$Combined)
results3$Combined            <- c(NA, "Sleep duration", "Sleep, outdoors and reading", NA, "Time outdoors", "Sleep, outdoors and
reading", NA, "Time reading", "Sleep, outdoors and reading")
results4                     <- results3
names(results4)[names(results4) == "Exposure"]    <- "Activity (hours per day)"
names(results4)[names(results4) == "F"]           <- "F-stat (1st stage)"
names(results4)[names(results4) == "Combined"]    <- "Risk factors in model"

outcsv                       <- paste0(mydir,"TableS3_2024-07-17.csv")
write.csv(results, file=outcsv, row.names=FALSE)

# Combined plot for cross-sectional observation and IV analyses
# ---------------------------------------------------------------

combined_obs_cs$IV     <- as.character("-")
combined_obs_cs$F      <- as.character("...")
combined_obs_cs$WHpval <- as.character("-")
combined_cs            <- rbind(combined_obs_cs, combined_iv_cs)
```

```
myrow                    <- 1
results3                 <- combined_cs[0,]
for (n in 1:num_exposures){
  myexposure                                <- exposure_list[n]
  myrows                                    <-
combined_cs[which(combined_cs$Exposure==myexposure),c("Exposure","F","BETA","LCI","UCI","Combined")]
  myextra                                   <- nrow(myrows)
  results3[myrow,]                          <- NA
  results3$Exposure[myrow]                  <- myexposure
  results3[(myrow + 1):(myrow + myextra),c("Exposure","F","BETA","LCI","UCI","Combined")] <- myrows
  myrow                                     <- myrow + myextra + 1
  }

results3$Exposure            <- as.character(results3$Exposure)
results3$Exposure            <- c("Sleep duration at age 9.5 years",NA,NA,NA,NA,
                                  "Time outdoors at age 8.5 years",NA,NA,NA,NA,
                                  "Time reading at age 8.5 years",NA,NA,NA,NA)
results3$Combined            <- as.character(results3$Combined)
results3$Combined            <- c(NA, "Sleep duration", "Sleep, outdoors and reading", "Sleep duration", "Sleep, outdoors and
reading",
                                  NA, "Time outdoors", "Sleep, outdoors and reading",  "Time outdoors", "Sleep, outdoors and
reading",
                                  NA, "Time reading", "Sleep, outdoors and reading", "Time reading", "Sleep, outdoors and
reading")
results4                     <- results3
results4$Analysis            <- rep(c("","Observational","Observational","IV","IV"),3)
results4$MyColour            <- rep(c("","dark red","dark red","blue","blue"),3)
names(results4)[names(results4) == "Exposure"]    <- "Risk factor (hours per day)"
names(results4)[names(results4) == "F"]           <- "F-stat"
names(results4)[names(results4) == "Combined"]    <- "Risk factors in model"

outfile                      <- paste0(mydir,"Figure2_2024-07-17.png")

forester(left_side_data = results4[,c("Risk factor (hours per day)","Analysis","Risk factors in model","F-stat")],
           estimate_col_name="Refractive error (D)(95% CI)",
           estimate = results4$BETA,
           ci_low = results4$LCI,
           ci_high = results4$UCI,
           estimate_precision = 2,
           display = TRUE,
           xlim = c(-2.5, 2.5),
           null_line_at = 0,
           font_family = "sans",
```

```
           render_as="png",
           nudge_height = 0.1,
           nudge_y = 0,
           point_colours = results4$MyColour,
           file_path=outfile)



# Multiple IV analysis: Longitudinal sample
# ----------------------------------------

dataYPL$AgeP1           <- scale(dataYPL$Age)
dataYPL$AgeP2           <- scale((dataYPL$Age)^2)
dataYPL$AgeP3           <- scale((dataYPL$Age)^3)
dataYPL$AgeP4           <- scale((dataYPL$Age)^4)

myMod3                   <- summary(plm(avMSE ~ SLP115:Age + TO103:Age + RB103:Age + AgeP1 + AgeP2 + AgeP3  |
                                    bedtime_var42_81 + DogOwner + CatOwner + BirthOrder + scoreTOSpgs + scoreEDUpgs + scoreSLPpgs
+
                                    Age:bedtime_var42_81 + Age:DogOwner + Age:CatOwner + Age:BirthOrder + Age:scoreTOSpgs +
Age:scoreEDUpgs + Age:scoreSLPpgs + AgeP1 + AgeP2 + AgeP3,
                                    data = dataYPL,
                                    index = c("cidB3675","Visit"), model = "random", random.method = "nerlove", inst.method =
"bvk"))

myMod3Adj                <- summary(plm(avMSE ~ SLP115:Age + TO103:Age + RB103:Age + AgeP1 + AgeP2 + AgeP3 + Sex + TDIQ + mat_age
+ PC1 + PC2 + PC3 |
                                    bedtime_var42_81 + DogOwner + CatOwner + BirthOrder + scoreTOSpgs + scoreEDUpgs + scoreSLPpgs
+
                                    Age:bedtime_var42_81 + Age:DogOwner + Age:CatOwner + Age:BirthOrder + Age:scoreTOSpgs +
Age:scoreEDUpgs + Age:scoreSLPpgs + AgeP1 + AgeP2 + AgeP3 + Sex + TDIQ + mat_age + PC1 + PC2 + PC3,
                                    data = dataYPL,
                                    index = c("cidB3675","Visit"), model = "random", random.method = "nerlove", inst.method =
"bvk"))

exposure_list            <- c("SLP115","TO103","RB103")
num_exposures            <- length(exposure_list)
ivs                      <- "bedtime_var42_81 + DogOwner + CatOwner + BirthOrder + scoreTOSpgs + scoreEDUpgs + scoreSLPpgs +
                             Age:bedtime_var42_81 + Age:DogOwner + Age:CatOwner + Age:BirthOrder + Age:scoreTOSpgs +
Age:scoreEDUpgs + Age:scoreSLPpgs"

results                  <- as.data.frame(matrix(ncol=11, nrow=(4*num_exposures)))
names(results)           <-
c("Outcome","Exposure","IV","BETA_INT","SE_INT","LCI_INT","UCI_INT","P_INT","N","Adjusted","Combined")
```

```
myrow                   <- 1
for(e in 1:num_exposures){
  formExposure          <- exposure_list[e]
  formNonAdj            <- as.formula(paste0("avMSE ~ ",formExposure,":Age + AgeP1 + AgeP2 + AgeP3 | ", ivs, " + poly(Age,3)"))
  formAdj               <- as.formula(paste0("avMSE ~ ",formExposure,":Age + AgeP1 + AgeP2 + AgeP3 + Sex + TDIQ + mat_age + PC1
+ PC2 + PC3 | ", ivs, " + AgeP1 + AgeP2 + AgeP3 + Sex + TDIQ + mat_age + PC1 + PC2 + PC3"))
  myMod                 <- summary(plm(formNonAdj, data = dataYPL, index = c("cidB3675","Visit"), model = "random",
random.method = "nerlove", inst.method = "bvk"))
  myModAdj              <- summary(plm(formAdj,    data = dataYPL, index = c("cidB3675","Visit"), model = "random",
random.method = "nerlove", inst.method = "bvk"))
  outrow                <- grep(formExposure,row.names(myMod$coefficients))
  results[myrow,1]      <- "avMSE"
  results[myrow,2]      <- formExposure
  results[myrow,3]      <- "All seven IVs"
  results[myrow,4]      <- myMod$coefficients[outrow,1]
  results[myrow,5]      <- myMod$coefficients[outrow,2]
  results[myrow,6]      <- myMod$coefficients[outrow,1] - (1.96*myMod$coefficients[outrow,2])
  results[myrow,7]      <- myMod$coefficients[outrow,1] + (1.96*myMod$coefficients[outrow,2])
  results[myrow,8]      <- myMod$coefficients[outrow,4]
  results[myrow,9]      <- 3086
  results[myrow,10]     <- 0
  results[myrow,11]     <- 0
  myrow                 <- myrow + 1
  outrow                <- grep(formExposure,row.names(myModAdj$coefficients))
  results[myrow,1]      <- "avMSE"
  results[myrow,2]      <- formExposure
  results[myrow,3]      <- "All seven IVs"
  results[myrow,4]      <- myModAdj$coefficients[outrow,1]
  results[myrow,5]      <- myModAdj$coefficients[outrow,2]
  results[myrow,6]      <- myModAdj$coefficients[outrow,1] - (1.96*myModAdj$coefficients[outrow,2])
  results[myrow,7]      <- myModAdj$coefficients[outrow,1] + (1.96*myModAdj$coefficients[outrow,2])
  results[myrow,8]      <- myModAdj$coefficients[outrow,4]
  results[myrow,9]      <- 3086
  results[myrow,10]     <- 1
  results[myrow,11]     <- 0
  myrow                 <- myrow + 1

  outrow                <- grep(formExposure,row.names(myMod3$coefficients))
  results[myrow,1]      <- "avMSE"
  results[myrow,2]      <- formExposure
  results[myrow,3]      <- "All seven IVs"
  results[myrow,4]      <- myMod3$coefficients[outrow,1]
  results[myrow,5]      <- myMod3$coefficients[outrow,2]
```

```
  results[myrow,6]       <- myMod3$coefficients[outrow,1] - (1.96*myMod3$coefficients[outrow,2])
  results[myrow,7]       <- myMod3$coefficients[outrow,1] + (1.96*myMod3$coefficients[outrow,2])
  results[myrow,8]       <- myMod3$coefficients[outrow,4]
  results[myrow,9]       <- 3086
  results[myrow,10]      <- 0
  results[myrow,11]      <- 1
  myrow                  <- myrow + 1
  outrow                 <- grep(formExposure,row.names(myMod3Adj$coefficients))
  results[myrow,1]       <- "avMSE"
  results[myrow,2]       <- formExposure
  results[myrow,3]       <- "All seven IVs"
  results[myrow,4]       <- myMod3Adj$coefficients[outrow,1]
  results[myrow,5]       <- myMod3Adj$coefficients[outrow,2]
  results[myrow,6]       <- myMod3Adj$coefficients[outrow,1] - (1.96*myMod3Adj$coefficients[outrow,2])
  results[myrow,7]       <- myMod3Adj$coefficients[outrow,1] + (1.96*myMod3Adj$coefficients[outrow,2])
  results[myrow,8]       <- myMod3Adj$coefficients[outrow,4]
  results[myrow,9]       <- 3086
  results[myrow,10]      <- 1
  results[myrow,11]      <- 1
  myrow                  <- myrow + 1
}
results

results2                 <- results[which(results$Adjusted==1),]
combined_iv_long         <- results2

myrow                    <- 1
results3                 <- results2[0,]
for (n in 1:num_exposures){
  myexposure                            <- exposure_list[n]
  myrows                                <-
results2[which(results2$Exposure==myexposure),c("Outcome","N","BETA_INT","LCI_INT","UCI_INT","Combined")]
  myextra                               <- nrow(myrows)
  results3[myrow,]                      <- NA
  results3$Exposure[myrow]              <- myexposure
  results3[(myrow + 1):(myrow + myextra),c("Outcome","N","BETA_INT","LCI_INT","UCI_INT","Combined")] <- myrows
  myrow                                 <- myrow + myextra + 1
  }

results3$Exposure            <- as.character(results3$Exposure)
results3$Exposure            <- c("Sleep duration at age 9.5 years",NA,NA,"Time outdoors at age 8.5 years",NA,NA,"Time reading at
age 8.5 years",NA,NA)
results3$Combined            <- as.character(results3$Combined)
```

```
results3$Combined             <- c(NA, "Sleep duration", "Sleep, outdoors and reading", NA, "Time outdoors", "Sleep, outdoors and
reading", NA, "Time reading", "Sleep, outdoors and reading")
results4                      <- results3
names(results4)[names(results4) == "Exposure"]    <- "Activity (hours per day)"
names(results4)[names(results4) == "Combined"]    <- "Risk factors in model"

outcsv                        <- paste0(mydir,"TableS4_2024-07-17.csv")
write.csv(results, file=outcsv, row.names=FALSE)

# Combined plot for longitudinal observation and IV analyses
# ---------------------------------------------------------

combined_obs_long$IV      <- as.character("-")
combined_long             <- rbind(combined_obs_long, combined_iv_long)

myrow                     <- 1
results3                  <- combined_long[0,]
for (n in 1:num_exposures){
  myexposure                                <- exposure_list[n]
  myrows                                    <-
combined_long[which(combined_long$Exposure==myexposure),c("Exposure","BETA_INT","LCI_INT","UCI_INT","Combined")]
  myextra                                   <- nrow(myrows)
  results3[myrow,]                          <- NA
  results3$Exposure[myrow]                  <- myexposure
  results3[(myrow + 1):(myrow + myextra),c("Exposure","BETA_INT","LCI_INT","UCI_INT","Combined")] <- myrows
  myrow                                     <- myrow + myextra + 1
  }

results3$Exposure             <- as.character(results3$Exposure)
results3$Exposure             <- c("Sleep duration at age 9.5 years",NA,NA,NA,NA,
                                   "Time outdoors at age 8.5 years",NA,NA,NA,NA,
                                   "Time reading at age 8.5 years",NA,NA,NA,NA)
results3$Combined             <- as.character(results3$Combined)
results3$Combined             <- c(NA, "Sleep duration", "Sleep, outdoors and reading", "Sleep duration", "Sleep, outdoors and
reading",
                                   NA, "Time outdoors", "Sleep, outdoors and reading",  "Time outdoors", "Sleep, outdoors and
reading",
                                   NA, "Time reading", "Sleep, outdoors and reading", "Time reading", "Sleep, outdoors and
reading")
results4                      <- results3
results4$Analysis             <- rep(c("","Observational","Observational","IV","IV"),3)
results4$MyColour             <- rep(c("","dark red","dark red","blue","blue"),3)
names(results4)[names(results4) == "Exposure"]    <- "Risk factor (hours per day)"
names(results4)[names(results4) == "Combined"]    <- "Risk factors in model"
```

```
outfile                      <- paste0(mydir,"Figure3_2024-07-17.png")

forester(left_side_data = results4[,c("Risk factor (hours per day)","Analysis","Risk factors in model")],
           estimate_col_name="Refractive error (D)(95% CI)",
           estimate = results4$BETA,
           ci_low = results4$LCI,
           ci_high = results4$UCI,
           estimate_precision = 2,
           display = TRUE,
           xlim = c(-0.15, 0.15),
           null_line_at = 0,
           font_family = "sans",
           render_as="png",
           nudge_height = 0.1,
           nudge_y = 0,
           point_colours = results4$MyColour,
           file_path=outfile)


###########################################################################################################################

# Observational associations between each IV and sleep duration
# ---------------------------------------------------------------

exposure_list            <- c("CatOwner","DogOwner","BirthOrder","bedtime_var42_81","scoreTOSpgs","scoreEDUpgs","scoreSLPpgs")
num_exposures            <- length(exposure_list)
outcome_list             <- c("SLP42","SLP57","SLP69","SLP81","SLP115","SLP140")
num_outcomes             <- length(outcome_list)

results                  <- as.data.frame(matrix(nrow=1,ncol=8))
names(results)           <- c("Outcome","Exposure","BETA","SE","LCI","UCI","P","N")
myrow                    <- 1
for(e in 1:num_exposures){
  myExposure             <- as.character(exposure_list[e])
  dataYP$myTrait         <- dataYP[,myExposure]
for(f in 1:num_outcomes){
  myOutcome              <- as.character(outcome_list[f])
  dataYP$myOut           <- dataYP[,myOutcome]
  myMod                  <- summary(lm(myOut ~ myTrait, data=dataYP))
  results[myrow,1]       <- myOutcome
  results[myrow,2]       <- myExposure
  results[myrow,3]       <- myMod$coefficients[2,1]
  results[myrow,4]       <- myMod$coefficients[2,2]
```

```
  results[myrow,5]       <- myMod$coefficients[2,1] - (1.96*myMod$coefficients[2,2])
  results[myrow,6]       <- myMod$coefficients[2,1] + (1.96*myMod$coefficients[2,2])
  results[myrow,7]       <- myMod$coefficients[2,4]
  results[myrow,8]       <- myMod$df[1] + myMod$df[2]
  myrow                  <- myrow + 1
}
}
results

results$Exposure         <- as.factor(results$Exposure)
num_exps                 <- length(levels(results$Exposure))
myrow                    <- 1
results2                 <- results[0,]
for (n in 1:num_exps){
  myexposure                             <- levels(results$Exposure)[n]
  myrows                                 <- results[which(results$Exposure==myexposure),c("Outcome","N","BETA","LCI","UCI")]
  myextra                                <- nrow(myrows)
  results2[myrow,]                       <- NA
  results2$Exposure[myrow]               <- myexposure
  results2[(myrow + 1):(myrow + myextra),c("Outcome","N","BETA","LCI","UCI")] <- myrows
  myrow                                  <- myrow + myextra + 1
  }

results2$Outcome          <- as.factor(results2$Outcome)
levels(results2$Outcome)  <- list("9.5"="SLP115","11.5"="SLP140","3.5"="SLP42","5.0"="SLP57", "6.0"="SLP69","7.0"="SLP81")
results2$Outcome          <- ordered(results2$Outcome, levels=c("3.5","5.0","6.0","7.0","9.5","11.5"))

results2$SelfReport       <- ifelse(right(results2$Outcome, n=2)=="SP",1,0)
results2$SelfReport       <- as.factor(results2$SelfReport)
levels(results2$SelfReport)  <- c("Parent-reported time reading", "Self-reported time reading")

levels(results2$Exposure)  <- list("Bedtime variation"="bedtime_var42_81", "Birth order"="BirthOrder",
                                   "Cat owner (parent-reported)"="CatOwner", "Dog owner (parent-reported)"="DogOwner",
                                   "PGS for EduYears"="scoreEDUpgs", "PGS for sleep duration"="scoreSLPpgs", "PGS for time
outdoors"="scoreTOSpgs")

names(results2)[names(results2) == "Exposure"]    <- "Variable"
names(results2)[names(results2) == "Outcome"]     <- "Age (years)"

outfile                   <- paste0(mydir,"FigureS6_2024-05-05.png")

results3                  <- results2[c(1,6:49),] # remove results for sleep duraion at ages used to create Bedtime_variation
variable
```

```
theme_set(theme_classic())
forester(left_side_data = results3[,c("Variable","Age (years)","N")],
           estimate_col_name="Sleep duration in hours per day (95% CI)",
           estimate = results3$BETA,
           ci_low = results3$LCI,
           ci_high = results3$UCI,
           estimate_precision = 2,
           display = TRUE,
           xlim = c(-0.25, 0.25),
           null_line_at = 0,
           font_family = "sans",
           render_as="png",
           file_path=outfile)

# Observational associations between each IV and  time outdoors
# -----------------------------------------------------------

exposure_list             <- c("CatOwner","DogOwner","BirthOrder","bedtime_var42_81","scoreTOSpgs","scoreEDUpgs","scoreSLPpgs")
num_exposures             <- length(exposure_list)
outcome_list              <- c("TO38","TO54","TO65","TO77","TO103","TO167SP")
num_outcomes              <- length(outcome_list)

results                   <- as.data.frame(matrix(nrow=1,ncol=8))
names(results)            <- c("Outcome","Exposure","BETA","SE","LCI","UCI","P","N")
myrow                     <- 1
for(e in 1:num_exposures){
  myExposure              <- as.character(exposure_list[e])
  dataYP$myTrait          <- dataYP[,myExposure]
for(f in 1:num_outcomes){
  myOutcome               <- as.character(outcome_list[f])
  dataYP$myOut            <- dataYP[,myOutcome]
  myMod                   <- summary(lm(myOut ~ myTrait, data=dataYP))
  results[myrow,1]        <- myOutcome
  results[myrow,2]        <- myExposure
  results[myrow,3]        <- myMod$coefficients[2,1]
  results[myrow,4]        <- myMod$coefficients[2,2]
  results[myrow,5]        <- myMod$coefficients[2,1] - (1.96*myMod$coefficients[2,2])
  results[myrow,6]        <- myMod$coefficients[2,1] + (1.96*myMod$coefficients[2,2])
  results[myrow,7]        <- myMod$coefficients[2,4]
  results[myrow,8]        <- myMod$df[1] + myMod$df[2]
  myrow                   <- myrow + 1
}
}
results
```

```
results$Exposure         <- as.factor(results$Exposure)
num_exps                 <- length(levels(results$Exposure))
myrow                    <- 1
results2                 <- results[0,]
for (n in 1:num_exps){
  myexposure                             <- levels(results$Exposure)[n]
  myrows                                 <- results[which(results$Exposure==myexposure),c("Outcome","N","BETA","LCI","UCI")]
  myextra                                <- nrow(myrows)
  results2[myrow,]                       <- NA
  results2$Exposure[myrow]               <- myexposure
  results2[(myrow + 1):(myrow + myextra),c("Outcome","N","BETA","LCI","UCI")] <- myrows
  myrow                                  <- myrow + myextra + 1
  }

results2$Outcome          <- as.factor(results2$Outcome)
levels(results2$Outcome)  <- list("8.5"="TO103",  "14.0"="TO167SP", "3.0"="TO38", "4.5"="TO54",  "5.5"="TO65",  "6.5"="TO77")
results2$Outcome          <- ordered(results2$Outcome, levels=c("3.0","4.5","5.5","6.5","8.5","14.0"))
results2$SelfReport       <- ifelse(right(results2$Outcome, n=2)=="SP",1,0)
results2$SelfReport       <- as.factor(results2$SelfReport)
levels(results2$SelfReport)  <- c("Parent-reported time outdoors", "Self-reported time outdoors")

levels(results2$Exposure)  <- list("Bedtime_variation"="bedtime_var42_81", "Birth order"="BirthOrder",
                                   "Cat owner (parent-reported)"="CatOwner", "Dog owner (parent-reported)"="DogOwner",
                                   "PGS for EduYears"="scoreEDUpgs", "PGS for sleep duration"="scoreSLPpgs", "PGS for time
outdoors"="scoreTOSpgs")

names(results2)[names(results2) == "Exposure"]    <- "Variable"
names(results2)[names(results2) == "Outcome"]     <- "Age (years)"

outfile                   <- paste0(mydir,"FigureS7_2024-05-05.png")

theme_set(theme_classic())
forester(left_side_data = results2[,c("Variable","Age (years)","N")],
           estimate_col_name="Time outdoors in hours per day (95% CI)",
           estimate = results2$BETA,
           ci_low = results2$LCI,
           ci_high = results2$UCI,
           estimate_precision = 2,
           display = TRUE,
           xlim = c(-0.25, 0.25),
           null_line_at = 0,
           font_family = "sans",
           render_as="png",
```

```
          file_path=outfile)


# Observational associations between each IV and  time reading
# -------------------------------------------------------------

exposure_list             <- c("CatOwner","DogOwner","BirthOrder","bedtime_var42_81","scoreTOSpgs","scoreEDUpgs","scoreSLPpgs")
num_exposures             <- length(exposure_list)
outcome_list              <- c("RB54","RB65","RB77","RB103","RB167SP")
num_outcomes              <- length(outcome_list)

results                   <- as.data.frame(matrix(nrow=1,ncol=8))
names(results)            <- c("Outcome","Exposure","BETA","SE","LCI","UCI","P","N")
myrow                     <- 1
for(e in 1:num_exposures){
  myExposure              <- as.character(exposure_list[e])
  dataYP$myTrait          <- dataYP[,myExposure]
for(f in 1:num_outcomes){
  myOutcome               <- as.character(outcome_list[f])
  dataYP$myOut            <- dataYP[,myOutcome]
  myMod                   <- summary(lm(myOut ~ myTrait, data=dataYP))
  results[myrow,1]        <- myOutcome
  results[myrow,2]        <- myExposure
  results[myrow,3]        <- myMod$coefficients[2,1]
  results[myrow,4]        <- myMod$coefficients[2,2]
  results[myrow,5]        <- myMod$coefficients[2,1] - (1.96*myMod$coefficients[2,2])
  results[myrow,6]        <- myMod$coefficients[2,1] + (1.96*myMod$coefficients[2,2])
  results[myrow,7]        <- myMod$coefficients[2,4]
  results[myrow,8]        <- myMod$df[1] + myMod$df[2]
  myrow                   <- myrow + 1
}
}
results

results$Exposure          <- as.factor(results$Exposure)
num_exps                  <- length(levels(results$Exposure))
myrow                     <- 1
results2                  <- results[0,]
for (n in 1:num_exps){
  myexposure                              <- levels(results$Exposure)[n]
  myrows                                  <- results[which(results$Exposure==myexposure),c("Outcome","N","BETA","LCI","UCI")]
  myextra                                 <- nrow(myrows)
  results2[myrow,]                        <- NA
  results2$Exposure[myrow]                <- myexposure
```

```
  results2[(myrow + 1):(myrow + myextra),c("Outcome","N","BETA","LCI","UCI")] <- myrows
  myrow                                   <- myrow + myextra + 1
  }

results2$Outcome           <- as.factor(results2$Outcome)
levels(results2$Outcome)  <- list("8.5"="RB103",  "14.0"="RB167SP",  "4.5"="RB54",  "5.5"="RB65",  "6.5"="RB77")
results2$Outcome           <- ordered(results2$Outcome, levels=c("4.5","5.5","6.5","8.5","14.0"))
results2$SelfReport        <- ifelse(right(results2$Outcome, n=2)=="SP",1,0)
results2$SelfReport        <- as.factor(results2$SelfReport)
levels(results2$SelfReport)  <- c("Parent-reported time reading", "Self-reported time reading")

levels(results2$Exposure)  <- list("Bedtime_variation"="bedtime_var42_81", "Birth order"="BirthOrder",
                                   "Cat owner (parent-reported)"="CatOwner", "Dog owner (parent-reported)"="DogOwner",
                                   "PGS for EduYears"="scoreEDUpgs", "PGS for sleep duration"="scoreSLPpgs", "PGS for time
outdoors"="scoreTOSpgs")

names(results2)[names(results2) == "Exposure"]    <- "Variable"
names(results2)[names(results2) == "Outcome"]     <- "Age (years)"

outfile                   <- paste0(mydir,"FigureS8_2024-05-05.png")

theme_set(theme_classic())
forester(left_side_data = results2[,c("Variable","Age (years)","N")],
           estimate_col_name="Time reading in hours per day (95% CI)",
           estimate = results2$BETA,
           ci_low = results2$LCI,
           ci_high = results2$UCI,
           estimate_precision = 2,
           display = TRUE,
           xlim = c(-0.25, 0.25),
           null_line_at = 0,
           font_family = "sans",
           render_as="png",
           file_path=outfile)

###############################################################################################################################

# IV analysis: Each of seven variables as an IV for sleep duration
# ------------------------------------------------------------------

formOut                  <- "Yr15_avMSE"
iv_list                  <- c("CatOwner","DogOwner","BirthOrder","bedtime_var42_81","scoreTOSpgs","scoreEDUpgs","scoreSLPpgs")
exposure_list            <- c("SLP115")
cov_list                 <- c("Sex","TDIQ","mat_age","PC1","PC2","PC3")
```

```
num_ivs                  <- length(iv_list)
num_exposures            <- length(exposure_list)

results                  <- as.data.frame(matrix(nrow=1,ncol=11))
names(results)           <- c("Outcome","Exposure","IV","F","BETA","SE","LCI","UCI","P","N","Adjusted")
myrow                    <- 1
for(e in 1:num_exposures){
  formExposure           <- as.character(exposure_list[e])
for(f in 1:num_ivs){
  formIV                 <- as.character(iv_list[f])
  formNonAdj             <- as.formula(paste(formOut,"~ 1 |",formExposure,"~",formIV))
  formAdj                <- as.formula(paste(formOut,"~", paste(cov_list, collapse="+"),"|",formExposure,"~",formIV))
  myMod                  <- summary(feols(formNonAdj, data = dataYPW, notes=FALSE))
  myModAdj               <- summary(feols(formAdj, data = dataYPW, notes=FALSE))
  results[myrow,1]       <- formOut
  results[myrow,2]       <- formExposure
  results[myrow,3]       <- formIV
  results[myrow,4]       <- fitstat(myMod,type="ivf",simplify=TRUE)$stat
  results[myrow,5]       <- myMod$coeftable[2,1]
  results[myrow,6]       <- myMod$coeftable[2,2]
  results[myrow,7]       <- myMod$coeftable[2,1] - (1.96*myMod$coeftable[2,2])
  results[myrow,8]       <- myMod$coeftable[2,1] + (1.96*myMod$coeftable[2,2])
  results[myrow,9]       <- myMod$coeftable[2,4]
  results[myrow,10]      <- myMod$nobs
  results[myrow,11]      <- 0
  myrow                  <- myrow + 1
  results[myrow,1]       <- formOut
  results[myrow,2]       <- formExposure
  results[myrow,3]       <- formIV
  results[myrow,4]       <- fitstat(myModAdj,type="ivf",simplify=TRUE)$stat
  results[myrow,5]       <- myModAdj$coeftable[2,1]
  results[myrow,6]       <- myModAdj$coeftable[2,2]
  results[myrow,7]       <- myModAdj$coeftable[2,1] - (1.96*myModAdj$coeftable[2,2])
  results[myrow,8]       <- myModAdj$coeftable[2,1] + (1.96*myModAdj$coeftable[2,2])
  results[myrow,9]       <- myModAdj$coeftable[2,4]
  results[myrow,10]      <- myModAdj$nobs
  results[myrow,11]      <- 1
  myrow                  <- myrow + 1
}
}

results2                  <- results[order(results$Adjusted),]
myrow                     <- 1
results3                  <- results[0,]
```

```
for (n in 0:1){
  myrows                              <-
results2[which(results2$Adjusted==n),c("Outcome","Exposure","IV","F","BETA","LCI","UCI","N")]
  myextra                             <- nrow(myrows)
  results3[myrow,]                    <- NA
  results3$Adjusted[myrow]            <- n
  results3[(myrow + 1):(myrow + myextra),c("Outcome","Exposure","IV","F","BETA","LCI","UCI","N")] <- myrows
  myrow                               <- myrow + myextra + 1
  }

results3$Adjusted         <- as.factor(results3$Adjusted)
levels(results3$Adjusted) <- c("Unadjusted analysis","Adjusted analysis")
results3$IV               <- as.factor(results3$IV)
levels(results3$IV)       <- list("Bedtime_variation"="bedtime_var42_81", "Birth order"="BirthOrder",
                                  "Cat owner (parent-reported)"="CatOwner", "Dog owner (parent-reported)"="DogOwner",
                                  "PGS for EduYears"="scoreEDUpgs", "PGS for sleep duration"="scoreSLPpgs", "PGS for time
outdoors"="scoreTOSpgs")

results3$F                            <- sprintf("%.2f", results3$F)
results3$F                            <- ifelse(results3$F=="NA","",results3$F)
names(results3)[names(results3) == "F"]    <- "F-stat (1st stage)"

outfile                   <- paste0(mydir,"FigureS9_2024-05-05.png")

theme_set(theme_classic())
forester(left_side_data = results3[,c("Adjusted","IV","N","F-stat (1st stage)")],
           estimate_col_name="Refractive error in diopters (95% CI)",
           estimate = results3$BETA,
           ci_low = results3$LCI,
           ci_high = results3$UCI,
           estimate_precision = 2,
           display = TRUE,
           xlim = c(-5, 5),
           null_line_at = 0,
           font_family = "sans",
           render_as="png",
           file_path=outfile)


# IV analysis: Each of seven variables as an IV for time outdoors
# ------------------------------------------------------------------

formOut                  <- "Yr15_avMSE"
iv_list                  <- c("CatOwner","DogOwner","BirthOrder","bedtime_var42_81","scoreTOSpgs","scoreEDUpgs","scoreSLPpgs")
```

```
exposure_list             <- c("TO103")
cov_list                  <- c("Sex","TDIQ","mat_age","PC1","PC2","PC3")
num_ivs                   <- length(iv_list)
num_exposures             <- length(exposure_list)

results                   <- as.data.frame(matrix(nrow=1,ncol=11))
names(results)            <- c("Outcome","Exposure","IV","F","BETA","SE","LCI","UCI","P","N","Adjusted")
myrow                     <- 1
for(e in 1:num_exposures){
  formExposure            <- as.character(exposure_list[e])
for(f in 1:num_ivs){
  formIV                  <- as.character(iv_list[f])
  formNonAdj              <- as.formula(paste(formOut,"~ 1 |",formExposure,"~",formIV))
  formAdj                 <- as.formula(paste(formOut,"~", paste(cov_list, collapse="+"),"|",formExposure,"~",formIV))
  myMod                   <- summary(feols(formNonAdj, data = dataYPW, notes=FALSE))
  myModAdj                <- summary(feols(formAdj, data = dataYPW, notes=FALSE))
  results[myrow,1]        <- formOut
  results[myrow,2]        <- formExposure
  results[myrow,3]        <- formIV
  results[myrow,4]        <- fitstat(myMod,type="ivf",simplify=TRUE)$stat
  results[myrow,5]        <- myMod$coeftable[2,1]
  results[myrow,6]        <- myMod$coeftable[2,2]
  results[myrow,7]        <- myMod$coeftable[2,1] - (1.96*myMod$coeftable[2,2])
  results[myrow,8]        <- myMod$coeftable[2,1] + (1.96*myMod$coeftable[2,2])
  results[myrow,9]        <- myMod$coeftable[2,4]
  results[myrow,10]       <- myMod$nobs
  results[myrow,11]       <- 0
  myrow                   <- myrow + 1
  results[myrow,1]        <- formOut
  results[myrow,2]        <- formExposure
  results[myrow,3]        <- formIV
  results[myrow,4]        <- fitstat(myModAdj,type="ivf",simplify=TRUE)$stat
  results[myrow,5]        <- myModAdj$coeftable[2,1]
  results[myrow,6]        <- myModAdj$coeftable[2,2]
  results[myrow,7]        <- myModAdj$coeftable[2,1] - (1.96*myModAdj$coeftable[2,2])
  results[myrow,8]        <- myModAdj$coeftable[2,1] + (1.96*myModAdj$coeftable[2,2])
  results[myrow,9]        <- myModAdj$coeftable[2,4]
  results[myrow,10]       <- myModAdj$nobs
  results[myrow,11]       <- 1
  myrow                   <- myrow + 1
}
}

results2                   <- results[order(results$Adjusted),]
```

```
myrow                     <- 1
results3                  <- results[0,]
for (n in 0:1){
  myrows                                <-
results2[which(results2$Adjusted==n),c("Outcome","Exposure","IV","F","BETA","LCI","UCI","N")]
  myextra                               <- nrow(myrows)
  results3[myrow,]                      <- NA
  results3$Adjusted[myrow]              <- n
  results3[(myrow + 1):(myrow + myextra),c("Outcome","Exposure","IV","F","BETA","LCI","UCI","N")] <- myrows
  myrow                                 <- myrow + myextra + 1
  }

results3$Adjusted         <- as.factor(results3$Adjusted)
levels(results3$Adjusted) <- c("Unadjusted analysis","Adjusted analysis")
results3$IV               <- as.factor(results3$IV)
levels(results3$IV)       <- list("Bedtime_variation"="bedtime_var42_81", "Birth order"="BirthOrder",
                                   "Cat owner (parent-reported)"="CatOwner", "Dog owner (parent-reported)"="DogOwner",
                                   "PGS for EduYears"="scoreEDUpgs", "PGS for sleep duration"="scoreSLPpgs", "PGS for time
outdoors"="scoreTOSpgs")

results3$F                              <- sprintf("%.2f", results3$F)
results3$F                              <- ifelse(results3$F=="NA","",results3$F)
names(results3)[names(results3) == "F"]    <- "F-stat (1st stage)"

outfile                   <- paste0(mydir,"FigureS10_2024-05-05.png")

theme_set(theme_classic())
forester(left_side_data = results3[,c("Adjusted","IV","N","F-stat (1st stage)")],
           estimate_col_name="Refractive error in diopters (95% CI)",
           estimate = results3$BETA,
           ci_low = results3$LCI,
           ci_high = results3$UCI,
           estimate_precision = 2,
           display = TRUE,
           xlim = c(-5, 5),
           null_line_at = 0,
           font_family = "sans",
           render_as="png",
           file_path=outfile)

# IV analysis: Each of seven variables as an IV for time reading
# -----------------------------------------------------------------

formOut                  <- "Yr15_avMSE"
```

```
iv_list                  <- c("CatOwner","DogOwner","BirthOrder","bedtime_var42_81","scoreTOSpgs","scoreEDUpgs","scoreSLPpgs")
exposure_list            <- c("RB103")
cov_list                 <- c("Sex","TDIQ","mat_age","PC1","PC2","PC3")
num_ivs                  <- length(iv_list)
num_exposures            <- length(exposure_list)

results                  <- as.data.frame(matrix(nrow=1,ncol=11))
names(results)           <- c("Outcome","Exposure","IV","F","BETA","SE","LCI","UCI","P","N","Adjusted")
myrow                    <- 1
for(e in 1:num_exposures){
  formExposure           <- as.character(exposure_list[e])
for(f in 1:num_ivs){
  formIV                 <- as.character(iv_list[f])
  formNonAdj             <- as.formula(paste(formOut,"~ 1 |",formExposure,"~",formIV))
  formAdj                <- as.formula(paste(formOut,"~", paste(cov_list, collapse="+"),"|",formExposure,"~",formIV))
  myMod                  <- summary(feols(formNonAdj, data = dataYPW, notes=FALSE))
  myModAdj               <- summary(feols(formAdj, data = dataYPW, notes=FALSE))
  results[myrow,1]       <- formOut
  results[myrow,2]       <- formExposure
  results[myrow,3]       <- formIV
  results[myrow,4]       <- fitstat(myMod,type="ivf",simplify=TRUE)$stat
  results[myrow,5]       <- myMod$coeftable[2,1]
  results[myrow,6]       <- myMod$coeftable[2,2]
  results[myrow,7]       <- myMod$coeftable[2,1] - (1.96*myMod$coeftable[2,2])
  results[myrow,8]       <- myMod$coeftable[2,1] + (1.96*myMod$coeftable[2,2])
  results[myrow,9]       <- myMod$coeftable[2,4]
  results[myrow,10]      <- myMod$nobs
  results[myrow,11]      <- 0
  myrow                  <- myrow + 1
  results[myrow,1]       <- formOut
  results[myrow,2]       <- formExposure
  results[myrow,3]       <- formIV
  results[myrow,4]       <- fitstat(myModAdj,type="ivf",simplify=TRUE)$stat
  results[myrow,5]       <- myModAdj$coeftable[2,1]
  results[myrow,6]       <- myModAdj$coeftable[2,2]
  results[myrow,7]       <- myModAdj$coeftable[2,1] - (1.96*myModAdj$coeftable[2,2])
  results[myrow,8]       <- myModAdj$coeftable[2,1] + (1.96*myModAdj$coeftable[2,2])
  results[myrow,9]       <- myModAdj$coeftable[2,4]
  results[myrow,10]      <- myModAdj$nobs
  results[myrow,11]      <- 1
  myrow                  <- myrow + 1
}
}
```

```
results2                  <- results[order(results$Adjusted),]
myrow                     <- 1
results3                  <- results[0,]
for (n in 0:1){
  myrows                                <-
results2[which(results2$Adjusted==n),c("Outcome","Exposure","IV","F","BETA","LCI","UCI","N")]
  myextra                               <- nrow(myrows)
  results3[myrow,]                      <- NA
  results3$Adjusted[myrow]              <- n
  results3[(myrow + 1):(myrow + myextra),c("Outcome","Exposure","IV","F","BETA","LCI","UCI","N")] <- myrows
  myrow                                 <- myrow + myextra + 1
  }

results3$Adjusted         <- as.factor(results3$Adjusted)
levels(results3$Adjusted) <- c("Unadjusted analysis","Adjusted analysis")
results3$IV               <- as.factor(results3$IV)
levels(results3$IV)       <- list("Bedtime_variation"="bedtime_var42_81", "Birth order"="BirthOrder",
                                  "Cat owner (parent-reported)"="CatOwner", "Dog owner (parent-reported)"="DogOwner",
                                  "PGS for EduYears"="scoreEDUpgs", "PGS for sleep duration"="scoreSLPpgs", "PGS for time
outdoors"="scoreTOSpgs")

results3$F                              <- sprintf("%.2f", results3$F)
results3$F                              <- ifelse(results3$F=="NA","",results3$F)
names(results3)[names(results3) == "F"]    <- "F-stat (1st stage)"

outfile                   <- paste0(mydir,"FigureS11_2024-05-05.png")

theme_set(theme_classic())
forester(left_side_data = results3[,c("Adjusted","IV","N","F-stat (1st stage)")],
           estimate_col_name="Refractive error in diopters (95% CI)",
           estimate = results3$BETA,
           ci_low = results3$LCI,
           ci_high = results3$UCI,
           estimate_precision = 2,
           display = TRUE,
           xlim = c(-5, 5),
           null_line_at = 0,
           font_family = "sans",
           render_as="png",
           file_path=outfile)

##########################################################################################################
```

```
# Top 3 IVs
# =========

# TO  -> DogOwner    + PGS_TO  + PGS_EDU
# RB  -> BirthOrder  + PGS_EDU + PGS_SLP
# SLP -> bedtime_var + PGS_TO  + PGS_EDU

exposure_list            <- c("SLP115","TO103","RB103")
num_exposures            <- length(exposure_list)

TO_3IV_Adj                 <- as.formula("Yr15_avMSE ~ Sex + TDIQ + SLP115 + RB103 + mat_age + PC1 + PC2 + PC3 | TO103 ~
                                                     DogOwner + scoreTOSpgs + scoreEDUpgs")
TO_7IV_Adj                 <- as.formula("Yr15_avMSE ~ Sex + TDIQ + SLP115 + RB103 + mat_age + PC1 + PC2 + PC3 | TO103 ~
                                                     CatOwner + DogOwner + BirthOrder + bedtime_var42_81 + scoreTOSpgs +
scoreEDUpgs + scoreSLPpgs")

RB_3IV_Adj                 <- as.formula("Yr15_avMSE ~ Sex + TDIQ + SLP115 + TO103 + mat_age + PC1 + PC2 + PC3 | RB103 ~
                                                     BirthOrder + scoreEDUpgs + scoreSLPpgs")
RB_7IV_Adj                 <- as.formula("Yr15_avMSE ~ Sex + TDIQ + SLP115 + TO103 + mat_age + PC1 + PC2 + PC3 | RB103 ~
                                                     CatOwner + DogOwner + BirthOrder + bedtime_var42_81 + scoreTOSpgs +
scoreEDUpgs + scoreSLPpgs")

SLP_3IV_Adj                <- as.formula("Yr15_avMSE ~ Sex + TDIQ + TO103 + RB103 + mat_age + PC1 + PC2 + PC3 | SLP115 ~
                                                     bedtime_var42_81 + scoreTOSpgs + scoreEDUpgs")
SLP_7IV_Adj                <- as.formula("Yr15_avMSE ~ Sex + TDIQ + TO103 + RB103 + mat_age + PC1 + PC2 + PC3 | SLP115 ~
                                                     CatOwner + DogOwner + BirthOrder + bedtime_var42_81 + scoreTOSpgs +
scoreEDUpgs + scoreSLPpgs")

myModTO_3IV_Adj            <- summary(feols(TO_3IV_Adj,    data = dataYPW, notes=FALSE))
myModTO_7IV_Adj            <- summary(feols(TO_7IV_Adj,    data = dataYPW, notes=FALSE))
myModRB_3IV_Adj            <- summary(feols(RB_3IV_Adj,    data = dataYPW, notes=FALSE))
myModRB_7IV_Adj            <- summary(feols(RB_7IV_Adj,    data = dataYPW, notes=FALSE))
myModSLP_3IV_Adj           <- summary(feols(SLP_3IV_Adj,   data = dataYPW, notes=FALSE))
myModSLP_7IV_Adj           <- summary(feols(SLP_7IV_Adj,   data = dataYPW, notes=FALSE))

results                  <- as.data.frame(matrix(nrow=6,ncol=10))
names(results)           <- c("Outcome","Exposure","IV","F","BETA","LCI","UCI","P","N","Adjusted")

results[,1]              <- "Yr15_avMSE"
results[,2]              <- c("TO103","TO103","RB103","RB103","SLP115","SLP115")
results[,3]              <- c("Top three IVs","All seven IVs","Top three IVs","All seven IVs","Top three IVs","All seven IVs")
results[,9]              <- myModTO_3IV_Adj$nobs
results[,10]             <- 1
results[1,4]             <- sprintf("%.2f", fitstat(myModTO_3IV_Adj,type="ivf",simplify=TRUE)$stat)
```

```
results[2,4]             <- sprintf("%.2f", fitstat(myModTO_7IV_Adj,type="ivf",simplify=TRUE)$stat)
results[3,4]             <- sprintf("%.2f", fitstat(myModRB_3IV_Adj,type="ivf",simplify=TRUE)$stat)
results[4,4]             <- sprintf("%.2f", fitstat(myModRB_7IV_Adj,type="ivf",simplify=TRUE)$stat)
results[5,4]             <- sprintf("%.2f", fitstat(myModSLP_3IV_Adj,type="ivf",simplify=TRUE)$stat)
results[6,4]             <- sprintf("%.2f", fitstat(myModSLP_7IV_Adj,type="ivf",simplify=TRUE)$stat)

results[1,5]             <- myModTO_3IV_Adj$coeftable[2,1]
results[2,5]             <- myModTO_7IV_Adj$coeftable[2,1]
results[3,5]             <- myModRB_3IV_Adj$coeftable[2,1]
results[4,5]             <- myModRB_7IV_Adj$coeftable[2,1]
results[5,5]             <- myModSLP_3IV_Adj$coeftable[2,1]
results[6,5]             <- myModSLP_7IV_Adj$coeftable[2,1]

results[1,6]             <- myModTO_3IV_Adj$coeftable[2,1] - (1.96*myModTO_3IV_Adj$coeftable[2,2])
results[2,6]             <- myModTO_7IV_Adj$coeftable[2,1] - (1.96*myModTO_7IV_Adj$coeftable[2,2])
results[3,6]             <- myModRB_3IV_Adj$coeftable[2,1] - (1.96*myModRB_3IV_Adj$coeftable[2,2])
results[4,6]             <- myModRB_7IV_Adj$coeftable[2,1] - (1.96*myModRB_7IV_Adj$coeftable[2,2])
results[5,6]             <- myModSLP_3IV_Adj$coeftable[2,1] - (1.96*myModSLP_3IV_Adj$coeftable[2,2])
results[6,6]             <- myModSLP_7IV_Adj$coeftable[2,1] - (1.96*myModSLP_7IV_Adj$coeftable[2,2])

results[1,7]             <- myModTO_3IV_Adj$coeftable[2,1] + (1.96*myModTO_3IV_Adj$coeftable[2,2])
results[2,7]             <- myModTO_7IV_Adj$coeftable[2,1] + (1.96*myModTO_7IV_Adj$coeftable[2,2])
results[3,7]             <- myModRB_3IV_Adj$coeftable[2,1] + (1.96*myModRB_3IV_Adj$coeftable[2,2])
results[4,7]             <- myModRB_7IV_Adj$coeftable[2,1] + (1.96*myModRB_7IV_Adj$coeftable[2,2])
results[5,7]             <- myModSLP_3IV_Adj$coeftable[2,1] + (1.96*myModSLP_3IV_Adj$coeftable[2,2])
results[6,7]             <- myModSLP_7IV_Adj$coeftable[2,1] + (1.96*myModSLP_7IV_Adj$coeftable[2,2])

results[1,8]             <- myModTO_3IV_Adj$coeftable[2,4]
results[2,8]             <- myModTO_7IV_Adj$coeftable[2,4]
results[3,8]             <- myModRB_3IV_Adj$coeftable[2,4]
results[4,8]             <- myModRB_7IV_Adj$coeftable[2,4]
results[5,8]             <- myModSLP_3IV_Adj$coeftable[2,4]
results[6,8]             <- myModSLP_7IV_Adj$coeftable[2,4]

myrow                     <- 1
results3                  <- results[0,]
for (n in 1:num_exposures){
  myexposure                              <- exposure_list[n]
  myrows                                  <- 
results[which(results$Exposure==myexposure),c("Outcome","IV","F","BETA","LCI","UCI")]
  myextra                                 <- nrow(myrows)
  results3[myrow,]                        <- NA
  results3$Exposure[myrow]                <- myexposure
  results3[(myrow + 1):(myrow + myextra),c("Outcome","IV","F","BETA","LCI","UCI")] <- myrows
```

```
  myrow                                  <- myrow + myextra + 1
  }

results3$Exposure                        <- as.character(results3$Exposure)
results3$Exposure                        <- c("Sleep duration at age 9.5 years",NA,NA,"Time outdoors at age 8.5 
years",NA,NA,"Time reading at age 8.5 years",NA,NA)
results3$IV                              <- as.factor(results3$IV)
results3$F                               <- ifelse(results3$F=="NA","",results3$F)
names(results3)[names(results3) == "F"]    <- "F-stat (1st stage)"


outfile                  <- paste(mydir,"Top3IVs_2024-07-22.png")

forester(left_side_data = results3[,c("Exposure","IV","F-stat (1st stage)")],
           estimate_col_name="Refractive error in diopters (95% CI)",
           estimate = results3$BETA,
           ci_low = results3$LCI,
           ci_high = results3$UCI,
           estimate_precision = 2,
           display = TRUE,
           xlim = c(-2.5, 2.5),
           null_line_at = 0,
           font_family = "sans",
           render_as="png",
           nudge_height = 0.1,
           nudge_y = 0,
           file_path=outfile)
```

Supplementary Note S3. Observational associations of sleep duration, time outdoors and time reading at early ages with the outcome, 'refractive error at age 15 years'.

Analyses were carried out in the full sample. In analyses that adjusted for gender, socioeconomic status and maternal age, sleep duration in infancy had a weak positive association with refractive error at age 15 years, but this relationship waned as children got older (Supplementary Table S1and Supplementary Figure S4). The strongest evidence for an association was at age 3 years-old, when one additional hour asleep per day was associated with a +0.07 D (95% C.I. 0.03 to 0.16, $P = 0.011$) more hyperopic refractive error by the age of 15 years. Compared to sleep duration, time outdoors had a larger, positive association with refractive error at age 15 years, while time reading books had a negative association from the age of 5.5 years that increased in size as children got older (Supplementary Figure S4). The strongest association with time outdoors was at 14 years-old, when one additional hour outdoors per day was associated with a + 0.19 D (95% C.I. 0.09 to 0.29, $P < 0.001$) more hyperopic refractive error by the age of 15 years. The strongest association with time reading books also was at 14 years-old, when one additional hour reading per day was associated with a -0.23 D (95% C.I. -0.16 to -0.30, $P < 0.001$) more myopic refractive error by the age of 15 years. The results of analyses that did not adjust for the effects of gender and socioeconomic status were very similar to the adjusted analyses (Supplementary Table S1).

Supplementary Note S4. Selection of instrumental variables for each myopia risk factor.

For sleep duration, which has been less intensively studied as a potential risk factor for myopia than the other two predictors, there were small but statistically significant associations with several demographic characteristics and behaviors, as detailed in Supplementary Figure S5.

A key criterion for an IV is that it has a robust association with the risk factor of interest (quantified using the 1st stage F-statistic). We considered a total of seven different IVs for our three risk factors of interest. For sleep duration, bedtime variability (between 3.5 and 7 years of age) and a PGS for sleep duration in adulthood were chosen as potential IVs. Dog ownership by the family at any time when child was aged 7-15 years, and a PGS for time outdoors were chosen as potential IVs for time outdoors (with cat ownership by the family when child was aged 7-15 years as a negative control IV). Birth order and a PGS for years spent in education were chosen as potential IVs for time spent reading. Of these seven variables, bedtime variability, the PGS for EduYears, and the PGS for time outdoors were most strongly associated with sleep duration (Supplementary Figure S6). For instance, a one standard deviation increase in bedtime variability was associated with a 0.10 hour (95% C.I. 0.04 to 0.15, $P < 0.001$) reduction in sleep duration at age 9.5 years. The variables most strongly associated with time outdoors at age 8.5 years were dog ownership, bedtime variability, and the PGSs for time outdoors and EduYears (Supplementary Figure S7). For example, dog ownership was associated with an additional 0.07 hours spent outdoors per day (95% C.I. 0.04 to 0.10, $P < 0.001$). Many of the seven variables had complex associations with children's time reading, with reversals in direction across childhood (Supplementary Figure S8). For time reading at age 8.5 years, the most strongly associated variables were birth order and the PGSs for EduYears, time outdoors, and sleep duration. Having an additional older sibling was associated with a reduction in time reading per day of 0.07 hours (95% C.I. -0.09 to -0.05, $P < 0.001$).

In summary, these analyses suggested that bedtime variability was a better IV for sleep duration than a PRS for adult sleep duration. Importantly, the analyses also suggested that most of the seven IVs captured effects relating to more than one risk factor. This latter attribute would invalidate the variables as stand-alone IVs. Instead, this work implied the seven variables were better suited to a multiple-IV framework that assessed the risk of the three putative risk factors simultaneously.

Supplementary Table S1. Observational analysis of risk factors associated with the outcome 'refractive error at age 15 years' in the full sample.

Risk factor	Age (years) when risk factor measured	BETA (D/hour)	SE	95% LCI	95% UCI	*P*	N	Adjusted [a]	Self-reported [b]
Sleep duration	3	0.079	0.026	0.028	0.130	2.56E-03	3504	No	No
	3	0.070	0.028	0.016	0.124	1.13E-02	3292	Yes	No
	4.5	0.060	0.033	-0.004	0.124	6.62E-02	3470	No	No
	4.5	0.045	0.035	-0.023	0.113	1.91E-01	3255	Yes	No
	5.5	0.028	0.033	-0.035	0.092	3.83E-01	3344	No	No
	5.5	0.014	0.034	-0.053	0.081	6.84E-01	3137	Yes	No
	6.5	0.003	0.037	-0.069	0.075	9.27E-01	3293	No	No
	6.5	-0.018	0.039	-0.094	0.058	6.39E-01	3091	Yes	No
	9.5	0.038	0.035	-0.032	0.107	2.88E-01	3549	No	No
	9.5	0.029	0.039	-0.047	0.105	4.59E-01	3196	Yes	No
	11.5	-0.008	0.037	-0.082	0.065	8.22E-01	3359	No	No
	11.5	-0.018	0.041	-0.098	0.062	6.60E-01	3019	Yes	No
Time outdoors	3	0.100	0.053	-0.004	0.204	6.02E-02	3494	No	No
	3	0.085	0.056	-0.026	0.195	1.32E-01	3279	Yes	No
	4.5	0.097	0.048	0.003	0.192	4.42E-02	3412	No	No
	4.5	0.090	0.051	-0.010	0.189	7.65E-02	3203	Yes	No
	5.5	0.034	0.047	-0.058	0.126	4.72E-01	3354	No	No
	5.5	0.011	0.049	-0.085	0.106	8.29E-01	3152	Yes	No
	6.5	0.099	0.056	-0.011	0.209	7.92E-02	3271	No	No
	6.5	0.088	0.059	-0.028	0.203	1.37E-01	3067	Yes	No
	8.5	0.097	0.050	-0.001	0.194	5.26E-02	3446	No	No
	8.5	0.095	0.054	-0.011	0.201	7.91E-02	3112	Yes	No
	14	0.184	0.048	0.089	0.279	1.46E-04	2651	No	Yes
	14	0.186	0.051	0.087	0.285	2.52E-04	2497	Yes	Yes

Supplementary Table S1. Continued…

Risk factor	Age (years) when risk factor measured	BETA (D/hour)	SE	95% LCI	95% UCI	*P*	N	Adjusted [a]	Self-reported [b]
Time reading	4.5	-0.003	0.035	-0.071	0.066	9.40E-01	3422	No	No
	4.5	-0.016	0.036	-0.088	0.055	6.55E-01	3211	Yes	No
	5.5	-0.064	0.035	-0.134	0.005	6.82E-02	3369	No	No
	5.5	-0.082	0.037	-0.155	-0.009	2.87E-02	3161	Yes	No
	6.5	-0.161	0.042	-0.244	-0.078	1.50E-04	3293	No	No
	6.5	-0.181	0.045	-0.270	-0.092	6.77E-05	3087	Yes	No
	8.5	-0.195	0.037	-0.268	-0.123	1.44E-07	3465	No	No
	8.5	-0.201	0.041	-0.281	-0.121	9.14E-07	3131	Yes	No
	14	-0.220	0.034	-0.286	-0.155	5.97E-11	3091	No	Yes
	14	-0.230	0.037	-0.302	-0.158	4.11E-10	2785	Yes	Yes

[a] Adjusted analyses controlled for gender, maternal age and Townsend Deprivation Index (quintile).
[b] Risk factor exposure levels were reported by the mother, except where indicated as self-reported.

Supplementary Table S2. Observational analysis of risk factors associated with the outcome 'refractive error' in the longitudinal sample.

**Risk factor**	Age (years) when risk factor measured	**BETA (Annual change in D per hour)**	**SE**	**95% LCI**	**95% UCI**	***P***	**N**	**Adjusted [a]**	**Combined [b]**
Sleep duration	9.5	0.001	0.002	-0.003	0.004	5.85E-01	3086	No	No
	9.5	0.001	0.002	-0.003	0.004	6.51E-01	3086	Yes	No
	9.5	0.001	0.002	-0.002	0.005	5.23E-01	3086	No	Yes
	9.5	0.001	0.002	-0.003	0.004	6.69E-01	3086	Yes	Yes
Time outdoors	8.5	0.009	0.002	0.004	0.013	5.93E-04	3086	No	No
	8.5	0.008	0.003	0.003	0.013	1.30E-03	3086	Yes	No
	8.5	0.009	0.002	0.004	0.014	3.25E-04	3086	No	Yes
	8.5	0.009	0.003	0.004	0.013	6.05E-04	3086	Yes	Yes
Time reading	8.5	-0.015	0.002	-0.019	-0.011	2.15E-15	3086	No	No
	8.5	-0.015	0.002	-0.019	-0.012	1.34E-15	3086	Yes	No
	8.5	-0.015	0.002	-0.019	-0.012	9.14E-16	3086	No	Yes
	8.5	-0.016	0.002	-0.019	-0.012	5.55E-16	3086	Yes	Yes

[a] Adjusted analyses controlled for gender, maternal age and Townsend Deprivation Index (quintile).
[b] Risk factors were considered either singly (Combined = "No") or with all three included together in the regression model (Combined = "Yes").

Supplementary Table S3. Instrumental variable analysis of risk factors associated with the outcome 'refractive error at age 15 years' in the cross-sectional.

Risk factor	Age (years) when risk factor measured	F-statistic (1st stage)	BETA (D)	SE	95% LCI	95% UCI	*P*	N	Adjusted [a]	Combined [b]
Sleep duration	9.5	9.06	0.370	0.262	-0.142	0.883	1.57E-01	2302	No	No
	9.5	8.41	0.232	0.272	-0.301	0.765	3.93E-01	2302	Yes	No
	9.5	9.06	-0.439	0.399	-1.221	0.343	2.71E-01	2302	No	Yes
	9.5	8.61	-0.384	0.384	-1.136	0.368	3.17E-01	2302	Yes	Yes
Time outdoors	8.5	7.30	1.049	0.424	0.218	1.881	1.34E-02	2302	No	No
	8.5	6.26	0.936	0.453	0.048	1.824	3.90E-02	2302	Yes	No
	8.5	7.30	1.015	0.581	-0.124	2.154	8.09E-02	2302	No	Yes
	8.5	6.06	0.460	0.622	-0.759	1.678	4.60E-01	2302	Yes	Yes
Time reading	8.5	8.48	-0.895	0.298	-1.479	-0.311	2.70E-03	2302	No	No
	8.5	10.31	-0.882	0.274	-1.418	-0.345	1.30E-03	2302	Yes	No
	8.5	8.48	-0.866	0.367	-1.585	-0.146	1.85E-02	2302	No	Yes
	8.5	9.63	-1.017	0.402	-1.805	-0.228	1.16E-02	2302	Yes	Yes

[a] Adjusted analyses controlled for gender, maternal age, Townsend Deprivation Index and the first three genetic ancestry principal components.
[b] Risk factors were considered either singly (Combined = "No") or with all three included together in the regression model (Combined = "Yes").

Supplementary Table S4. Instrumental variable analysis of risk factors associated with the outcome 'refractive error' in the longitudinal sample.

Risk factor	Age (years) when risk factor measured	BETA (Annual change in D per hour)	SE	95% LCI	95% UCI	*P*	N	Adjusted [a]	Combined [b]
Sleep duration	9.5	0.030	0.012	0.006	0.054	1.53E-02	3086	No	No
	9.5	0.031	0.013	0.007	0.056	1.24E-02	3086	Yes	No
	9.5	-0.027	0.019	-0.064	0.009	1.45E-01	3086	No	Yes
	9.5	-0.024	0.019	-0.061	0.012	1.94E-01	3086	Yes	Yes
Time outdoors	8.5	0.068	0.018	0.034	0.103	1.11E-04	3086	No	No
	8.5	0.067	0.018	0.031	0.103	2.58E-04	3086	Yes	No
	8.5	0.062	0.024	0.015	0.109	1.01E-02	3086	No	Yes
	8.5	0.047	0.024	-0.001	0.095	5.51E-02	3086	Yes	Yes
Time reading	8.5	-0.053	0.013	-0.078	-0.028	2.40E-05	3086	No	No
	8.5	-0.055	0.013	-0.080	-0.031	9.98E-06	3086	Yes	No
	8.5	-0.048	0.016	-0.079	-0.017	2.57E-03	3086	No	Yes
	8.5	-0.053	0.017	-0.087	-0.020	1.86E-03	3086	Yes	Yes

[a] Adjusted analyses controlled for gender, maternal age, Townsend Deprivation Index and the first three genetic ancestry principal components.
[b] Risk factors were considered either singly (Combined = "No") or with all three included together in the regression model (Combined = "Yes").

Supplementary Table S5. Pearson correlation matrix for the instrumental variables in the cross-sectional sample.

IV	Bedtime variability	Dog Owner	Cat Owner	Birth Order	PGS for Time outdoors	PGS for EduYears	PGS for Sleep Duration
Bedtime variability	1.000	0.097**	-0.007	0.095**	0.044	-0.068**	0.010
Dog Owner	0.097**	1.000	-0.064**	0.110**	0.080**	-0.076**	-0.021
Cat Owner	-0.007	-0.064**	1.000	0.013	-0.021	0.015	0.009
Birth Order	0.095**	0.110**	0.013	1.000	-0.013	0.014	0.013
PGS for Time outdoors	0.044	0.080**	-0.021	-0.013	1.000	-0.463**	0.019
PGS for EduYears	-0.068**	-0.076**	0.015	0.014	-0.463**	1.000	0.037
PGS for Sleep Duration	0.010	-0.021	0.009	0.013	0.019	0.037	1.000
** P <0.05 after Bonferroni adjustment to account for multiple testing							

Supplementary Figure S1. Correlation of sleep duration across childhood. Scatter plots of sleep duration between ages 3.5-11.5 years. Spearman correlations are shown in the lower triangle.

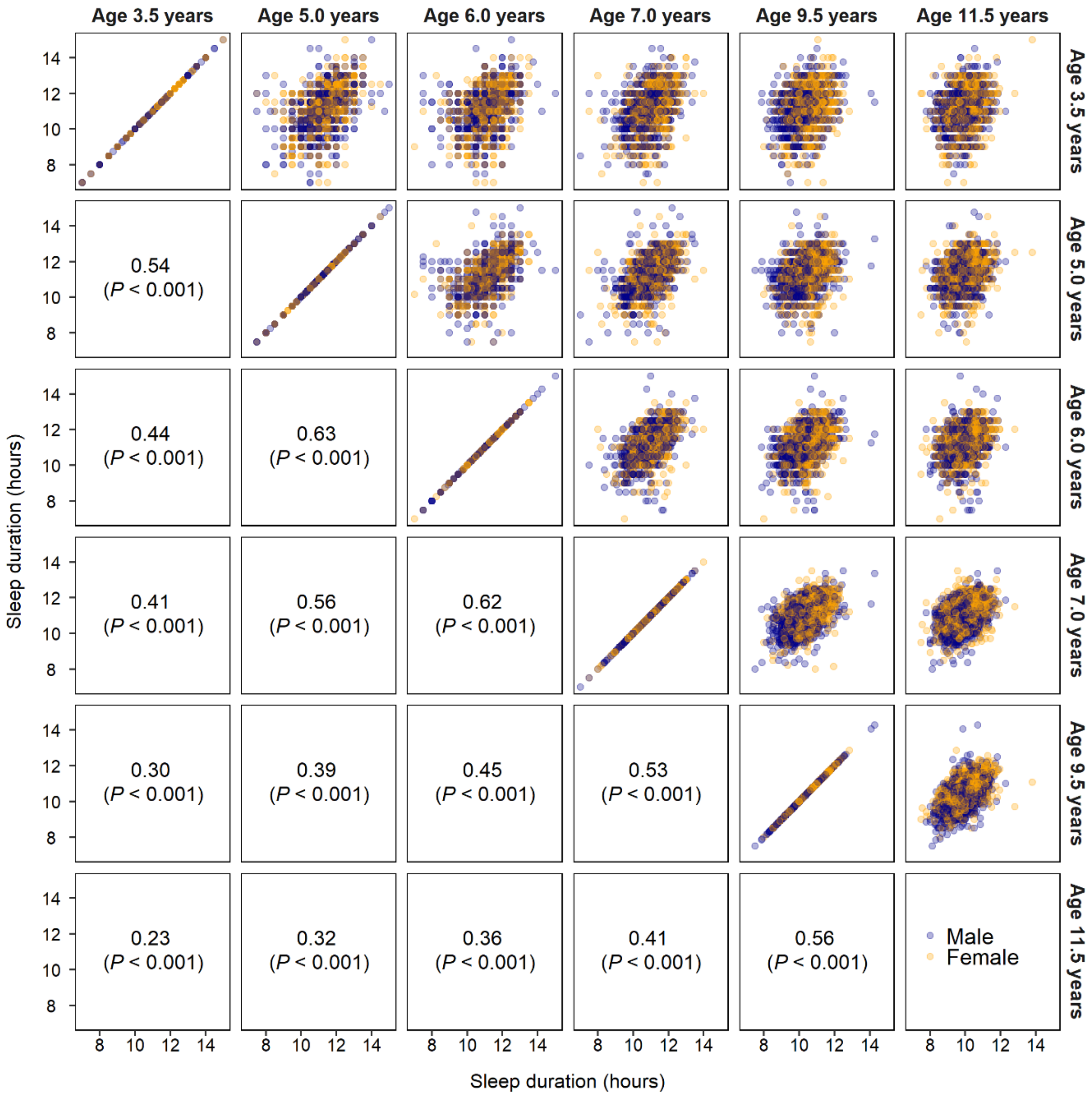

Supplementary Figure S2. Correlation of time outdoors across childhood. Scatter plots of parental-reported time outdoors between ages 3-14 years. Spearman correlations are shown in the lower triangle.

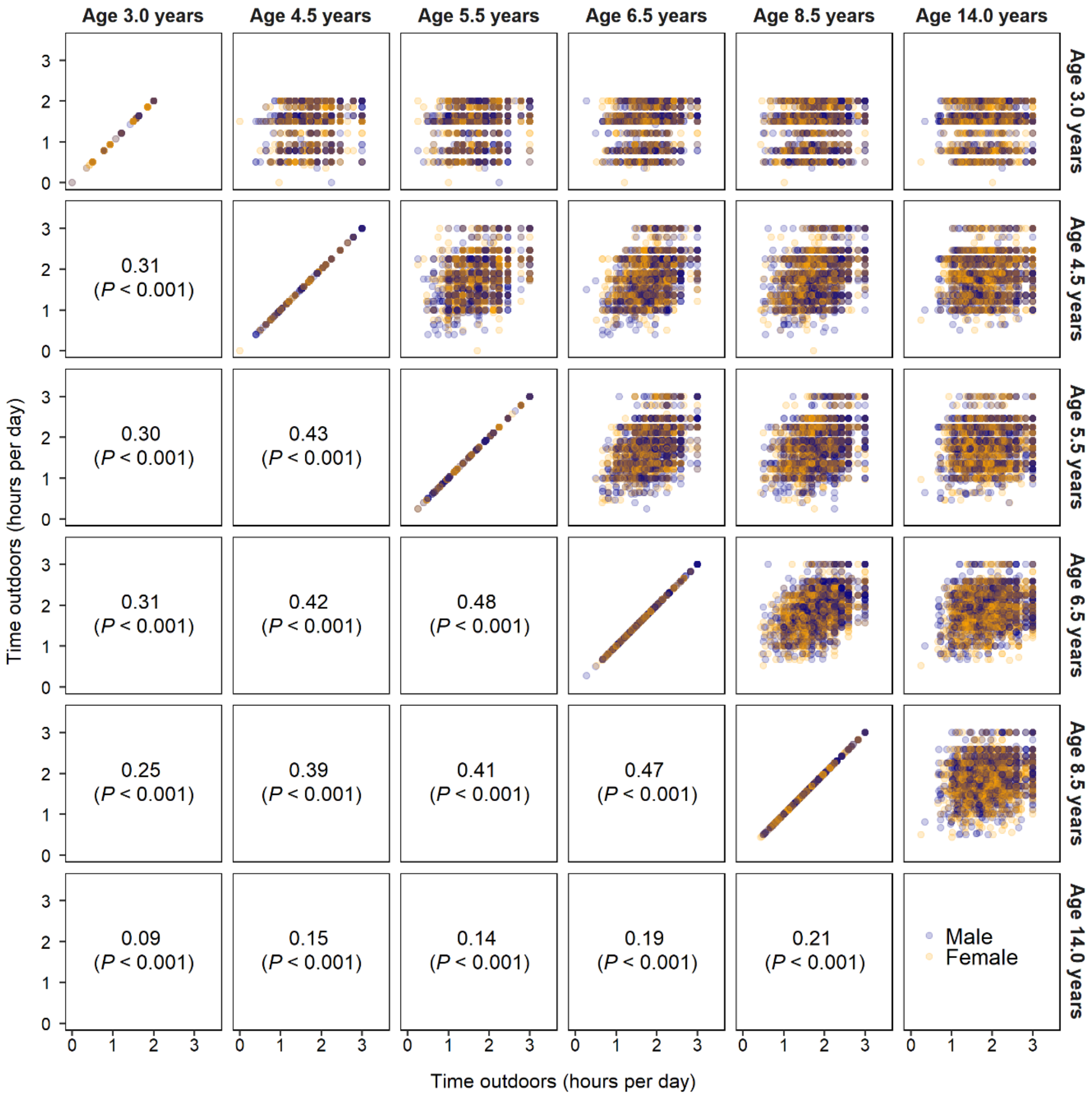

Supplementary Figure S3. Correlation of time reading across childhood. Scatter plots of parental-reported time reading between ages 4.5-14.0 years. Spearman correlations are shown in the lower triangle. Points were jittered by 0.2 hours/day to better illustrate trends.

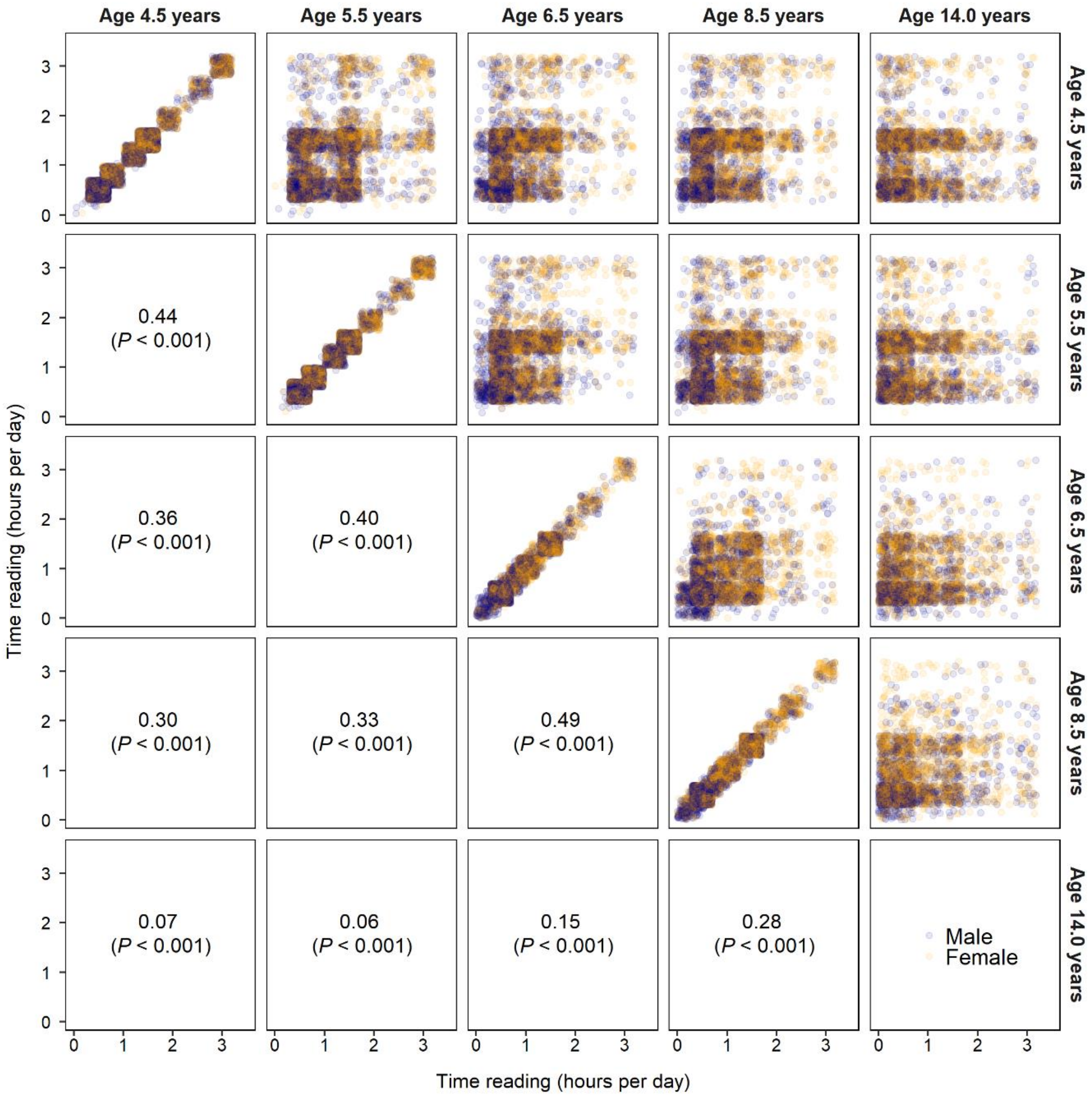

Supplementary Figure S4. Observational associations of sleep duration, time outdoors and time reading at early ages with the outcome, 'refractive error at age 15 years'. Activities (in units of hours per day) were recorded via questionnaires between the ages of 3 and 14 years. Associations are presented in units of D per one hour of additional activity each day. N is the sample size for the analysis. Analyses were adjusted for gender, maternal age and Townsend Deprivation Index (quintile).

Activity (hours per day)	Age (years)	N	Refractive error in diopters (95% CI)
Sleep duration			
	3.0	3292	0.07 (0.02 to 0.12)
	4.5	3255	0.05 (-0.02 to 0.11)
	5.5	3137	0.01 (-0.05 to 0.08)
	6.5	3091	-0.02 (-0.09 to 0.06)
	9.5	3196	0.03 (-0.05 to 0.10)
	11.5	3019	-0.02 (-0.10 to 0.06)
Time outdoors			
	3.0	3279	0.08 (-0.03 to 0.20)
	4.5	3203	0.09 (-0.01 to 0.19)
	5.5	3152	0.01 (-0.09 to 0.11)
	6.5	3067	0.09 (-0.03 to 0.20)
	8.5	3112	0.10 (-0.01 to 0.20)
	14.0	2497	0.19 (0.09 to 0.29)
Time reading			
	4.5	3211	-0.02 (-0.09 to 0.06)
	5.5	3161	-0.08 (-0.15 to -0.01)
	6.5	3087	-0.18 (-0.27 to -0.09)
	8.5	3131	-0.20 (-0.28 to -0.12)
	14.0	2785	-0.23 (-0.30 to -0.16)

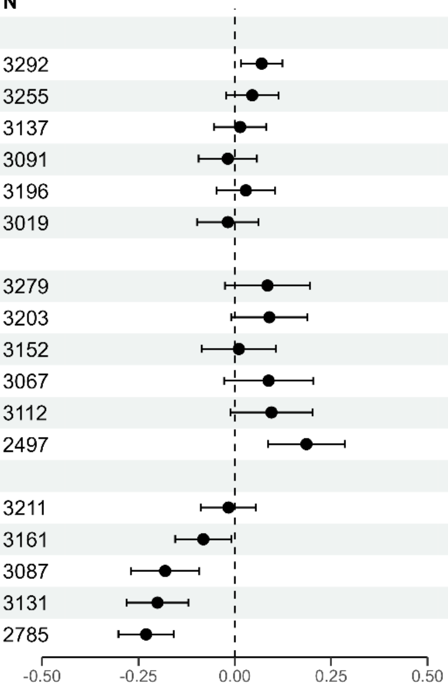

Supplementary Figure S5. Relationship between sleep duration at age 9.5 years with demographic characteristics and instrumental variables. Time outdoors and time reading were reported by parents when the participants were aged 8.5 years. In the upper panels, circle symbols are the mean, the error bars extend for one standard deviation and the shaded region represents the frequency distribution. Δ indicates the difference in sleep duration between groups and the *P*-value is from a *t*-test or ANOVA. In the lower panels, the line is the regression $y \sim x$, while *r* and the *P*-value refer to a Spearman correlation test.

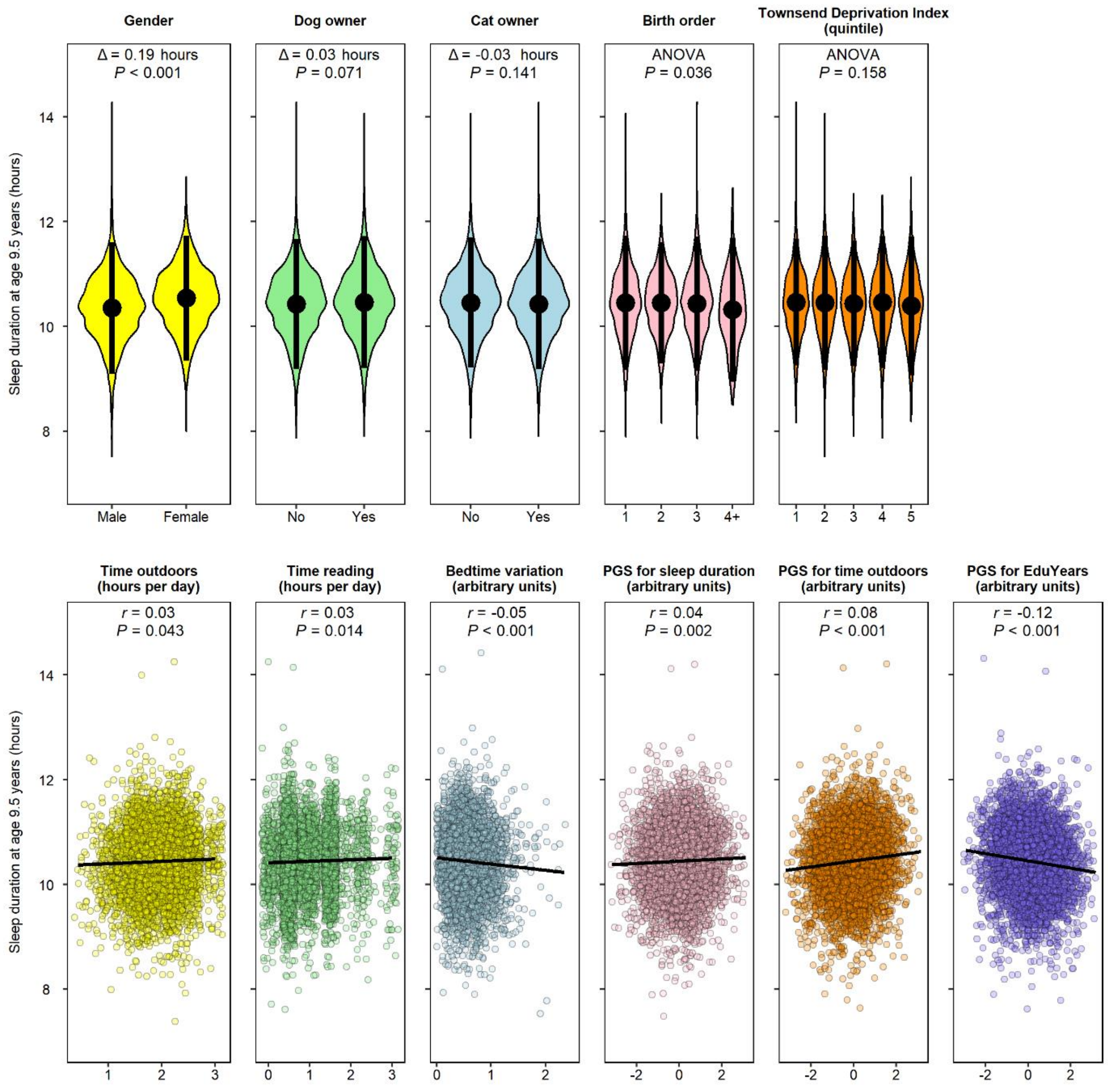

Supplementary Figure S6. Observational associations between seven different instrumental variables and children's sleep duration at various ages. Seven variables were evaluated for their association with the time participants spent sleeping at age 3 to 11.5 years. Associations are presented in units of 'hours of sleep' per unit of the test variable in a univariate analysis. N is the sample size for the analysis. Units were as follows: Bedtime variation in standard deviation of child's bedtime between 3.5 and 7 years-old; Birth order in numerical order; Cat owner as Yes vs. No (reference) for parented reported ownership of a cat at any time when child was aged 7-15 years; Dog owner as Yes vs. No (reference) for parented reported ownership of a dog at any time when child was aged 7-15 years; PGS for EduYears/Sleep duration/Time outdoors all in units of standard deviation. (Results are not shown for sleep duration at ages 3.5 to 7 years and Bedtime variation, since the sleep duration bedtime at these ages was used to derive the Bedtime variation variable).

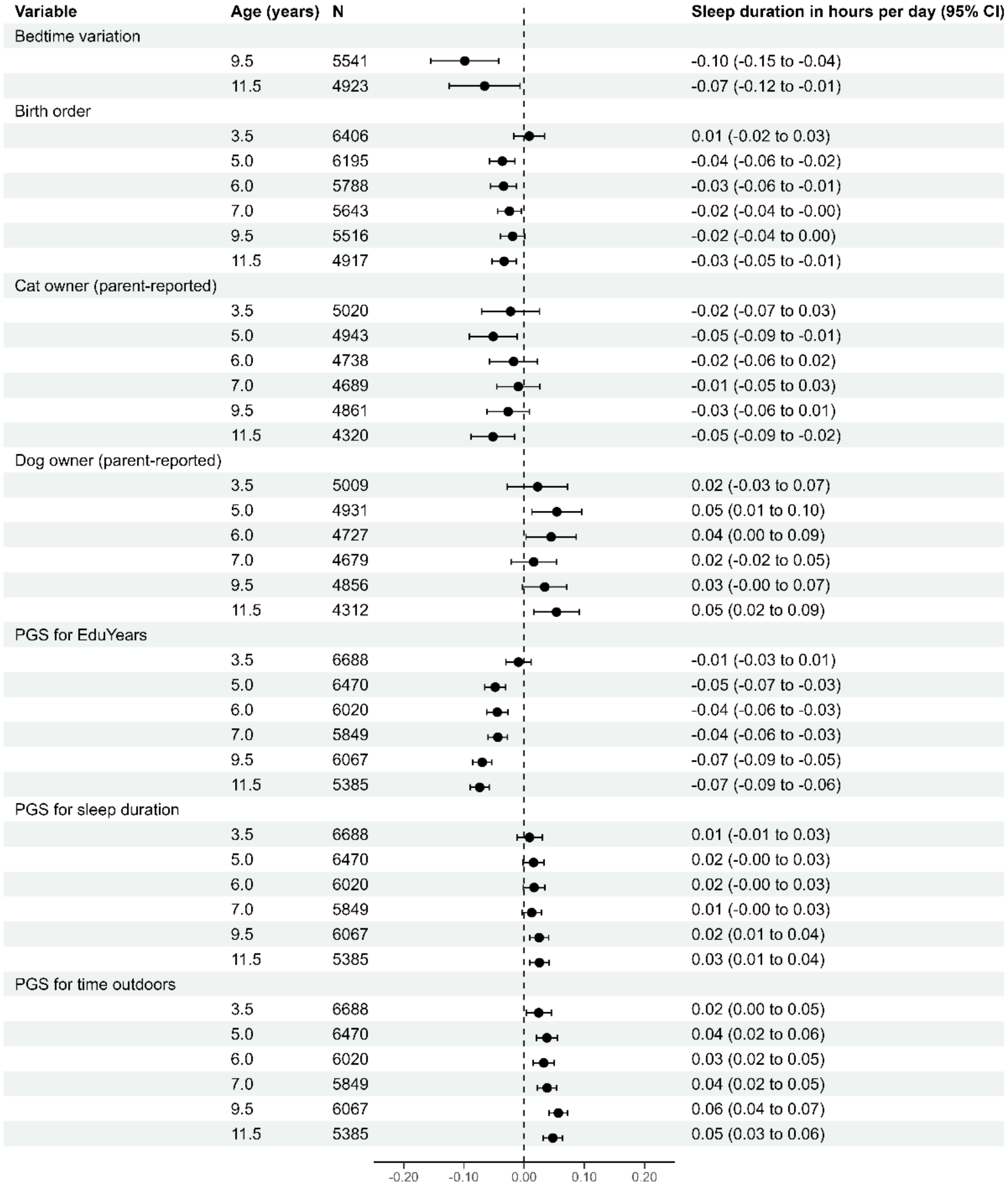

Supplementary Figure S7. Observational associations between seven different instrumental variables and children's time outdoors at various ages. Seven variables were evaluated for their association with the time participants spent outdoors at age 3 to 14 years. Associations are presented in units of hours of daily time spent outdoors per unit of the test variable in a univariate analysis. N is the sample size for the analysis. Units were as follows: Bedtime variation in standard deviation of child's bedtime between 3.5 and 7 years-old; Birth order in numerical order; Cat owner as Yes vs. No (reference) for parented reported ownership of a cat at any time when child was aged 7-15 years; Dog owner as Yes vs. No (reference) for parented reported ownership of a dog at any time when child was aged 7-15 years; PGS for EduYears/Sleep duration/Time outdoors all in units of standard deviation.

Variable	Age (years)	N	Time outdoors in hours per day (95% CI)
Bedtime_variation			
	3.0	6295	0.03 (-0.00 to 0.06)
	4.5	6165	0.02 (-0.02 to 0.06)
	5.5	6034	0.08 (0.04 to 0.12)
	6.5	5761	0.07 (0.03 to 0.11)
	8.5	5432	0.07 (0.03 to 0.11)
	14.0	3759	0.14 (0.08 to 0.20)
Birth order			
	3.0	6401	0.02 (0.01 to 0.03)
	4.5	6139	0.02 (0.01 to 0.04)
	5.5	5932	0.04 (0.02 to 0.05)
	6.5	5621	0.01 (0.00 to 0.03)
	8.5	5383	0.01 (-0.00 to 0.03)
	14.0	3692	0.03 (0.01 to 0.05)
Cat owner (parent-reported)			
	3.0	4993	-0.00 (-0.03 to 0.02)
	4.5	4870	0.01 (-0.02 to 0.03)
	5.5	4817	0.01 (-0.02 to 0.04)
	6.5	4668	-0.00 (-0.03 to 0.02)
	8.5	4706	0.00 (-0.02 to 0.03)
	14.0	3110	-0.01 (-0.05 to 0.03)
Dog owner (parent-reported)			
	3.0	4982	0.05 (0.02 to 0.07)
	4.5	4857	0.06 (0.04 to 0.09)
	5.5	4807	0.06 (0.03 to 0.09)
	6.5	4657	0.04 (0.01 to 0.06)
	8.5	4697	0.07 (0.04 to 0.10)
	14.0	3106	0.07 (0.03 to 0.11)
PGS for EduYears			
	3.0	6686	-0.03 (-0.04 to -0.02)
	4.5	6417	-0.04 (-0.05 to -0.03)
	5.5	6172	-0.03 (-0.04 to -0.02)
	6.5	5831	-0.04 (-0.05 to -0.02)
	8.5	5906	-0.06 (-0.07 to -0.04)
	14.0	3783	-0.07 (-0.09 to -0.05)
PGS for sleep duration			
	3.0	6686	0.00 (-0.01 to 0.01)
	4.5	6417	-0.00 (-0.01 to 0.01)
	5.5	6172	-0.01 (-0.02 to 0.00)
	6.5	5831	-0.00 (-0.01 to 0.01)
	8.5	5906	-0.01 (-0.02 to 0.01)
	14.0	3783	-0.01 (-0.03 to 0.01)
PGS for time outdoors			
	3.0	6686	0.03 (0.02 to 0.04)
	4.5	6417	0.04 (0.03 to 0.05)
	5.5	6172	0.03 (0.02 to 0.04)
	6.5	5831	0.03 (0.02 to 0.04)
	8.5	5906	0.05 (0.04 to 0.06)
	14.0	3783	0.07 (0.05 to 0.09)

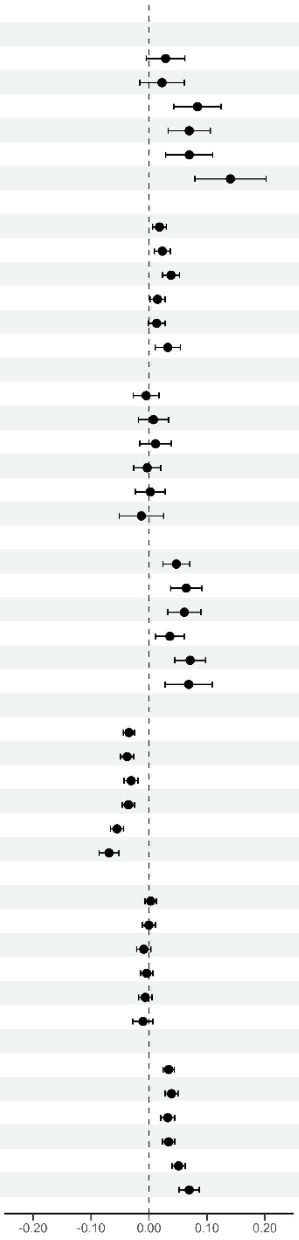

Supplementary Figure S8. Observational associations between seven different instrumental variables and children's time reading at various ages. Seven variables were evaluated for their association with the time participants spent reading at age 4.5 to 14 years. Associations are presented in units of daily hours spent reading per unit of the test variable in a univariate analysis. N is the sample size for the analysis. Units were as follows: Bedtime variation in standard deviation of child's bedtime between 3.5 and 7 years-old; Birth order in numerical order; Cat owner as Yes vs. No (reference) for parented reported ownership of a cat at any time when child was aged 7-15 years; Dog owner as Yes vs. No (reference) for parented reported ownership of a dog at any time when child was aged 7-15 years; PGS for EduYears/Sleep duration/Time outdoors all in units of standard deviation.

Variable	Age (years)	N	Time reading in hours per day (95% CI)
Bedtime_variation			
	4.5	6192	0.07 (0.01 to 0.12)
	5.5	6063	0.07 (0.02 to 0.13)
	6.5	5777	0.05 (0.00 to 0.10)
	8.5	5466	0.01 (-0.05 to 0.06)
	14.0	4088	-0.11 (-0.19 to -0.04)
Birth order			
	4.5	6166	-0.07 (-0.09 to -0.05)
	5.5	5960	-0.06 (-0.08 to -0.04)
	6.5	5640	-0.03 (-0.05 to -0.01)
	8.5	5418	-0.07 (-0.09 to -0.05)
	14.0	4124	-0.06 (-0.09 to -0.03)
Cat owner (parent-reported)			
	4.5	4892	0.01 (-0.03 to 0.05)
	5.5	4835	-0.01 (-0.04 to 0.03)
	6.5	4678	0.00 (-0.03 to 0.03)
	8.5	4740	0.02 (-0.02 to 0.05)
	14.0	3543	0.06 (0.01 to 0.11)
Dog owner (parent-reported)			
	4.5	4879	0.05 (0.01 to 0.09)
	5.5	4825	0.07 (0.04 to 0.11)
	6.5	4668	0.03 (-0.00 to 0.06)
	8.5	4731	0.00 (-0.03 to 0.04)
	14.0	3538	-0.08 (-0.13 to -0.04)
PGS for EduYears			
	4.5	6447	-0.03 (-0.05 to -0.02)
	5.5	6203	-0.03 (-0.05 to -0.02)
	6.5	5850	-0.00 (-0.01 to 0.01)
	8.5	5942	0.04 (0.03 to 0.06)
	14.0	4525	0.14 (0.12 to 0.16)
PGS for sleep duration			
	4.5	6447	-0.03 (-0.04 to -0.01)
	5.5	6203	-0.03 (-0.04 to -0.01)
	6.5	5850	-0.02 (-0.04 to -0.01)
	8.5	5942	-0.03 (-0.05 to -0.02)
	14.0	4525	-0.01 (-0.03 to 0.01)
PGS for time outdoors			
	4.5	6447	0.02 (0.00 to 0.04)
	5.5	6203	0.02 (0.00 to 0.04)
	6.5	5850	-0.00 (-0.02 to 0.01)
	8.5	5942	-0.03 (-0.05 to -0.02)
	14.0	4525	-0.08 (-0.10 to -0.06)

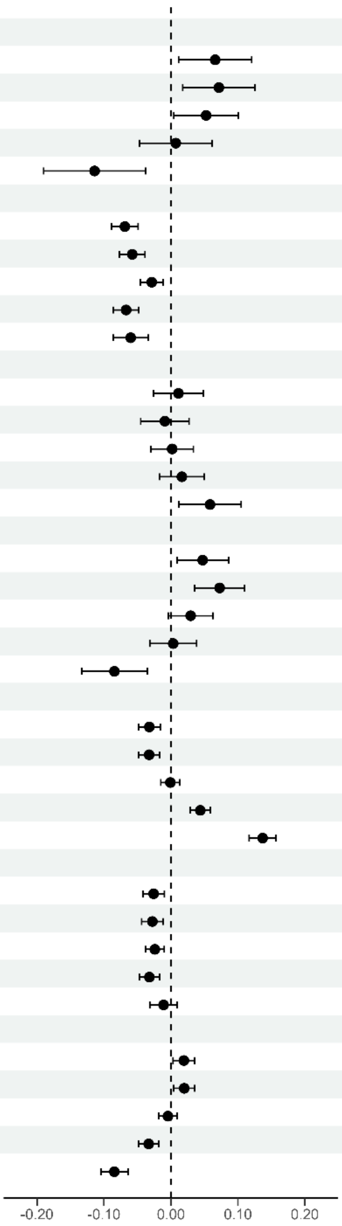

Supplementary Figure S9. Instrumental variable analysis of the relationship between sleep duration and refractive error. Seven variables were considered, one at a time, as an instrumental variable (IV) to gauge the effect of sleep duration when children were aged 9.5 years on 'refractive error at age 15 years' in the cross-sectional sample. Effects are reported in units of diopters per additional hour sleeping each day. N is the sample size for the analysis. F-stat (1st stage) refers to the F-statistic for the first stage of the two step least squares regression, which describes the strength of the relationship between the IV and sleep duration at age 9.5 years. The adjusted analyses included variables for gender, maternal age, Townsend Deprivation Index (quintile), and the first 3 genetic ancestry principal components.

Adjusted	IV	N	F-stat (1st stage)	Refractive error in diopters (95% CI)
Unadjusted analysis				
	Cat owner (parent-reported)	2302	2.01	0.12 (-2.69 to 2.93)
	Dog owner (parent-reported)	2302	6.09	0.32 (-1.31 to 1.95)
	Birth order	2302	0.48	6.09 (-12.04 to 24.23)
	Bedtime_variation	2302	19.48	-0.72 (-1.68 to 0.25)
	PGS for time outdoors	2302	20.54	0.91 (-0.05 to 1.87)
	PGS for EduYears	2302	22.04	1.16 (0.19 to 2.13)
	PGS for sleep duration	2302	1.53	0.96 (-2.58 to 4.49)
Adjusted analysis				
	Cat owner (parent-reported)	2302	2.40	0.05 (-2.56 to 2.66)
	Dog owner (parent-reported)	2302	3.55	0.13 (-2.01 to 2.28)
	Birth order	2302	6.29	2.38 (-0.07 to 4.83)
	Bedtime_variation	2302	24.06	-0.62 (-1.48 to 0.25)
	PGS for time outdoors	2302	13.86	0.97 (-0.23 to 2.17)
	PGS for EduYears	2302	11.17	1.41 (-0.05 to 2.87)
	PGS for sleep duration	2302	1.87	0.94 (-2.30 to 4.17)

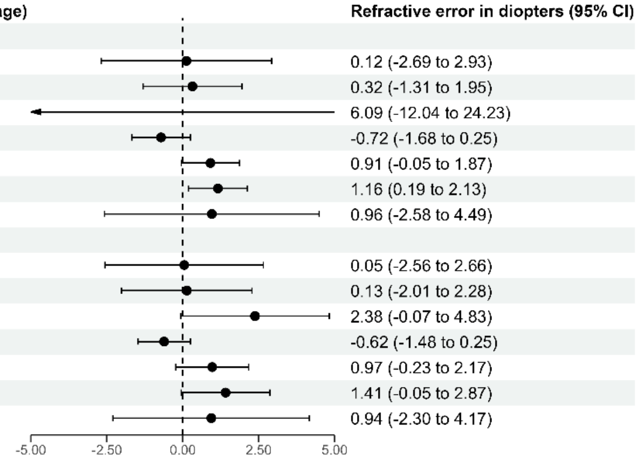

Supplementary Figure S10. Instrumental variable analysis of the relationship between time outdoors and refractive error. Seven variables were considered, one at a time, as an instrumental variable (IV) to gauge the effect of time outdoors when children were aged 8.5 years on 'refractive error at age 15 years' in the cross-sectional sample. Effects are reported in units of diopters per additional hour outdoors each day. N is the sample size for the analysis. F-stat (1st stage) refers to the F-statistic for the first stage of the two step least squares regression, which describes the strength of the relationship between the IV and time outdoors at age 8.5 years. The adjusted analyses included variables for gender, maternal age, Townsend Deprivation Index (quintile), and the first 3 genetic ancestry principal components.

Adjusted	IV	N	F-stat (1st stage)	Refractive error in diopters (95% CI)
Unadjusted analysis				
	Cat owner (parent-reported)	2302	0.10	-0.76 (-19.00 to 17.49)
	Dog owner (parent-reported)	2302	8.37	0.38 (-1.56 to 2.33)
	Birth order	2302	0.00	-84.18 (-2438.81 to 2270.45)
	Bedtime_variation	2302	1.48	3.64 (-3.68 to 10.96)
	PGS for time outdoors	2302	29.97	1.06 (-0.02 to 2.15)
	PGS for EduYears	2302	34.66	1.30 (0.26 to 2.34)
	PGS for sleep duration	2302	0.61	-2.12 (-11.18 to 6.93)
Adjusted analysis				
	Cat owner (parent-reported)	2302	0.14	-0.28 (-15.22 to 14.66)
	Dog owner (parent-reported)	2302	6.08	0.14 (-2.13 to 2.42)
	Birth order	2302	1.57	6.62 (-4.56 to 17.80)
	Bedtime_variation	2302	0.42	6.46 (-14.72 to 27.64)
	PGS for time outdoors	2302	25.81	0.99 (-0.17 to 2.16)
	PGS for EduYears	2302	27.56	1.25 (0.09 to 2.41)
	PGS for sleep duration	2302	0.47	-2.60 (-13.83 to 8.63)

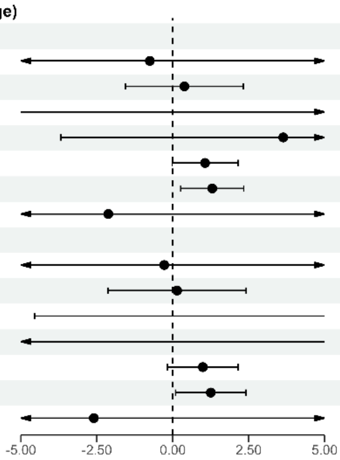

Supplementary Figure S11. Instrumental variable analysis of the relationship between time reading and refractive error. Seven variables were considered, one at a time, as an instrumental variable (IV) to gauge the effect of time reading when children were aged 8.5 years on ‘refractive error at age 15 years’ in the cross-sectional sample. Effects are reported in units of diopters per additional hour spent reading each day. N is the sample size for the analysis. F-stat (1st stage) refers to the F-statistic for the first stage of the two step least squares regression, which describes the strength of the relationship between the IV and time reading at age 8.5 years. The adjusted analyses included variables for gender, maternal age, Townsend Deprivation Index (quintile), and the first 3 genetic ancestry principal components.

Adjusted	IV	N	F-stat (1st stage)	Refractive error in diopters (95% CI)
Unadjusted analysis				
	Cat owner (parent-reported)	2302	0.01	1.57 (-44.93 to 48.06)
	Dog owner (parent-reported)	2302	0.02	-5.83 (-88.15 to 76.49)
	Birth order	2302	30.38	-0.82 (-1.62 to -0.01)
	Bedtime_variation	2302	0.02	-24.90 (-385.52 to 335.72)
	PGS for time outdoors	2302	5.57	-1.86 (-4.13 to 0.41)
	PGS for EduYears	2302	15.34	-1.48 (-2.74 to -0.21)
	PGS for sleep duration	2302	10.47	-0.39 (-1.70 to 0.92)
Adjusted analysis				
	Cat owner (parent-reported)	2302	0.10	0.26 (-13.86 to 14.38)
	Dog owner (parent-reported)	2302	0.12	-0.80 (-13.88 to 12.29)
	Birth order	2302	27.92	-1.21 (-2.11 to -0.31)
	Bedtime_variation	2302	0.08	-11.67 (-95.14 to 71.79)
	PGS for time outdoors	2302	9.09	-1.28 (-2.87 to 0.31)
	PGS for EduYears	2302	27.34	-0.97 (-1.84 to -0.09)
	PGS for sleep duration	2302	11.52	-0.40 (-1.68 to 0.87)

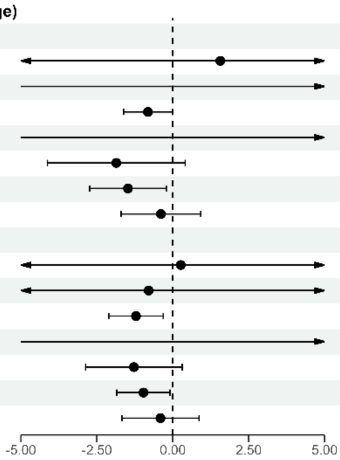

Supplementary Figure S12. Risk factors associated with the outcome 'refractive error at age 15 years' in the cross-sectional sample in analyses using the top three instrumental variables or all seven instrumental variables. Associations are presented in units of D per one hour of additional activity each day. Risk factor exposure (in units of hours per day) was recorded via questionnaires at the specified age. Risk factors were first assessed one at a time. The analyses included either 3 or 7 instrumental variables and adjusted for gender, maternal age, Townsend Deprivation Index, and the first 3 genetic ancestry principal components. F-stat refers to the F-statistic (combined for all instrumental variables) from the first stage of the two step least squares regression. The top three IVs for sleep duration were: bedtime variability, the PGS for time outdoors and the PGS for EduYears. The top three IVs for time outdoors were: dog ownership by the family, the PGS for time outdoors and the PGS for EduYears. The top three IVs for time reading were: birth order, the PGS for EduYears and the PGS for sleep duration.

Exposure	IV	F-stat (1st stage)	Refractive error in diopters (95% CI)
Sleep duration at age 9.5 years			
	Top three IVs	13.97	-0.00 (-0.63 to 0.62)
	All seven IVs	8.41	0.23 (-0.30 to 0.77)
Time outdoors at age 8.5 years			
	Top three IVs	14.07	0.81 (-0.09 to 1.71)
	All seven IVs	6.26	0.94 (0.05 to 1.82)
Time reading at age 8.5 years			
	Top three IVs	23.55	-0.90 (-1.44 to -0.36)
	All seven IVs	10.31	-0.88 (-1.42 to -0.34)

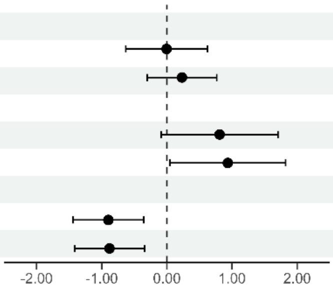

Supplement: Supplement 1 [file tvst-13-11-10_s001.pdf]
